# On the canonical energy of weak gravitational fields with a cosmological constant $\varLambda \in \mathbb {R}$

**DOI:** 10.1140/epjc/s10052-021-09350-y

**Published:** 2021-08-05

**Authors:** P. T. Chruściel, Sk J. Hoque, M. Maliborski, T. Smołka

**Affiliations:** 1 University of Vienna, Gravitational Physics, Boltzmanngasse, 5, A1090 Wien, Austria; 2 Institut de Hautes Études Scientifiques, Le Bois-Marie, 35 rte de Chartres, 91440 Bures-sur-Yvette, France; 3 Institute of Theoretical Physics, Faculty of Mathematics and Physics, Charles University, V Holešovičkách 2, 180 00 Prague 8, Czech Republic; 4 Department of Mathematical Methods in Physics, Faculty of Physics, University of Warsaw, Pasteura 5, 02-093 Warszawa, Poland

## Abstract

We analyse the canonical energy of vacuum linearised gravitational fields on light cones on a de Sitter, Minkowski, and Anti de Sitter backgrounds in Bondi gauge. We derive the associated asymptotic symmetries. When $\varLambda >0$ the energy diverges, but a renormalised formula with well defined flux is obtained. We show that the renormalised energy in the asymptotically off-diagonal gauge coincides with the quadratisation of the generalisation of the Trautman–Bondi mass proposed in Chruściel and Ifsits (Phys Rev D 93:124075, arXiv:1603.07018 [gr-qc], 2016).

## Introduction

A question of current interest is the amount of energy that can be radiated by a gravitating system in the presence of a positive cosmological constant. This problem has been addressed in [13] using an approach based on the characteristic constraint equations and involving Bondi coordinates. The analysis there showed ambiguities in the resulting expression. The question then arises, whether some insight into the problem at hand could be gained by considering linearised gravity on the de Sitter background. The aim of this work is to carry out this project.

We start, in Sect. 2 with a general analysis of the canonical energy in linearised Lagrangian theories. The main point is to derive a formula for the canonical energy including all boundary terms, which are usually neglected and which play a key role in general relativity. The results presented in this section are essentially known [41, 56], but a coherent and systematic presentation for linearised field theories, keeping track of all terms in the integrals, does not exist in the literature. One of the main results in this section is Proposition 1, which does not seem to have appeared in the literature in this generality.

In Sect. 3 we show how to put a linearised gravitational field into Bondi gauge, analyse the small-*r* behaviour of the fields and derive the freedom remaining. This gives a unified treatment for all $\varLambda \in \mathbb {R}$ of asymptotic symmetries *à la Bondi-Metzner-Sachs* in the linearised regime, which leads to a clear view of the differences arising from the sign of the cosmological constant and from the boundary conditions imposed. We note that the current approach to asymptotic symmetries, based on characteristic initial data and their evolution, gives a perspective different from the one based e.g. on Fefferman–Graham expansions, as it introduces naturally a foliation of the conformal boundary $\mathscr {I}$ by spheres obtained by intersecting $\mathscr {I}$ with the light cones.

In Sect. 4 we analyse the large-*r* behaviour of vacuum metric perturbation in the Bondi gauge and show explicitly how the formalism works for a class of linearised solutions of the vacuum Einstein equations discovered by Blanchet and Damour [8]. Our asymptotic conditions on the linearised perturbations of the metric are modelled on the asymptotic behaviour of the full solutions of the Einstein vacuum field equations with positive cosmological constant and with smooth initial Cauchy data on $S^3$, as derived by Friedrich in [29]. Here some comments might be in order. In [29] Friedrich shows that small perturbations of de Sitter Cauchy data on $S^3$ lead to vacuum spacetimes with smooth conformal boundaries at infinity. He isolates a set of data on the spacelike boundary at infinity which parameterise uniquely all vacuum spacetimes with a positive cosmological constant and with smooth conformal completions at infinity. His analysis carries over without difficulties to the linearised equations; a convenient way to proceed is to linearise the equations in [47]. The results of Friedrich provide a rigorous justification of the asymptotic expansions proposed by Starobinsky [50], revisited later from a more general perspective in [26]. The readers familiar with the Fefferman–Graham expansions can view the results in this section as a translation of these expansions to characteristic Cauchy data in the linearised setting.

It should be emphasised that requiring asymptotic conditions more stringent than ours will lead to non-generic fields, in the sense that generic smooth changes of initial data on a Cauchy surface in de Sitter spacetime will lead to solutions which do not satisfy the more stringent conditions. And allowing less stringent conditions will lead to linearised metric fields which are not smoothly conformally extendable along, and in a neighborhood of, the initial light cone. In other words, our asymptotic conditions are optimal for linearised fields which are smoothly conformally extendable.

In Sect. 5 we apply the formalism developed so far to derive our formula for the canonical energy of vacuum linearised metric perturbations on a de Sitter, Minkowski, and Anti de Sitter backgrounds.

In the asymptotically flat case we recover the linearised-theory version of the usual Trautman–Bondi mass. Similarly when $\varLambda \le 0$ and the old-fashioned [4, 5, 16, 32] strong decay conditions are imposed on the linearised field we obtain the linearised version of the usual, conserved, Anti de Sitter mass.

When $\varLambda >0$, or when $\varLambda <0$ but the asymptotic conditions are relaxed to the linearised version of not-conformally-flat-$\mathscr {I}$, we obtain an expression for the canonical mass on light cones truncated to radius *R* which diverges when *R* tends to infinity. It turns out that the divergent part of the energy has a dynamics of its own, which allows one to introduce a renormalised energy which satisfies a well defined flux formula. This formula, and its flux, are the main results of this work, to be found in Sect. 5.4. We show in Sect. 5.6 that the renormalised energy coincides with a quadratisation of a generalisation of the Trautman–Bondi mass proposed in [13] when an “asymptotically off-diagonal gauge” is used. On the other hand, the “asymptotically block-diagonal gauge” leads to a formula for the renormalised energy which differs from the previous one by a boundary term determined in Sect. 5.7.1. We find several natural candidates for a renormalised canonical energy: $\delta ^2 m_{\mathrm {TB}}(\mathscr {C}_{u })$, $\hat{E}_{c,I}$ and $\hat{E}_{c,II}$; yet another one, $\delta ^2 E^{(\varLambda )}$, motivated by holography, has been proposed in [20]; cf. Sect. 5.7.2. We find that the only one which is invariant under asymptotic symmetries is $\delta ^2 m_{\mathrm {TB}}(\mathscr {C}_{u })$, which coincides with $\hat{E}_{c,I}$ in the gauge where the Bondi-transformed linearised metric is as regular as possible at the origin.

Most of the results derived here have been summarised in [12]. Further results on, or related to, this problem can be found in [1–3, 7, 24, 25, 35, 42, 43, 49, 51, 52].

We note that there exists a rich literature on energy when $\varLambda <0$ [4, 6, 19, 31, 33, 40, 48], and while essentially all our results apply to either sign of cosmological constant, including $\varLambda =0$, we will not carry-out a systematic comparison of our results to these, since the main concern of this paper is the case $\varLambda >0$, which is much less understood so far.

## The energy of linearised fields

Our aim in this section is to establish that the canonical energy of the linearised theory can be calculated, up-to-divergence, by means of the “presymplectic current” (using the Lee-Wald terminology) of the original theory; see Proposition 1 below. This is well known, but we revisit the proof as we will need the formula for the divergence term.

We further show gauge-independence up-to-boundary term of the canonical energy of the linearised theory. This is also well known (cf., e.g., [22, 28, 56]), and closely related to the above, but here again our focus is on the explicit form of the boundary term.

### General formalism

We consider a first-order Lagrangian field theory for a collection of fields $\phi \equiv (\phi ^A)$, where *A* runs over a finite set. We write

$$\begin{aligned} \partial \phi \equiv \left( \phi ^A{}_\mu \right) {:}{=} \left( \partial _\mu \phi ^A \right) \equiv \left( \frac{\partial \phi ^A}{\partial x^\mu } \right) \,. \end{aligned}$$

Given a Lagrangian density $\mathscr {L}(\phi , \partial \phi , \cdot )$, where $\cdot$ denotes background fields (which might or might not be present), the field equations are

2.1 $$\begin{aligned} \mathscr {E}_A{:}{=} \partial _\mu \left( \frac{\partial \mathscr {L}}{\partial \phi ^A{}_\mu } \right) - \frac{\partial \mathscr {L}}{\partial \phi ^A} =0 \,. \end{aligned}$$

For $\lambda \in I$, where *I* is an open interval containing 0, let $\phi (\lambda )\equiv \left( \phi ^A(\lambda )\right)$ be a one-parameter family of fields differentiable with respect to $\lambda$, set

2.2 $$\begin{aligned} \tilde{\phi }\equiv (\tilde{\phi }^A) {:}{=} \frac{d \phi ^A}{d\lambda } \,. \end{aligned}$$

Then $\tilde{\phi }$ satisfies the set of equations

2.3 $$\begin{aligned}&\partial _\mu \left( \frac{\partial ^2 \mathscr {L}}{\partial \phi ^A{}_\mu \partial \phi ^B{}_\nu } \partial _\nu \tilde{\phi }^B + \frac{\partial ^2 \mathscr {L}}{\partial \phi ^A{}_\mu \partial \phi ^B } \tilde{\phi }^B \right) \nonumber \\&\quad = \frac{\partial ^2 \mathscr {L}}{ \partial \phi ^A\partial \phi ^B{}_\mu } \partial _\mu \tilde{\phi }^B + \frac{\partial ^2 \mathscr {L}}{ \partial \phi ^A \partial \phi ^B } \tilde{\phi }^B + {{d \mathscr {E}_A}\over {d\lambda }} \,. \end{aligned}$$

To continue, it is convenient to introduce some notation. We set

2.4 $$\begin{aligned}&\displaystyle {\pi }_A{}^\mu {:}{=} \frac{\partial \mathscr {L}}{\partial \phi ^A{}_\mu } \,, \quad {\pi }_A {:}{=} \frac{\partial \mathscr {L}}{\partial \phi ^A } \,, \end{aligned}$$

2.5 $$\begin{aligned}&\displaystyle {\pi }_A{}^\mu {}_B{}^\nu {:}{=} \frac{\partial ^2 \mathscr {L}}{\partial \phi ^A{}_\mu \partial \phi ^B{}_\nu } \,, \ {\pi }_A{}^\mu {}_B {:}{=} \frac{\partial ^2 \mathscr {L}}{\partial \phi ^A{}_\mu \partial \phi ^B } \,, \ {\pi }_{A B} {:}{=} \frac{\partial ^2 \mathscr {L}}{ \partial \phi ^A \partial \phi ^B } \,. \end{aligned}$$

In this notation, (2.3) can be rewritten somewhat more concisely as

2.6 $$\begin{aligned}&\partial _\mu \left( {\pi }_A{}^\mu {}_B{}^\nu \partial _\nu \tilde{\phi }^B + {\pi }_A{}^\mu {}_B \tilde{\phi }^B \right) \nonumber \\&\quad = \left( {\pi }_B{}^\mu {}_A \partial _\mu \tilde{\phi }^B + {\pi }_{A B} \tilde{\phi }^B \right) + {{d \mathscr {E}_A}\over {d\lambda }} \,. \end{aligned}$$

*If* ${{d \mathscr {E}_A}\over {d\lambda }}$ *has been prescribed* (e.g., equal to zero, in which case the fields $\tilde{\phi }^A$ satisfy the linearised field equations), or is known and is $\tilde{\phi }$–independent, this set of equations can be derived from a Lagrangian density ${\,\,\,\widetilde{\mathscr {L}}}$ given by

2.7 $$\begin{aligned} {\,\,\,\widetilde{\mathscr {L}}}= & {} \frac{1}{2} {\pi }_A{}^\mu {}_B{}^\nu \partial _\mu \tilde{\phi }^A \partial _\nu \tilde{\phi }^B + {\pi }_A{}^\mu {}_B \partial _\mu \tilde{\phi }^A \tilde{\phi }^B \nonumber \\&+ \frac{1}{2} {\pi }_{A B} \tilde{\phi }^A \tilde{\phi }^B + {{d \mathscr {E}_A}\over {d\lambda }} \tilde{\phi }^A. \end{aligned}$$

(To avoid ambiguities: we see that (2.7) with ${{d \mathscr {E}_A}\over {d\lambda }} \equiv 0$ provides the Lagrangian for the linearised field equations at $\phi (\lambda )|_{\lambda =0}$, regardless of whether or not $\phi (\lambda )|_{\lambda =0}$ itself satisfies any field equations, though typically one would be interested in situations where $\mathscr {E}_A({\phi (0)})=0$.)

We remark that the field $\phi {:}{=}\phi (\lambda )|_{\lambda = 0}$ plays a role of a background field in ${\,\,\,\widetilde{\mathscr {L}}}$, so even if we assumed that $\mathscr {L}$ depends only upon $\phi$ and $\partial \phi$, we end up with a Lagrangian density where background dependence has to be taken into consideration.

The following identities are useful, assuming ${{d \mathscr {E}_A}\over {d\lambda }}=0$:

2.8 $$\begin{aligned} \tilde{{\pi }}_A{}^\mu (\tilde{\phi })&{:}{=}\, \frac{\partial {\,\,\,\widetilde{\mathscr {L}}}}{\partial \tilde{\phi }^A{}_\mu } = {\pi }_A{}^\mu {}_B{}^\nu \partial _\nu \tilde{\phi }^B + {\pi }_A{}^\mu {}_B \tilde{\phi }^B\nonumber \\&= \frac{d \big (\frac{\partial \mathscr {L}}{\partial \phi _A{}^\mu } \big )}{d\lambda } = \frac{d {\pi }_A{}^\mu }{d\lambda } \,, \end{aligned}$$

2.9 $$\begin{aligned} \tilde{{\pi }}_B(\tilde{\phi })&{:}{=} \frac{\partial {\,\,\,\widetilde{\mathscr {L}}}}{\partial \tilde{\phi }^B } = {\pi }_A{}^\mu {}_B \partial _\mu \tilde{\phi }^A + {\pi }_{A B} \tilde{\phi }^A\nonumber \\&= \frac{d \big (\frac{\partial \mathscr {L}}{\partial \phi _B} \big )}{d\lambda } = \frac{d {\pi }_B }{d\lambda } \,. \end{aligned}$$

From now on, we consider a theory which satisfies the following:

1. $\mathscr {L}$ is a scalar density.
2. There exists a notion of derivation with respect to a family of vector fields *X*, which we will denote by ${\mathcal {L}}_X$, which coincides with the usual Lie derivative on vector densities, and which we will call *Lie derivative* regardless of whether or not this is the usual Lie derivative on the remaining fields, such that the following holds:
  - ${\mathcal {L}}_X$ preserves the type of a field, thus ${\mathcal {L}}_X$ of a scalar density is a scalar density, etc.;
  - the field $\pi _A{}^\mu {\mathcal {L}}_X \phi ^A$ is a vector density;
  - in a coordinate system in which $X={\partial _0}$ we have ${\mathcal {L}}_X= {\partial _0}$;
  - ${\mathcal {L}}_X$ satisfies the Leibnitz rule.

The above holds if the fields $\phi ^A$ are tensor fields and $\mathscr {L}$ is of the form $\sqrt{|\det g|} L$, where *L* is a scalar, with ${\mathcal {L}}$ the standard Lie derivative.

Let us denote by ${\,\,\,\widetilde{\mathscr {H}}}$ the Hamiltonian density vector (called “Noether current” in [45]) associated with the Lagrangian density ${\,\,\,\widetilde{\mathscr {L}}}$ and *X*:

2.10 $$\begin{aligned} {\,\,\,\widetilde{\mathscr {H}}}^\mu [X]&{:}{=}\, \frac{\partial {\,\,\,\widetilde{\mathscr {L}}}}{\partial \tilde{\phi }^A{}_\mu } {\mathcal {L}}_X \tilde{\phi }^A - X^\mu {\,\,\,\widetilde{\mathscr {L}}}\nonumber \\&= \left( {\pi }_A{}^\mu {}_B{}^\nu \partial _\nu \tilde{\phi }^B + {\pi }_A{}^\mu {}_B \tilde{\phi }^B \right) {\mathcal {L}}_X \tilde{\phi }^A - X^\mu {\,\,\,\widetilde{\mathscr {L}}}\,. \end{aligned}$$

Let $\mathscr {H}^\mu [X]$ be the corresponding vector density associated with the original field $\phi$:

2.11 $$\begin{aligned} \mathscr {H}^\mu [X]&{:}{=}\, \frac{\partial \mathscr {L}}{\partial \phi ^A{}_\mu } {\mathcal {L}}_X \phi ^A - X^\mu \mathscr {L}\nonumber \\&= {\pi }_A{}^\mu {\mathcal {L}}_X \phi ^A - X^\mu \mathscr {L}\,. \end{aligned}$$

We wish to calculate the variation of $\mathscr {H}^\mu$. Typically one assumes that the background fields, if any, are $\lambda$-independent. This might, however, not be the case for some variations, e.g. if the variations correspond to coordinate transformation which do not leave the background invariant. In order to allow for such situations let us denote by

$$\begin{aligned} \psi {:}{=} (\psi ^I) \end{aligned}$$

the collection of all background fields; if $\mathscr {L}$ depends both upon a background and some derivatives thereof, we include the derivatives of the background as part of components of $\psi$. For completeness we will carry out the usual calculation of $\frac{d \mathscr {H}^\mu [X]}{d\lambda }$. For this, recall the formula for the Lie derivative of a vector density *Z*:

2.12 $$\begin{aligned} {\mathcal {L}}_X Z^\mu = \partial _\sigma (X^\sigma Z^\mu ) - Z^\sigma \partial _\sigma X^\mu \,. \end{aligned}$$

Keeping in mind that ${\pi }_A{}^\mu$ is a tensor density by H1), and our remaining hypotheses H2.a)-H2.d), we are led to the following identity:

2.13 $$\begin{aligned} \partial _\sigma \left( X^\sigma {\pi }_A{}^\mu {{d \phi ^A}\over {d\lambda }}\right)= & {} {\mathcal {L}}_X {\pi }_A{} ^\mu {{d \phi ^A}\over {d\lambda }} + {\pi }_A{} ^\mu {\mathcal {L}}_X {{d \phi ^A}\over {d\lambda }}\nonumber \\&+ \partial _\sigma X^\mu {\pi }_A{}^\sigma {{d \phi ^A}\over {d\lambda }} \,. \end{aligned}$$

We are ready now to calculate the variation of $\mathscr {H}$:

2.14 $$\begin{aligned}&\frac{d \mathscr {H}^\mu [X]}{d\lambda }\nonumber \\&\quad = {\mathcal {L}}_X \phi ^A \frac{d {\pi }_A{}^\mu }{d\lambda } + {\pi }_A{}^\mu {\mathcal {L}}_X \frac{d \phi ^A}{d\lambda } \nonumber \\&\qquad + \underbrace{ {\pi }_A{}^\mu {\mathcal {L}}_{\frac{dX}{d\lambda }} \phi ^A - \frac{dX^\mu }{d\lambda } \mathscr {L}}_{= \mathscr {H}^\mu [ \frac{dX}{d\lambda }]}\nonumber \\&\qquad - X^\mu \bigg ( {\pi }_A{}^\sigma \partial _\sigma {{d \phi ^A}\over {d\lambda }} +\underbrace{\frac{\partial \mathscr {L}}{ \partial \phi ^A} }_{=\partial _\sigma {\pi }_A{}^\sigma - \mathscr {E}^A } {{d \phi ^A}\over {d\lambda }} + \frac{\partial \mathscr {L}}{ \partial \psi ^I}{{{d \psi ^I}\over {d\lambda }}}\bigg ) \nonumber \\&\quad = {\mathcal {L}}_X \phi ^A \frac{d {\pi }_A{}^\mu }{d\lambda } - {\mathcal {L}}_X {\pi }_A{}^\mu \frac{d \phi ^A}{d\lambda } \nonumber \\&\qquad + \big ({\mathcal {L}}_X {\pi }_A{}^\mu -\partial _\sigma (X^\mu {\pi }_A{}^\sigma ) \big ) \frac{d \phi ^A}{d\lambda } \nonumber \\&\qquad + {\pi }_A{}^{\mu }{\mathcal {L}}_X \frac{d \phi ^A}{d\lambda } + \partial _\sigma X^\mu {\pi }_A{}^\sigma {{d \phi ^A}\over {d\lambda }}\nonumber \\&- X^\mu {\pi }_A{}^\sigma \partial _\sigma {{d \phi ^A}\over {d\lambda }} + \mathscr {H}^\mu \left[ \frac{dX}{d\lambda }\right] \nonumber \\&\qquad + X^\mu \left( \mathscr {E}_A \frac{d \phi ^A}{d\lambda } - \frac{\partial \mathscr {L}}{ \partial \psi ^I}{{{d \psi ^I}\over {d\lambda }}} \right) \nonumber \\&\quad = {\mathcal {L}}_X \phi ^A \frac{d {\pi }_A{}^\mu }{d\lambda } - {\mathcal {L}}_X {\pi }_A{}^\mu \frac{d \phi ^A}{d\lambda } \nonumber \\&\qquad + 2 \partial _{{\sigma }} \left( X^{[{{\sigma }}} {\pi }_A{}^{\mu ]} \frac{d \phi ^A}{d\lambda } \right) \nonumber \\&\qquad + \mathscr {H}^\mu \left[ \frac{dX}{d\lambda }\right] + X^\mu \left( \mathscr {E}_A \frac{d \phi ^A}{d\lambda } - \frac{\partial \mathscr {L}}{ \partial \psi ^I}{{{d \psi ^I}\over {d\lambda }}} \right) , \end{aligned}$$

(One should keep in mind that, when the background is not invariant under the flow of *X*, there might be a contribution from the background when calculating ${\mathcal {L}}_X {\pi }_A{}^\mu$.) When integrated over a compact hypersurface $\mathscr {S}$ with boundary,

2.15 $$\begin{aligned} \mathscr {H}[\mathscr {S},X] {:}{=} \int _\mathscr {S}\mathscr {H}^\mu [X] dS_\mu \,, \end{aligned}$$

(2.14) leads to the usual field-theoretical version of the generating formula of Hamilton: Indeed, for solutions of the field equations and for $\lambda$-independent vector fields *X* and background fields the last line in (2.14) vanishes and, using the notation

2.16 $$\begin{aligned}&\delta \phi ^A {:}{=} \frac{d \phi ^A}{d\lambda } \,, \quad \delta {\pi }_A{}^{\mu } {:}{=} \frac{d{\pi }_A{}^{\mu }}{d\lambda } \,, \end{aligned}$$

2.17 $$\begin{aligned}&dS_\mu = \partial _\mu \rfloor dx^{0}\wedge \cdots \wedge dx^n\,, \quad dS_{\mu \nu } = -\partial _\mu \rfloor dS_\nu \,, \end{aligned}$$

after integration of (2.14) over $\mathscr {S}$ one obtains

2.18 $$\begin{aligned} \delta \mathscr {H}[\mathscr {S},X]&{:}{=} \int _\mathscr {S}\frac{d \mathscr {H}^\mu [X]}{d\lambda } dS_\mu \nonumber \\= & {} \int _\mathscr {S}\big ( {\mathcal {L}}_X \phi ^A \delta {\pi }_A{}^\mu - {\mathcal {L}}_X {\pi }_A{}^\mu \delta \phi ^A \big ) dS_\mu \nonumber \\&- \int _{\partial \mathscr {S}} X^{[{{\sigma }}} {\pi }_A{}^{\mu ]} \delta \phi ^A \, dS_{{{\sigma }} \mu } \,, \end{aligned}$$

where the boundary term might or might not vanish depending upon the boundary conditions satisfied by the fields at hand. These terms do not vanish, and play a key role for the problems at hand in this work.

Given two one-parameter families of fields $\phi ^A(\lambda )$ and $\phi ^A( \tau )$, the “presymplectic current” $\omega ^\mu$ is defined as

2.19 $$\begin{aligned} \omega ^\mu \left( \frac{d \phi }{d\lambda }, \frac{d \phi }{d\tau }\right)= & {} \frac{d \phi ^A }{ d\tau } \frac{d {\pi }_A{}^\mu }{d\lambda }- \frac{d \phi ^A }{d\lambda } \frac{d {\pi }_A{}^\mu }{d\tau }\nonumber \\\equiv & {} \frac{d \phi ^A }{ d\tau } \tilde{{\pi }}_A{}^\mu \left( \frac{d \phi }{d\lambda }\right) - \frac{d \phi ^A }{d\lambda } \tilde{{\pi }}_A{}^\mu \left( \frac{d \phi }{d\tau } \right) ,\nonumber \\ \end{aligned}$$

with a similar definition for $\tilde{\omega }^\mu$:

2.20 $$\begin{aligned} \tilde{\omega }^\mu \left( \frac{d \tilde{\phi }}{d{{\sigma }}}, \frac{d \tilde{\phi }}{d\tau }\right)= & {} \frac{d \tilde{\phi }^A }{ d\tau } \frac{d \tilde{{\pi }}_A{}^\mu }{d{{\sigma }}}- \frac{d \tilde{\phi }^A }{d{{\sigma }}} \frac{d \tilde{{\pi }}_A{}^\mu }{d\tau }\nonumber \\= & {} \frac{d \tilde{\phi }^A }{ d\tau } \tilde{{\pi }}_A{}^\mu \left( \frac{d \tilde{\phi }}{d\sigma }\right) - \frac{d \tilde{\phi }^A }{d\sigma } \tilde{{\pi }}_A{}^\mu \left( \frac{d \tilde{\phi }}{d\tau } \right) ,\nonumber \\ \end{aligned}$$

where in the last equation we have used linearity of $\tilde{{\pi }}_A{}^\mu$ in its argument. Strictly speaking, $\tilde{{\pi }}_A{}^\mu \left( \frac{d \tilde{\phi }}{d\sigma }\right)$ in (2.20) should be written as $(\tilde{{\pi }}_A{}^\mu )_*\left( \frac{d \tilde{\phi }}{d\sigma }\right)$, where $(\tilde{{\pi }}_A{}^\mu )_*$ is the map tangent to the linear map

$$\begin{aligned} \frac{d \phi }{d\lambda } \mapsto \tilde{{\pi }}_A{}^\mu \left( \frac{d \phi }{d\lambda } \right) \,, \end{aligned}$$

but we will stick to the notation $\tilde{{\pi }}_A{}^\mu$.

While $\tilde{\omega }^\mu$ and $\omega ^\mu$ look identical, one should keep in mind that they are not defined on the same spaces: the arguments of $\omega ^\mu$ are sections of the bundle tangent to the bundle of fields, while the arguments of $\tilde{\omega }^\mu$ are sections of the bundle of tangents to the tangents. The difference is, however, somewhat esoteric in any case.

#### The divergence of the presymplectic current

We wish to calculate the divergence of the pre-symplectic current (2.19). Consider thus, as before, a two-parameter family of fields $\phi ^{A}(\lambda , \tau )$. Assuming that the variations and the coordinate derivatives commute we have:

2.21 $$\begin{aligned} \partial _{\mu } \left( \frac{d \phi ^A }{ d\tau } \frac{d {\pi }_A{}^\mu }{d\lambda } \right)= & {} \partial _{\mu }\frac{d \phi ^A }{ d\tau } \frac{d {\pi }_A{}^\mu }{d\lambda }+\frac{d \phi ^A }{ d\tau } \partial _{\mu } \frac{d {\pi }_A{}^\mu }{d\lambda } \nonumber \\= & {} \partial _{\mu }\frac{d \phi ^A }{ d\tau } \frac{d {\pi }_A{}^\mu }{d\lambda }+\frac{d \phi ^A }{ d\tau } \frac{d }{d\lambda } \left( \mathscr {E}_A+{\pi }_{A}\right) \nonumber \\= & {} \frac{d}{d \lambda } \left[ {\pi }_A{}^{\mu } \partial _{\mu }\frac{d \phi ^A}{d \tau } +\left( \mathscr {E}_A+{\pi }_{A}\right) \frac{d \phi ^A}{d \tau } \right] \nonumber \\&-{\pi }_A{}^{\mu } \partial _{\mu }\frac{d^2 \phi ^A}{d \tau d \lambda } -\left( \mathscr {E}_A+{\pi }_{A}\right) \frac{d^2 \phi ^A}{d \tau d \lambda } \nonumber \\= & {} \frac{d^2 \mathscr {L}}{d \lambda \delta \tau } +\frac{d}{d \lambda } \left[ \mathscr {E}_A \frac{d \phi ^A}{d \tau } \right] \nonumber \\&-{\pi }_A{}^{\mu } \partial _{\mu }\frac{d^2 \phi ^A}{d \tau d \lambda } -\left( \mathscr {E}_A+{\pi }_{A}\right) \frac{d^2 \phi ^A}{d \tau d \lambda }.\nonumber \\ \end{aligned}$$

Changing the order of $\tau$ and $\lambda$ leads to

2.22 $$\begin{aligned} \partial _{\mu } \omega ^{\mu }\equiv & {} \partial _{\mu } \left( \frac{d \phi ^A }{ d\tau } \frac{d {\pi }_A{}^\mu }{d\lambda }- \frac{d \phi ^A }{d\lambda } \frac{d {\pi }_A{}^\mu }{d\tau } \right) \nonumber \\= & {} \frac{d \mathscr {E}_A}{d \lambda } \frac{d \phi ^A}{d \tau } -\frac{d \mathscr {E}_A}{d \tau } \frac{d \phi ^A}{d \lambda }. \end{aligned}$$

We conclude that the divergence will vanish if the linearised field equations

2.23 $$\begin{aligned} \frac{d \mathscr {E}_A}{d \lambda }= 0 = \frac{d \mathscr {E}_A}{d \tau } \,. \end{aligned}$$

are satisfied.

#### The canonical energy of the linearised theory and the presymplectic form

Calculating directly from the definition (2.11) we have

2.24 $$\begin{aligned} \frac{d \mathscr {H}^\mu [X]}{d\lambda }= & {} \tilde{{\pi }}_A{}^\mu {\mathcal {L}}_X \phi ^A +{\pi }_A{}^\mu {\mathcal {L}}_X \tilde{\phi }^A - X^\mu \nonumber \\&\times \left( {\pi }_A{}^{{\sigma }} \partial _{{\sigma }} \tilde{\phi }^A + {\pi }_A \tilde{\phi }^A +\frac{\partial \mathscr {L}}{ \partial \psi ^I}{{{d \psi ^I}\over {d\lambda }}} \right) \nonumber \\&+\underbrace{{\pi }_A{}^\mu {\mathcal {L}}_{\frac{d X}{d \lambda }} \phi ^A -\frac{d X^{\mu }}{d \lambda } \mathscr {L}}_{\mathscr {H}^\mu [\frac{d X}{d \lambda }]} \,. \end{aligned}$$

Comparing (2.24) with (2.14) we obtain

2.25 $$\begin{aligned} 0= & {} -\pi _A{}^\mu {\mathcal {L}}_X \tilde{\phi }^A - {\mathcal {L}}_X \pi _A{}^\mu \tilde{\phi }^A + 2 \partial _{{\sigma }} \Big ( X^{[\sigma } \pi _A{}^{\mu ]} \tilde{\phi }^A \Big ) \nonumber \\&+ X^{\mu }\big ( {\pi }_{A}{}^{\sigma } \partial _\sigma \tilde{\phi }^A + ({\pi }_A + \mathscr {E}_A)\tilde{\phi }^A \big ) \,. \end{aligned}$$

This is true for all fields $\phi$, $\tilde{\phi }$ and *X*, regardless of whether or not the fields satisfy any equations.

We will differentiate (2.25) with respect to $\lambda$. Before doing this, we note first that a replacement in (2.25) of *X* by $dX/d\lambda$ gives the identity

2.26 $$\begin{aligned} 0= & {} -\pi _A{}^\mu {\mathcal {L}}_{\frac{dX}{d\lambda }} \tilde{\phi }^A - {\mathcal {L}}_{\frac{dX}{d\lambda }} \pi _A{}^\mu \tilde{\phi }^A + 2 \partial _{\sigma } \left( {\frac{dX}{d\lambda }}^{[\sigma } \pi _A{}^{\mu ]} \tilde{\phi }^A \right) \nonumber \\&+ {\frac{dX}{d\lambda }}^{\mu }\left( {\pi }_{A}{}^{\sigma } \partial _\sigma \tilde{\phi }^A + ({\pi }_A + \mathscr {E}_A)\tilde{\phi }^A \right) . \end{aligned}$$

Similarly, replacing $\tilde{\phi }$ by $d\tilde{\phi }/d\lambda$ in (2.25) gives

2.27 $$\begin{aligned} 0= & {} -\pi _A{}^\mu {\mathcal {L}}_{X} \frac{d\tilde{\phi }^A}{d\lambda } - {\mathcal {L}}_{X} \pi _A{}^\mu \frac{d\tilde{\phi }^A}{d\lambda } + 2 \partial _{\sigma } \left( X^{[\sigma } \pi _A{}^{\mu ]} \frac{d\tilde{\phi }^A}{d\lambda } \right) \nonumber \\&+ {X}^{\mu }\left( {\pi }_{A}{}^{\sigma } \partial _\sigma \frac{d\tilde{\phi }^A}{d\lambda } + ({\pi }_A + \mathscr {E}_A)\frac{d\tilde{\phi }^A}{d\lambda } \right) \,. \end{aligned}$$

Differentiating (2.25) with respect to $\lambda$, after taking into account (2.26) and (2.27) one is led to

2.28 $$\begin{aligned} 0= & {} -\tilde{{\pi }}_A{}^\mu {\mathcal {L}}_X \tilde{\phi }^A - {\mathcal {L}}_X \tilde{{\pi }}_A{}^\mu \tilde{\phi }^A + 2 \partial _{{\sigma }} \left( X^{[\sigma } \tilde{{\pi }}_A{}^{\mu ]} \tilde{\phi }^A \right) \nonumber \\&+ X^{\mu }\left( \tilde{{\pi }}_{A}{}^{\sigma } \partial _\sigma \tilde{\phi }^A + \left( \tilde{{\pi }}_A + \frac{d\mathscr {E}_A}{d\lambda }\right) \tilde{\phi }^A \right) . \end{aligned}$$

Adding this to twice the right-hand side of (2.10) one obtains

$$\begin{aligned} 2 {\,\,\,\widetilde{\mathscr {H}}}[X]= & {} \underbrace{ {\mathcal {L}}_X \tilde{\phi }^A \, \tilde{{\pi }}_A{}^\mu - {\mathcal {L}}_X \tilde{{\pi }}_A{}^\mu \tilde{\phi }^A }_{\equiv \, \omega ^\mu (\tilde{\phi }, {\mathcal {L}}_X \tilde{\phi })} + 2 \partial _{{\sigma }} \Big ( X^{[{{\sigma }}} \tilde{{\pi }}_A{}^{\mu ]} \tilde{\phi }^A \Big )\\&- X^\mu \frac{d \mathscr {E}_A}{d \lambda } \tilde{\phi }^A \,. \end{aligned}$$

This leads us to the following (compare [36, Appendix]); note the we are not assuming that the fields $\phi$ at which we are linearising satisfy any equations, nor that the background structures (if any) are invariant under the flow of *X*:

##### Proposition 1

Consider a solution $\tilde{\phi }$ of the linearised field equations and assume that the vector field *X* is independent of the fields considered. The Hamiltonian current

2.29 $$\begin{aligned} {\,\,\,\widetilde{\mathscr {H}}}^\mu [X] {:}{=} \tilde{{\pi }}^\mu {}_ A{\mathcal {L}}_X \tilde{\phi }^A - X^\mu {\,\,\,\widetilde{\mathscr {L}}} \end{aligned}$$

of the linearised theory can be rewritten as

2.30 $$\begin{aligned} {\,\,\,\widetilde{\mathscr {H}}}^\mu [X] = \frac{1}{2} \omega ^\mu (\tilde{\phi }, {\mathcal {L}}_X \tilde{\phi }) + \partial _{{\sigma }} \Big ( X^{[{{\sigma }}} \tilde{{\pi }}_A{}^{\mu ]} \tilde{\phi }^A \Big ) \,. \end{aligned}$$

Here ${\,\,\,\widetilde{\mathscr {L}}}$ is the Lagrangian density for the linearised equations, with $\tilde{{\pi }}^A{}_\mu = \partial {\,\,\,\widetilde{\mathscr {L}}}/ \partial (\partial _\mu \tilde{\phi }^A)$, and $\omega ^\mu$ is the presymplectic current (2.19).

In view of the above, the Hamiltonian ${\,\,\,\widetilde{\mathscr {H}}}(\mathscr {S},X)$ for the linearised theory associated with a hypersurface $\mathscr {S}$ reads

2.31 $$\begin{aligned} {\,\,\,\widetilde{\mathscr {H}}}[\mathscr {S}, X]&{:}{=} \int _\mathscr {S}\big ( \tilde{{\pi }}^A{}_\mu {\mathcal {L}}_X \tilde{\phi }^A - X^\mu {\,\,\,\widetilde{\mathscr {L}}}\big ) dS_\mu \nonumber \\&= \frac{1}{2} \left( \int _\mathscr {S}\omega ^\mu (\tilde{\phi }, {\mathcal {L}}_X \tilde{\phi }) \, dS_\mu \right. \nonumber \\ {}&\quad \left. {-} \int _{\partial \mathscr {S}} X^{[{{\sigma }}} \tilde{{\pi }}_A{}^{\mu ]} \tilde{\phi }^A dS_{{{\sigma }}\mu } \right) \,. \end{aligned}$$

When *X* is a time-translation, one often identifies the numerical value of (2.292.31) with the energy of the field contained in $\mathscr {S}$. We will use this terminology, momentarily ignoring all the delicate issues associated with the boundary conditions satisfied by the fields, to which we will return in due course.

#### Energy flux

We wish to derive a formula for the flux of energy across $\partial \mathscr {S}$. For this, define

2.32 $$\begin{aligned} \phi ( \tau )= \varPhi _\tau [Y]\big (\phi \big ) \,, \end{aligned}$$

where we use the symbol $\varPhi _\tau [Y]$ to denote both the flow of a vector field *Y* and its action on our field. We consider a family of fields obtaining by flowing along *Y*, and the resulting variational identity. We will require that *X* commutes with *Y*, that the background is invariant under the flow of *Y*, and that all the $\phi (\tau )$’s are solutions of the field equations. Equation (2.14) with $\lambda$ replaced by $\tau$ reads

2.33 $$\begin{aligned} \frac{d \mathscr {H}^\mu [X]}{d\tau }= & {} {\mathcal {L}}_X \phi ^A \frac{d {\pi }_A{}^\mu }{d\tau } - {\mathcal {L}}_X {\pi }_A{}^\mu \frac{d \phi ^A}{d\tau }\nonumber \\&+ 2 \partial _{{\sigma }} \left( X^{[{{\sigma }}} {\pi }_A{}^{\mu ]} \frac{d \phi ^A}{d\tau } \right) \nonumber \\= & {} {\mathcal {L}}_X \phi ^A {\mathcal {L}}_Y {\pi }_A{}^\mu - {\mathcal {L}}_X {\pi }_A{}^\mu {\mathcal {L}}_Y \phi ^A\nonumber \\&+ 2 \partial _{{\sigma }} \left( X^{[{{\sigma }}} {\pi }_A{}^{\mu ]} {\mathcal {L}}_Y \phi ^A \right) \,. \end{aligned}$$

Taking $Y=X$, Eq. (2.33) becomes

2.34 $$\begin{aligned} \frac{d \mathscr {H}^\mu [X]}{d\tau }= & {} 2 \partial _{{\sigma }} \left( X^{[{{\sigma }}} {\pi }_A{}^{\mu ]} {\mathcal {L}}_X\phi ^A \right) \,. \end{aligned}$$

This is the field-theoretical analogue of the statement that a Hamiltonian in mechanics is conserved along its flow, except that here one needs to take into account the boundary term. Indeed, given a hypersurface $\mathscr {S}$ set

$$\begin{aligned} \mathscr {S}_\tau {:}{=} \varPhi _\tau [X](\mathscr {S}) \end{aligned}$$

and define

2.35 $$\begin{aligned} \mathscr {H}(\mathscr {S}_\tau , X) {:}{=} \int _{\mathscr {S}_\tau } \mathscr {H}^\mu dS_\mu \,. \end{aligned}$$

It follows from (2.34) that

2.36 $$\begin{aligned} \frac{d\mathscr {H}(\mathscr {S}_\tau , X)}{d\tau } = {-} \int _{\partial \mathscr {S}_\tau } X^{[{{\sigma }}} {\pi }_A{}^{\mu ]} {\mathcal {L}}_X\phi ^A dS_{{{\sigma }} \mu } \,, \end{aligned}$$

so that the integrand of (2.36) represents the flux of energy through $\partial \mathscr {S}$ when $\mathscr {S}$ is dragged along the flow of *X*.

#### The divergence of the Noether current

An important consequence of the hypotheses H1.-H2., p. 5, is the identity

2.37 $$\begin{aligned} \partial _\mu \mathscr {H}^\mu [X]= \mathscr {E}_A {\mathcal {L}}_X \phi ^A \,. \end{aligned}$$

Note that the right-hand side is zero if a) either the field equations $\mathscr {E}^A=0$ hold, or b) the solution is stationary in the sense that ${\mathcal {L}}_X \phi = 0$.

The identity is easiest to establish by going to coordinates in which ${\mathcal {L}}_X={\partial _0}$, so that

2.38 $$\begin{aligned} \partial _\mu \mathscr {H}^\mu [X]= & {} \partial _\mu ({\pi }_A{}^\mu {\partial _0} \phi ^A - \delta ^\mu _{0} \mathscr {L}) \nonumber \\= & {} ( \partial _\mu {\pi }_A{}^\mu ) {\partial _0} \phi ^A + {\pi }_A{}^\mu \partial _\mu {\partial _0} \phi ^A - {\partial _0}\mathscr {L}\nonumber \\= & {} ( {\pi }_A + \mathscr {E}_A) \partial _{0} \phi ^A + {\pi }_A{}^\mu \partial _\mu \partial _{0} \phi ^A\nonumber \\&-\frac{ \partial \mathscr {L}}{\partial \phi ^A} \partial _{0} \phi ^A -\frac{ \partial \mathscr {L}}{\partial \phi ^A_\mu } \partial _{0} \partial _\mu \phi ^A \nonumber \\= & {} \mathscr {E}_A \partial _{0} \phi ^A \,, \end{aligned}$$

which is the same as (2.37).

Formula (2.37) provides an alternative derivation of (2.36), as follows: Let *X* be a vector field everywhere transversal to a hypersurface $\mathscr {S}$ with boundary $\partial \mathscr {S}$. Let, as before, $\mathscr {S}_\tau$ be obtained by flowing $\mathscr {S}$ with the vector field *X* for a time $\tau$. Let us denote by $T_\tau$ the hypersurface obtained by flowing the boundary of $\mathscr {S}$ from time zero to time $\tau$:

$$\begin{aligned} T_\tau =\cup _{s\in [0,\tau ]} \phi _s[X](\partial \mathscr {S}) \,. \end{aligned}$$

Supposing that the right-hand side of (2.37) vanishes, and applying Stokes’ theorem on the set bounded by $\mathscr {S}$, $\mathscr {S}_\tau$ and $T_\tau$ we obtain

2.39 $$\begin{aligned} H[\mathscr {S}_\tau ,X]= H[\mathscr {S}_{0},X] + \int _{T_\tau }\mathscr {H}^\mu dS_\mu \,. \end{aligned}$$

Differentiating with respect to $\tau$ one obtains

2.40 $$\begin{aligned} \frac{dH[\mathscr {S}_\tau ,X]}{d\tau }= {-} \int _{\partial \mathscr {S}} X^{[\nu } \mathscr {H}^{\mu ]} dS_{\nu \mu } \,. \end{aligned}$$

This coincides with (2.36), as the term $X^\mu \mathscr {L}$ in the definition of $\mathscr {H}^\mu$ drops out from the integral after antisymmetrisation.

#### The energy flux revisited

In the case of theory of fields linearised around a solution, there exists yet another way of computing the flux of energy across $\partial \mathscr {S}$, as follows:^1^

Since the divergence of $\omega ^\mu$ vanishes for solutions of field equations, the calculation leading to (2.40) gives

2.41 $$\begin{aligned} \frac{d}{d\tau } \int _\mathscr {S}\omega ^\mu (\tilde{\phi }, {\mathcal {L}}_X \tilde{\phi }) \, dS_\mu = - \int _{\partial \mathscr {S}} X^{[{{\sigma }}} \omega {}^{\mu ]}(\tilde{\phi }, {\mathcal {L}}_X \tilde{\phi })\, dS_{{{\sigma }}\mu }.\nonumber \\ \end{aligned}$$

Calculating the $\tau$-derivative of ${\,\,\,\widetilde{\mathscr {H}}}[\mathscr {S}, X]$ as given by (2.292.31) we obtain

2.42 $$\begin{aligned}&\frac{d {\,\,\,\widetilde{\mathscr {H}}}[\mathscr {S}_\tau , X]}{d\tau }\nonumber \\&\quad = \frac{1}{2} \frac{d}{d\tau } \int _\mathscr {S}\omega ^\mu (\tilde{\phi }, {\mathcal {L}}_X \tilde{\phi }) \, dS_\mu \nonumber \\&\quad - \frac{1}{2}\int _{\partial \mathscr {S}} {\mathcal {L}}_X \left( X^{[{{\sigma }}} \tilde{{\pi }}_A{}^{\mu ]} \tilde{\phi }^A\right) dS_{{{\sigma }}\mu } \nonumber \\&\quad = -\frac{1}{2}\int _{\partial \mathscr {S}} X^{[{{\sigma }}} \omega {}^{\mu ]}(\tilde{\phi }, {\mathcal {L}}_X \tilde{\phi })\, dS_{{{\sigma }}\mu } \nonumber \\&\qquad - \frac{1}{2}\int _{\partial \mathscr {S}}\left( X^{[{{\sigma }}} {\mathcal {L}}_X\tilde{{\pi }}_A{}^{\mu ]} \tilde{\phi }^A + X^{[{{\sigma }}} \tilde{{\pi }}_A{}^{\mu ]} {\mathcal {L}}_X \tilde{\phi }^A \right) dS_{{{\sigma }}\mu } \,.\nonumber \\ \end{aligned}$$

Inserting the definition $\omega ^{\mu }(\tilde{\phi }, {\mathcal {L}}_X \tilde{\phi }){:}{=} {\mathcal {L}}_X \tilde{\phi }^{A}\tilde{{\pi }}_A{}^{\mu }- \tilde{\phi }^{A} {\mathcal {L}}_X \tilde{{\pi }}_{A}{}^{\mu }$, one term cancels out and another gets doubled, resulting in the energy flux equal to

2.43 $$\begin{aligned} \frac{d{\,\,\,\widetilde{\mathscr {H}}}(\mathscr {S}_\tau , X)}{d\tau } = - \int _{\partial \mathscr {S}_\tau } X^{[{{\sigma }}} \tilde{{\pi }}_A{}^{\mu ]} {\mathcal {L}}_X\tilde{\phi }^A dS_{{{\sigma }} \mu } \,, \end{aligned}$$

recovering again (2.36).

### Scalar fields on de Sitter spacetime

We apply the formalism to a linear scalar field in Minkowski spacetime and in de Sitter spacetime. In our signature the Lagrangian reads

2.44 $$\begin{aligned} \mathscr {L}=- \frac{1}{2} \sqrt{|-\det g|} \big ( g^{\mu \nu }\partial _\mu \phi \, \partial _\nu \phi {+m^2}\phi ^2 \big ) \,, \end{aligned}$$

for a constant *m*. The theory coincides with its linearisation and we will therefore not make a distinction between the fields $\varphi$ and $\tilde{\varphi }$. The canonical energy-momentum current $\mathscr {H}^\mu$ equals

2.45 $$\begin{aligned} \mathscr {H}^\mu [X]= & {} - \sqrt{|-\det g|}\nonumber \\&\times \left( \nabla ^\mu \phi \,{\mathcal {L}}_X \phi - \frac{1}{2} \big (\nabla ^\alpha \phi \nabla _\alpha \phi {+m^2}\phi ^2 \big ) X^\mu \right) .\nonumber \\ \end{aligned}$$

We consider simultaneously the Minkowski space-time and the de Sitter space-time in Bondi coordinates, in which the metric takes the form

2.46 $$\begin{aligned} {{g}}\equiv {{g}}_{\alpha \beta } dx^\alpha dx^\beta = \epsilon {N}^2 du^2-2du \, dr + r^2 \underbrace{(d\theta ^2+\sin ^2 \theta d\phi ^2)}_{{=}{:}{\mathring{{\gamma }}}} , \end{aligned}$$

with $$\varLambda \ge 0$$,

$$\begin{aligned} {N}{:}{=} \sqrt{| (1-\alpha ^2 r^2)|} \,, \quad \alpha \in \left\{ 0,\sqrt{\frac{\varLambda }{3}} \right\} \,, \quad \epsilon \in \{\pm 1\} \,, \end{aligned}$$

with $\epsilon$ equal to one if $1-\alpha ^2 r^2<0$, and minus one otherwise. Hence

$$\begin{aligned} g^{\alpha \beta }\partial _\alpha \partial _\beta = -2 \partial _u\partial _r - \epsilon {N}^2 (\partial _r)^2 + r^{-2}{\mathring{{\gamma }}}^{AB}\partial _A\partial _B \,, \end{aligned}$$

and

2.47 $$\begin{aligned} \nabla \phi = - \partial _r \phi \,\partial _u - (\partial _u \phi + \epsilon {N}^2 \partial _r \phi )\partial _r + r^{-2}{\mathring{{\gamma }}}^{AB}\partial _A \phi \, \partial _B \,. \end{aligned}$$

(Starting from a more usual form of the de Sitter metric,

2.48 $$\begin{aligned} g = -(1-\alpha ^2 r^2)dt^2 + \frac{dr^2}{1-\alpha ^2 r^2} + r^2 (d\theta ^2+\sin ^2 \theta d\phi ^2) \,, \end{aligned}$$

the form (2.46) can be obtained, for $\alpha r >1$, by introducing a coordinate *u* through the formula

2.49 $$\begin{aligned} du : = dt - \frac{dr}{1-\alpha ^2 r^2} \equiv d \left( t + \frac{1}{2 \alpha } \ln \left( \frac{\alpha r -1}{\alpha r + 1} \right) \right) \,; \end{aligned}$$

cf., e.g., [27].)

We denote by $\mathscr {C}_{u}$ the light cone of constant *u*, and by $\mathscr {C}_{u,R}$ its truncation in which the Bondi coordinate *r* ranges from zero to *R*. We wish to calculate the canonical energy associated with the Killing vector field $X=\partial _u$ and contained in $\mathscr {C}_{u,R}$. Letting

2.50 $$\begin{aligned} d\mu _{\mathscr {C}}= & {} \sqrt{\det g_{AB}} \; dr\wedge dx^2\wedge dx^3 \quad \text{ and } \nonumber \\ d\mu _{{\mathring{{\gamma }}}}= & {} \sqrt{\det {\mathring{{\gamma }}}_{AB}} \; dx^2\wedge dx^3 \end{aligned}$$

we find

2.51 $$\begin{aligned}&E_c[\phi ,\mathscr {C}_{u,R}] {:}{=} \int _{\mathscr {C}_{u,R}} \mathscr {H}^\mu [\partial _u] dS_\mu = \int _{\mathscr {C}_{u,R}} \mathscr {H}^u [\partial _u] dS_u \nonumber \\&\quad = \frac{1}{2} \int _{\mathscr {C}_{u,R}} ( \nabla ^\alpha \phi \nabla _\alpha \phi - 2\nabla ^u\phi \, \partial _u \phi +m^2 \phi ^2 ) d\mu _{\mathscr {C}} \nonumber \\&\quad = \frac{1}{2} \int _{\mathscr {C}_{u,R}} \big ( g^{AB}\partial _A \phi \, \partial _B \phi -\epsilon {N}^2 ( \partial _r \phi )^2 +m^2 \phi ^2 \big ) r^2 \, dr \, d\mu _{{\mathring{{\gamma }}}} \,. \nonumber \\ \end{aligned}$$

We see that $(\partial _r\phi )^2$ gives a positive contribution to the energy integral in the region where $g_{uu} = \epsilon {N}^2$ is negative, and a negative contribution otherwise.

The presymplectic current is defined as

2.52 $$\begin{aligned} \omega ^\mu (\delta _1 \phi , \delta _2 \phi ) \!=\! (\delta _{1} \phi \nabla ^{\mu } \delta _{2} \phi \!-\!\delta _{2} \phi \nabla ^{\mu } \delta _{1} \phi ) \sqrt{|\det g |}, \end{aligned}$$

with obviously vanishing divergence on solutions of field equations:

$$\begin{aligned} \nabla _\mu \omega ^\mu \equiv \partial _\mu \omega ^\mu = 0 \,. \end{aligned}$$

The *u*-component of the presymplectic current on $\mathscr {C}_u$ reads

2.53 $$\begin{aligned} \omega ^u(\delta _1\phi , \delta _2\phi ) = \big ( \delta _2 \phi \, \partial _r \delta _1 \phi - \delta _1 \phi \, \partial _r \delta _2 \phi \big ) \sqrt{|\det g|} \,. \end{aligned}$$

When $X=\partial _u$, this results in the following volume integrand in (2.30)

2.54 $$\begin{aligned} \frac{1}{2 } \omega ^u( \phi , \partial _u\phi ) = \frac{1}{2} \big ( \partial _u \phi \, \partial _r \phi - \phi \, \partial _r \partial _u \phi \big ) \sqrt{|\det g|} \,, \end{aligned}$$

while the boundary integrand equals

2.55 $$\begin{aligned} \partial _{{\sigma }} \Big ( X^{[{{\sigma }}} \tilde{{\pi }}^{\mu ]} \phi \Big ) = \frac{1}{2} \partial _{i} \Big ( \phi \nabla ^i \phi \sqrt{|\det g|} \Big ) \,. \end{aligned}$$

Equation (2.30) leads to the following alternative form of (2.51):

2.56 $$\begin{aligned}&E_c[\phi ,\mathscr {C}_{u,R}] \nonumber \\&\quad = \int _{\mathscr {C}_{u,R}} \left( \frac{1}{2} \omega ^\mu (\tilde{\phi }, {\mathcal {L}}_X \tilde{\phi }) + \partial _{{\sigma }} \big ( X^{[{{\sigma }}} \pi ^{\mu ]} \tilde{\phi }\big ) \right) dS_\mu \nonumber \\&\quad = \frac{1}{2} \int _{\mathscr {C}_{u,R}} \Big ( \partial _u \phi \, \partial _r \phi - \phi \, \partial _r \partial _u \phi \Big ) d\mu _{\mathscr {C}} \nonumber \\&\qquad + \frac{1}{2} \int _{S_{u,R}} \phi \nabla ^i \phi \sqrt{|\det g|} \partial _i\rfloor ( dx^1\wedge dx^2\wedge dx^3) \end{aligned}$$

2.57 $$\begin{aligned}&\quad = \frac{1}{2} \int _{\mathscr {C}_{u,R}} \Big ( \partial _u \phi \, \partial _r \phi - \phi \, \partial _r \partial _u \phi \Big ) d\mu _{\mathscr {C}} \nonumber \\&\qquad - \frac{1}{2} \int _{S_{u,R}} \phi (\partial _u \phi + \epsilon N^2 \partial _r \phi ) \sqrt{|\det g|} \, dx^2\wedge dx^3. \,\,\, \nonumber \\ \end{aligned}$$

A careful reader might justly worry about the convergence of the integrals, since a light cone is not a smooth manifold. The fact that all integrals in this section are well behaved for solutions of the wave equation which are smooth in a neighborhood of the light cone can be verified using [14, Proposition 2.1].

As a check of equality of (2.56) and (2.51), we note that the field equation for $\phi$ reads

2.58 $$\begin{aligned}&m^2 \sqrt{|\det g|} \phi \nonumber \\&\quad = \sqrt{|\det g|}\, \Box \phi = \partial _\mu ( \sqrt{|\det g|} \nabla ^\mu \phi ) \nonumber \\&\quad = - \sqrt{|\det g|} \partial _u \partial _r \phi + \partial _i ( \sqrt{|\det g|} \nabla ^i \phi ) \end{aligned}$$

2.59 $$\begin{aligned}&\quad = - r^2\sqrt{\det {\mathring{{\gamma }}}} \partial _u \partial _r \phi - \partial _r \big ( r^2\sqrt{\det {\mathring{{\gamma }}}} (\partial _u \phi + \epsilon {N}^2 \partial _r \phi ) \big ) \nonumber \\&\qquad + \partial _A (\sqrt{\det {\mathring{{\gamma }}}} {\mathring{{\gamma }}}^{AB}\partial _B \phi ) \nonumber \\&\quad = - 2r \sqrt{\det {\mathring{{\gamma }}}}\partial _r (r \partial _u \phi ) - \partial _r \big ( r^2\sqrt{\det {\mathring{{\gamma }}}} \epsilon {N}^2 \partial _r \phi \big ) \nonumber \\&\qquad + \partial _A (\sqrt{\det {\mathring{{\gamma }}}} {\mathring{{\gamma }}}^{AB}\partial _B \phi ) \,. \end{aligned}$$

Equation (2.58) together with the divergence theorem can be used to replace the boundary term in (2.56) by a volume integral, indeed recovering (2.51).

The mass-flux formula (2.36) becomes

2.60 $$\begin{aligned}&\frac{dE_c[\phi ,\mathscr {C}_{u,R}]}{d u } \nonumber \\&\quad \equiv \frac{d\mathscr {H}(\mathscr {C}_{u,R}, \partial _u)}{d u } = - \int _{\partial \mathscr {S}_\tau } X^{[{{\sigma }}} \pi ^{\mu ]} \partial _u\phi \, dS_{{{\sigma }} \mu } \nonumber \\&\quad = \int _{S_{u,R}} \nabla ^{r} \phi \, \partial _u\phi \, r^2 \, d\mu _{{\mathring{{\gamma }}}} \nonumber \\&\quad = - \int _{S_{u,R}} \big ( (\partial _u\phi )^2 + \epsilon N^2 \partial _r \phi \, \partial _u \phi \big ) \, r^2 \, d\mu _{{\mathring{{\gamma }}}} \,. \end{aligned}$$

Let us momentarily assume that $\varLambda =0=m$. As is well known, there exists a large class of solutions of the wave equation on Minkowski spacetime with full asymptotic expansions, for large *r*,

2.61 $$\begin{aligned} \phi (u,r, x^A) = \frac{\overset{{ (-1)}}{\phi }(u,x^A)}{r} + \frac{\overset{{ (-2)}}{\phi }(u,x^A)}{r^2} + \frac{\overset{{ (-3)}}{\phi }(u,x^A)}{r^3} +\cdots \end{aligned}$$

After passing to the limit $R\rightarrow \infty$, for such solutions (2.60) becomes the usual energy-loss formula for the scalar field:

2.62 $$\begin{aligned} \frac{dE_c[\phi ,\mathscr {C}_{u }]}{d u }= & {} - \int _{S^2} (\partial _u\overset{{ (-1)}}{\phi })^2\, d\mu _{{\mathring{{\gamma }}}} \,. \end{aligned}$$

We now turn our attention to the de Sitter case. We first consider a massive scalar field, with the mass chosen so that the equation is conformally covariant,

2.63 $$\begin{aligned} \Box _g \phi - \underbrace{\frac{(d-2)R(g)}{4 (d-1)}}_{{=}{:}m^2} \phi = 0 \,, \end{aligned}$$

where *d* is the dimension of spacetime and *R*(*g*) is the scalar curvature of *g*. After a conformal transformation $g\mapsto \varOmega ^2 g$ the field $\varOmega ^{d/2-1} \phi$ satisfies again (2.63), with *g* there replaced by $\varOmega ^2 g$. This implies that solutions of (2.63) with smooth initial data on a Cauchy surface in de Sitter spacetime behave asymptotically for large *r*, in spacetime dimension four, again as in (2.61). We return to this in Sect. 2.2.1 below.

For such solutions (2.57) becomes

2.64 $$\begin{aligned} E_c[\phi ,\mathscr {C}_{u,R}]= & {} \frac{1}{2} \int _{\mathscr {C}_{u,R}} \Big ( \partial _u \phi \, \partial _r \phi - \phi \, \partial _r \partial _u \phi \Big ) d\mu _{\mathscr {C}} \nonumber \\&- \frac{1}{2} \int _{S^2} \overset{{ (-1)}}{\phi }\big ( \partial _u\overset{{ (-1)}}{\phi }- \alpha ^2 R \overset{{ (-1)}}{\phi }\big ) \, d\mu _{{\mathring{{\gamma }}}} + o(1) \,, \nonumber \\ \end{aligned}$$

where the volume integral converges when passing with the radius *R* to infinity, but the boundary integral diverges linearly in *R* in general. Indeed, given $\phi |_{\mathscr {C}_u}$ one can integrate (2.59) to

2.65 $$\begin{aligned}&\partial _u \phi \nonumber \\&\quad = \frac{1}{2r}\int _{0}^{r}\frac{1}{\rho } \big ( - \partial _r \big ( r^2 \epsilon {N}^2 \partial _r \phi \big ) - {m^2} r^2 \phi + {\varDelta _{{{\mathring{{\gamma }}}}}} \phi \big )\big |_{r=\rho } d\rho \nonumber \\&\quad = \frac{ \partial _u \overset{{ (-1)}}{\phi }}{r}+ \frac{ 2 \alpha ^2 \overset{{ (-3)}}{\phi }- \varDelta _{{{\mathring{{\gamma }}}}} \overset{{ (-1)}}{\phi }}{2 r^2} + \cdots \,, \end{aligned}$$

where $\varDelta _{{{\mathring{{\gamma }}}}}$ is the Laplace operator associated with the metric ${{\mathring{{\gamma }}}}$, and where we have used

2.66 $$\begin{aligned} m^2 = 2 \alpha ^2 \end{aligned}$$

in spacetime dimension four. Here

2.67 $$\begin{aligned} \partial _u \overset{{ (-1)}}{\phi }= & {} \int _{0}^{\infty }\frac{1}{2\rho } \big ( - \partial _r \big ( r^2 \epsilon {N}^2 \partial _r \phi \big ) - 2 \alpha ^2 r^2 \phi + {\varDelta _{{{\mathring{{\gamma }}}}}} \phi \big )\big |_{r=\rho } d\rho ,\nonumber \\ \end{aligned}$$

which shows that $\overset{{ (-1)}}{\phi }$ will not be zero at later times in general even if it is initially.

As for (2.60), we find

2.68 $$\begin{aligned}&\frac{dE_c[\phi ,\mathscr {C}_{u,R}]}{d u } \nonumber \\&\quad = - \int _{S^2} \bigg ( (\partial _u\overset{{ (-1)}}{\phi })^2 - \alpha ^2 R \overset{{ (-1)}}{\phi }\partial _u\overset{{ (-1)}}{\phi }\bigg ) \, d\mu _{{\mathring{{\gamma }}}} + o(1).\nonumber \\ \end{aligned}$$

This diverges again when *R* tends to infinity. However, we see that the divergent term in $E_c$ has a dynamics of its own, so that a *renormalised energy* can be obtained by subtracting the divergent term in $E_c$ and passing with *R* to infinity,

2.69 $$\begin{aligned} E [\phi ,\mathscr {C}_{u }]&{:}{=}\frac{1}{2} \int _{\mathscr {C}_{u }} \Big ( \partial _u \phi \, \partial _r \phi - \phi \, \partial _r \partial _u \phi \Big ) d\mu _{\mathscr {C}}\nonumber \\&- \frac{1}{2} \int _{S^2} \overset{{ (-1)}}{\phi }\partial _u\overset{{ (-1)}}{\phi }\, d\mu _{{\mathring{{\gamma }}}} \,. \end{aligned}$$

The renormalised energy satisfies again an energy-loss formula identical to (2.62), formally coinciding with that in Minkowski spacetime.

A similar behaviour is observed for the massless scalar field. It follows from [55] (cf. Sect. 2.2.1) that scalar fields evolving out of smooth initial data on a Cauchy surface have an asymptotic expansion of the form

2.70 $$\begin{aligned} \phi (u,r, x^A) = \overset{{ (0)}}{\phi }(u,r, x^A) + \frac{\overset{{ (-1)}}{\phi }(u,x^A)}{r} + \frac{\overset{{ (-2)}}{\phi }(u,x^A)}{r^2} \ldots \,, \end{aligned}$$

compare (2.92).

It turns out that the volume part of $E_c$ given by (2.57) does not converge anymore as *R* tends to infinity under (2.70). Indeed, using (2.92)–(2.93) one finds

2.71 $$\begin{aligned}&\frac{1}{2} \int _{\mathscr {C}_{u,R}} \Big ( \partial _u \phi \, \partial _r \phi - \phi \, \partial _r \partial _u \phi \Big ) d\mu _{\mathscr {C}} \nonumber \\&\quad = -\frac{1}{2} \int _{S^2}\bigg ( \Big ( \alpha ^{2} (\overset{{ (-1)}}{\phi })^{2}-\overset{{ (0)}}{\phi }\partial _u \overset{{ (-1)}}{\phi }\Big ) R \nonumber \\&\qquad +\mathring{D}^{A}\Big ( \overset{{ (-1)}}{\phi }\mathring{D}_{A}\overset{{ (0)}}{\phi }- \overset{{ (0)}}{\phi }\mathring{D}_{A} \overset{{ (-1)}}{\phi }\Big ) \ln R \bigg ) \, d\mu _{{\mathring{{\gamma }}}} +O(1) \, , \nonumber \\ \end{aligned}$$

where $\mathring{D}$ is the covariant derivative associated with the metric ${{\mathring{{\gamma }}}}$ and where *O*(1) denotes terms which have a finite limit as *R* tends to infinity. Note that the logarithmic term integrates-out to zero over $S^2$. This leads us to define the finite part, say $E_{{V}}$, of the volume integral as

2.72 $$\begin{aligned} E_{{V}}&{:}{=}\lim _{R\rightarrow \infty } \left( \frac{1}{2} \int _{\mathscr {C}_{u,R}} \Big ( \partial _u \phi \, \partial _r \phi - \phi \, \partial _r \partial _u \phi \Big ) d\mu _{\mathscr {C}} \right. \nonumber \\&\left. + \frac{R}{2} \int _{S^2} \big ( \alpha ^{2} (\overset{{ (-1)}}{\phi })^{2}-\overset{{ (0)}}{\phi }\partial _u \overset{{ (-1)}}{\phi }\big ) \, d\mu _{{\mathring{{\gamma }}}} \right) . \end{aligned}$$

One now finds

2.73 $$\begin{aligned}&E_c [\phi ,\mathscr {C}_{u,R }] \nonumber \\&\quad {:}{=} E_{{V}} -\frac{1}{2} \int _{S^2}\Bigg ( \Big ( \alpha ^{2} (\overset{{ (-1)}}{\phi })^{2}-\overset{{ (0)}}{\mathring{D}}_{A} \phi \mathring{D}^{A} \overset{{ (0)}}{\phi }\Big ) R \nonumber \\&\qquad +\overset{{ (0)}}{\phi }\overset{{ (-1)}}{\phi }+\overset{{ (-1)}}{\phi }\partial _{u} \overset{{ (-1)}}{\phi }-3 \alpha ^{2} \overset{{ (0)}}{\phi }\overset{{ (-3)}}{\phi }\nonumber \\&\qquad \underbrace{ +\overset{{ (-1)}}{\phi }{\varDelta _{{{\mathring{{\gamma }}}}}} \overset{{ (0)}}{\phi }-\frac{1}{2} \overset{{ (0)}}{\phi }{\varDelta _{{{\mathring{{\gamma }}}}}} \overset{{ (-1)}}{\phi }}_{=\frac{1}{2}\overset{{ (-1)}}{\phi }{\varDelta _{{{\mathring{{\gamma }}}}}} \overset{{ (0)}}{\phi }\ \hbox {after integration by parts}} \Bigg ) \, d\mu _{{\mathring{{\gamma }}}} +o(1) \, , \end{aligned}$$

which continues to diverge as *R* tends to infinity in general. The associated energy flux formula reads

2.74 $$\begin{aligned} \frac{dE_c[\phi ,\mathscr {C}_{u,R}]}{d u }= & {} - \int _{S^2} \bigg ( R \big ( {\varDelta _{{{\mathring{{\gamma }}}}}} \overset{{ (0)}}{\phi }+ \partial _{u} \overset{{ (-1)}}{\phi }\big ) \alpha ^{2} \overset{{ (-1)}}{\phi }\nonumber \\&+\Big ( \overset{{ (-1)}}{\phi }-3\alpha ^{2} \overset{{ (-3)}}{\phi }- \frac{1}{2} {\varDelta _{{{\mathring{{\gamma }}}}}} \overset{{ (-1)}}{\phi }\Big ) \alpha ^{2} \overset{{ (-1)}}{\phi }\nonumber \\&+(\partial _{u} \overset{{ (-1)}}{\phi })^2 + {\varDelta _{{{\mathring{{\gamma }}}}}} \overset{{ (0)}}{\phi }\partial _{u} \overset{{ (-1)}}{\phi }\bigg ) \, d\mu _{{\mathring{{\gamma }}}} + o(1). \nonumber \\ \end{aligned}$$

Using $\partial _u \overset{{ (0)}}{\phi }= \alpha ^2 \overset{{ (-1)}}{\phi }$ (cf. (2.93)), one finds (unsurprisingly) that the divergent term has a dynamics of its own, so that the finite renormalised energy, defined as

2.75 $$\begin{aligned} E [\phi ,\mathscr {C}_{u }]:= & {} E_{{V}}-\frac{1}{2} \int _{S^2}\bigg ( \overset{{ (0)}}{\phi }\overset{{ (-1)}}{\phi }+\overset{{ (-1)}}{\phi }\partial _{u} \overset{{ (-1)}}{\phi }-3 \alpha ^{2} \overset{{ (0)}}{\phi }\overset{{ (-3)}}{\phi }\nonumber \\&+\frac{1}{2}\overset{{ (-1)}}{\phi }{\varDelta _{{{\mathring{{\gamma }}}}}} \overset{{ (0)}}{\phi }\bigg ) \, d\mu _{{\mathring{{\gamma }}}} \end{aligned}$$

has a finite and well-defined flux:

2.76 $$\begin{aligned} \frac{dE [\phi ,\mathscr {C}_{u }]}{d u }= & {} - \int _{S^2} \bigg ( \Big ( \overset{{ (-1)}}{\phi }-3\alpha ^{2} \overset{{ (-3)}}{\phi }- \frac{1}{2} {\varDelta _{{{\mathring{{\gamma }}}}}} \overset{{ (-1)}}{\phi }\Big ) \alpha ^{2} \overset{{ (-1)}}{\phi }\nonumber \\&+(\partial _{u} \overset{{ (-1)}}{\phi })^2 + {\varDelta _{{{\mathring{{\gamma }}}}}} \overset{{ (0)}}{\phi }\partial _{u} \overset{{ (-1)}}{\phi }\bigg ) \, d\mu _{{\mathring{{\gamma }}}}. \end{aligned}$$

#### Asymptotics of scalar fields on de Sitter spacetime

In order to understand the behaviour for large *r*, in Bondi coordinates, of solutions of the massive or massless wave equation,

2.77 $$\begin{aligned} \Box \phi = m^2 \phi \,, \end{aligned}$$

on de Sitter spacetime it is most convenient to work using a foliation of (part of) de Sitter spacetime by flat submanifolds, so that the metric takes the form

2.78 $$\begin{aligned} g= -d\tau ^2 + e^{2 \sqrt{\frac{\varLambda }{3}}\tau } (dx^2 + dy^2 +dz^2) \,. \end{aligned}$$

Let $\rho =\sqrt{x^2+ y^2 +z^2}$, where (*x*, *y*, *z*) are as in (2.78), thus

2.79 $$\begin{aligned} g= -d\tau ^2 + e^{2 \sqrt{\frac{\varLambda }{3}}\tau } \big ( d\rho ^2 + \rho ^2(d\theta ^2 +\sin ^2\theta d\varphi ^2) \big ) \,. \end{aligned}$$

Set

2.80 $$\begin{aligned} T{:}{=} \alpha ^{-1} e^{-\alpha \tau } \,, \quad \alpha = \sqrt{\frac{\varLambda }{3}} \,. \end{aligned}$$

We start our discussion with the case $m=0$. According to [55], smooth solutions of the massless scalar wave equation on de Sitter spacetime extend through the conformal boundary $\{T=0\}$ as

2.81 $$\begin{aligned} \phi = f + T^3 \ln T \check{f} \,, \end{aligned}$$

where *f* and $\check{f}$ are smooth functions of (*T*, *x*, *y*, *z*). (By matching coefficients as below one finds in fact that $\check{f}\equiv 0$; in other words, $\phi$ extends smoothly across the conformal boundary.) The coordinate transformation

2.82 $$\begin{aligned} r= \rho e^{\alpha \tau } \,, \quad t= \tau - \frac{1}{ 2 \alpha } \ln (-1+ \rho ^2 \alpha ^2 e^{2 \alpha \tau } ) \,, \end{aligned}$$

brings (2.79) to the form

2.83 $$\begin{aligned} g = - V dt^2 + \frac{dr^2}{V} + r^2 (d\theta ^2 + \sin ^2\theta d\varphi ^2) \,, \quad V = 1 - \alpha ^2 {r^2} \,. \end{aligned}$$

In terms of the coordinate *u* of (2.49),

2.84 $$\begin{aligned} u = t + \frac{1}{2 \alpha } \ln \big (\frac{\alpha r -1}{\alpha r + 1} \big ) \,, \end{aligned}$$

one finds

2.85 $$\begin{aligned} u = \tau - \frac{1}{ \alpha } \ln ( \alpha r + 1) \quad \Longleftrightarrow \quad T= \frac{e^{-\alpha u}}{\alpha (1+\alpha r)} \,, \end{aligned}$$

as well as

2.86 $$\begin{aligned} \rho = \frac{r e^{-\alpha u}}{1+\alpha r} \,. \end{aligned}$$

We thus have the expansions as $r\rightarrow \infty$, absolutely convergent for $r > \alpha ^{-1}$,

2.87 $$\begin{aligned} \rho= & {} \frac{ e^{-\alpha u}}{ \alpha } \sum _{n=0}^\infty \left( -\frac{1}{ \alpha r}\right) ^n = \frac{ e^{-\alpha u}}{ \alpha } \left( 1 -\frac{1}{ \alpha r} + \frac{1}{ (\alpha r)^2} \,{+}\, \cdots \right) , \nonumber \\ T= & {} \frac{ e^{-\alpha u}}{ \alpha ^2 r } \sum _{n=0}^\infty \left( -\frac{1}{ \alpha r}\right) ^n \,{=}\, \frac{e^{-\alpha u}}{\alpha }\left( \frac{1}{ \alpha r} \,{-}\, \frac{1}{ (\alpha r)^2} \,{+}\, \cdots \right) . \nonumber \\ \end{aligned}$$

This shows that a Taylor-series in *T*, near $T=0$, for a function *f*,

2.88 $$\begin{aligned} f(T,\rho ,\theta ,\varphi ) = f(0,\rho ,\theta ,\varphi ) + \partial _T f(0,\rho ,\theta ,\varphi ) T + \cdots \,, \end{aligned}$$

translates into a full asymptotic expansion in 1/*r*, for large *r*:

2.89 $$\begin{aligned}&\underbrace{f|_{(T=0,\rho =\frac{e^{-\alpha u}}{\alpha },\theta ,\varphi )}}_{\overset{{ (0)}}{f} (u,\theta ,\varphi )} + \underbrace{ \frac{ e^{-\alpha u}( \partial _T f- \partial _\rho f)|_{(T=0,\rho =\frac{e^{-\alpha u}}{\alpha },\theta ,\varphi )} }{\alpha } }_{\overset{{ (-1)}}{f}(u,\theta ,\varphi )} \nonumber \\&\quad \times \frac{1 }{ r } + \cdots \,. \end{aligned}$$

Using

$$\begin{aligned} \ln (\alpha T)= & {} - \alpha u -\ln (1+\alpha r)\nonumber \\= & {} \ln (\alpha r) \left( 1 + \frac{1}{\alpha r} + \cdots \right) - \alpha u \,, \end{aligned}$$

Equation (2.89) translates to the following asymptotic expansion for solutions $\phi$ of the massless wave equation:

2.90 $$\begin{aligned} \phi (u,r, x^A)= & {} \overset{{ (0)}}{\phi }(u,x^A) + \frac{\overset{{ (-1)}}{\phi }(u,x^A)}{r} + \frac{\overset{{ (-2)}}{\phi }(u,x^A) }{r^2}\nonumber \\&+ \frac{\overset{{ (-3)}}{\phi }(u,x^A)}{r^3} + \frac{ \overset{{ (-3,1)}}{\phi }(u,x^A)\ln r}{r^3} + \cdots .\nonumber \\ \end{aligned}$$

Here some words of caution are in order. When considering the characteristic Cauchy problem for the wave equation with initial data on a light cone $\mathscr {C}(u_0)$, any function $\phi |_{\mathscr {C}(u_0)}$ can be used as initial data. In particular we can prescribe $\phi$ of the form (2.90) at $u=u_0$ with arbitrary expansion functions $\overset{{ (-k)}}{\phi }$. However, some relations will have to be satisfied by the coefficients so that $\phi$ extends as in (2.81). This can be seen by inserting the Taylor expansion (5.65) in the massless wave equation in the coordinates of (2.78) to find that solutions take the following form near $T=0$:

2.91 $$\begin{aligned} \phi (T,x^i)= & {} f(x^i) - \frac{1}{2} \varDelta _\delta f(x^i) T^2 + \frac{1}{6} \breve{f}(x^i) T^3\nonumber \\&- \frac{1}{8} \varDelta _\delta ^2 f(x^i) T^4 + \cdots \,, \end{aligned}$$

with arbitrary functions *f* and $\breve{f}$, where $\varDelta _\delta$ is the Laplacian on Euclidean $\mathbb {R}^3$, and where no logarithmic terms occur, so that the function $\check{f}$ in (2.81) is zero.

Using (2.91), the expansion (2.90) becomes

2.92 $$\begin{aligned} \phi (u,r, x^A)= & {} f\left( \frac{e^{-\alpha u}}{\alpha },x^A\right) - \frac{e^{-\alpha u}\partial _\rho f\left( \frac{e^{-\alpha u}}{\alpha },x^A\right) }{\alpha ^2r} \nonumber \\&- \frac{\varDelta _{{{\mathring{{\gamma }}}}}f\left( \frac{e^{-\alpha u}}{\alpha },x^A\right) }{2 (\alpha r)^2} + \cdots \nonumber \\= & {} \overset{{ (0)}}{\phi }(u,x^A) + \frac{ \overset{{ (-1)}}{\phi }(u, x^A) }{\alpha ^2 r } - \frac{\varDelta _{{{\mathring{{\gamma }}}}}\overset{{ (0)}}{\phi }(u,x^A) }{2 (\alpha r)^2}\nonumber \\&+ \cdots \,. \end{aligned}$$

where $\overset{{ (0)}}{\phi }(u,\cdot ) =f\big (\frac{e^{-\alpha u}}{\alpha },\cdot \big )$ and $\overset{{ (-3)}}{\phi }$ are arbitrary. Note that $\overset{{ (-2)}}{\phi }$ is determined uniquely by $\overset{{ (0)}}{\phi }$.

As a consistency check, given $\phi |_{\mathscr {C}_u}$ of the form (2.92) one can integrate (2.59) to determine $\partial _u \phi$ on $\mathscr {C}_u$:

2.93 $$\begin{aligned} \partial _u \phi= & {} \frac{1}{2r}\int _{0}^{r}\frac{1}{\rho } \big ( - \partial _r \big ( r^2 \epsilon {N}^2 \partial _r \phi \big ) + {\varDelta _{{{\mathring{{\gamma }}}}}} \phi \big )\big |_{r=\rho } d\rho \nonumber \\= & {} \alpha ^2 \overset{{ (-1)}}{\phi }+ \frac{\partial _u \overset{{ (-1)}}{\phi }}{r } - \frac{\varDelta _{{{\mathring{{\gamma }}}}} \overset{{ (-1)}}{\phi }}{2 r^2} - \frac{(\varDelta _{{{\mathring{{\gamma }}}}} +2) \overset{{ (-2)}}{\phi }}{ 4 r^3} + \cdots \,.\nonumber \\ \end{aligned}$$

where

2.94 $$\begin{aligned} \partial _u \overset{{ (-1)}}{\phi }= & {} \lim _{r\rightarrow \infty } \left( \frac{1}{2} \int _{0}^{r}\frac{1}{\rho } \big ( - \partial _r \big ( r^2 \epsilon {N}^2 \partial _r \phi \big ) + {\varDelta _{{{\mathring{{\gamma }}}}}} \phi \big )\big |_{r=\rho } d\rho \right. \nonumber \\&\left. - \alpha ^2 \overset{{ (-1)}}{\phi }r \right) \,. \end{aligned}$$

We finish by a short remark on the conformally covariant case. The corresponding wave equation in the metric (2.79) becomes the massless Minkowskian wave equation in the coordinates (*T*, *x*, *y*, *z*) for the function

2.95 $$\begin{aligned} \hat{\phi }{:}{=} T^{-1} \phi \,. \end{aligned}$$

A Taylor expansion near $T=0$ of a solution $\hat{\phi }$ gives

2.96 $$\begin{aligned} \hat{\phi }(T,\rho ,\theta ,\varphi )= & {} f(\rho ,\theta ,\varphi ) + \hat{f} (\rho ,\theta ,\varphi ) T\nonumber \\&+ \frac{1}{2} \varDelta _\delta f(\rho ,\theta ,\varphi ) T^2 + \cdots \,, \end{aligned}$$

where $\varDelta _\delta$ is the Laplace operator of the flat metric $\delta$ on $\mathbb {R}^3$, and where *f* and $\hat{f}$ are arbitrary functions. Hence

2.97 $$\begin{aligned}&\phi (u,r,\cdot ) = f(\rho ,\cdot ) T + \hat{f} (\rho ,\cdot ) T^2 + \frac{1}{2} \varDelta _\delta f (\rho ,\cdot ) T^3 + \cdots \nonumber \\&\quad = \frac{e^{-\alpha u}}{1+\alpha r} f \left( \frac{r e^{-\alpha u}}{1+\alpha r },\cdot \right) + \hat{f} \left( \frac{r e^{-\alpha u}}{1+\alpha r},\cdot \right) \left( \frac{e^{-\alpha u}}{1+\alpha r}\right) ^2 \nonumber \\&\qquad + \frac{1}{2} \varDelta _\delta f \left( \frac{r e^{-\alpha u}}{1+\alpha r},\cdot \right) \left( \frac{e^{-\alpha u}}{1+\alpha r} \right) ^3 + \cdots \nonumber \,,\\&\quad = \frac{e^{-\alpha u}}{\alpha ^2 r} f \left( \frac{ e^{-\alpha u}}{ \alpha },\cdot \right) \nonumber \\&\qquad - \frac{e^{-2\alpha u}}{ \alpha ^4 r ^2} \left( \alpha e^{ \alpha u} f \left( \frac{ e^{-\alpha u}}{ \alpha },\cdot \right) + \partial _\rho f \left( \frac{ e^{-\alpha u}}{ \alpha },\cdot \right) \right. \nonumber \\&\qquad \left. - \hat{f} \left( \frac{ e^{-\alpha u}}{ \alpha },\cdot \right) \right. + \cdots \,. \end{aligned}$$

We see that the initial data for a solution which extends smoothly through the conformal boundary at infinity will have, in Bondi coordinates, arbitrary expansion coefficients $\overset{{ (-1)}}{\phi }$ and $\overset{{ (-2)}}{\phi }$, with all the remaining expansion coefficients determined uniquely by these first two.

### Linearised gravity

We apply the results of Sect. 2.1 to vacuum general relativity with cosmological constant $\varLambda$, using the background metric approach of [11]. Thus

$$\begin{aligned} (\phi ^A) \equiv (g_{\mu \nu }) \,. \end{aligned}$$

We note the usual ambiguity related to the question, how to differentiate with respect to a symmetric tensor field. When performing variations we resolve this by allowing $g_{\mu \nu }$ not to have any symmetries, with all geometric quantities such as the Christoffel symbols, the Ricci tensor, or the volume form defined using the symmetric part $g_{(\mu \nu )}$ of $g_{\mu \nu }$. The tensor field $g_{\mu \nu }$ is assumed to be symmetric in all final formulae and in all unrelated calculations.

In [11] the Lagrangian density is obtained by removing from the Hilbert one a divergence which is made covariant by using a background metric ${b}$. After allowing for a cosmological constant, in space-time dimension *d* this leads to the Lagrangian (see [15, Section 5.1])

2.98 $$\begin{aligned} \mathscr {L}= & {} {\mathfrak {g}}^{\mu \nu } \left[ \left( {\varGamma }^{{{{\varphi }}}}_{{{{\chi }}}\mu } - {{B}}^{{{{\varphi }}}}_{{{{\chi }}}\mu } \right) \left( {\varGamma }^{{{{\chi }}}}_{{{{\varphi }}}\nu }- {{B}}^{{{{\chi }}}}_{{{{\varphi }}}\nu } \right) \right. \nonumber \\&\left. - \left( {\varGamma }^{{{{\varphi }}}}_{\mu \nu } - {{B}}^{{{{\varphi }}}}_{\mu \nu } \right) \left( {\varGamma }^{{{{\chi }}}}_{{{{\varphi }}}{{{\chi }}}} - {{B}}^{{{{\chi }}}}_{{{{\varphi }}}{{{\chi }}}} \right) + r_{\mu \nu } -\frac{2\varLambda }{d} g_{\mu \nu } \right] \,, \nonumber \\ \end{aligned}$$

where $r_{\mu \nu }$ is the Ricci tensor of ${b}$,

2.99 $$\begin{aligned} {\mathfrak {g}}^{\mu \nu } {:}{=} \frac{1}{16 \pi } \sqrt{-\det g } \ g^{\mu \nu } \,, \end{aligned}$$

and where the ${B}^{{{\varphi }}}_{{{{\psi }}} \gamma }$’s are the Christoffel symbols of the background metric ${b}$.

Consider the field

$$\begin{aligned} (\delta {\pi }_A{}^{{{\varphi }}}):= & {} \Big ({{d {\pi }_A{}^{{{\varphi }}}}\over {d\lambda }}\Big ) \equiv \Big ({{d {\pi }^{{{{\psi }}}\gamma {{{\varphi }}}}}\over {d\lambda }}\Big ) \equiv (\delta {\pi }^{{{{\psi }}}\gamma {{{\varphi }}}} )\nonumber \\:= & {} \left( {{d }\over {d\lambda }}{\frac{\partial \mathscr {L}}{\partial (\partial _{{{\varphi }}} g_{{{{\psi }}}\gamma })}}\right) \,. \end{aligned}$$

Viewing momentarily $\varGamma ^{{{\varphi }}}_{\mu \nu }$ and $g_{\mu \nu }$ as independent variables we have

2.100 $$\begin{aligned} \delta \mathscr {L}= & {} {\mathfrak {g}}^{\mu \nu } \Big [ \delta {\varGamma }^{{{{\varphi }}}}_{{{{\chi }}}\mu } \left( {\varGamma }^{{{{\chi }}}}_{{{{\varphi }}}\nu }- {{B}}^{{{{\chi }}}}_{{{{\varphi }}}\nu } \right) + \left( {\varGamma }^{{{{\varphi }}}}_{{{{\chi }}}\mu } - {{B}}^{{{{\varphi }}}}_{{{{\chi }}}\mu } \right) \delta {\varGamma }^{{{{\chi }}}}_{{{{\varphi }}}\nu }\nonumber \\&- \delta {\varGamma }^{{{{\varphi }}}}_{\mu \nu } \left( {\varGamma }^{{{{\chi }}}}_{{{{\varphi }}}{{{\chi }}}} - {{B}}^{{{{\chi }}}}_{{{{\varphi }}}{{{\chi }}}} \right) - \left( {\varGamma }^{{{{\varphi }}}}_{\mu \nu } - {{B}}^{{{{\varphi }}}}_{\mu \nu } \right) \delta {\varGamma }^{{{{\chi }}}}_{{{{\varphi }}}{{{\chi }}}} \Big ] \nonumber \\&+\frac{\partial \mathscr {L}}{\partial g_{\mu \nu }} \delta g_{\mu \nu } \nonumber \\= & {} {\mathfrak {g}}^{\mu \nu } \Big [ 2 \left( {\varGamma }^{{{{\chi }}}}_{{{{\varphi }}}\nu }- {{B}}^{{{{\chi }}}}_{{{{\varphi }}}\nu } \right) \delta ^\rho _\mu - \left( {\varGamma }^{{{{\psi }}}}_{{{{\varphi }}}{{{\psi }}}} -{{B}}^{{{{\psi }}}}_{{{{\varphi }}}{{{\psi }}}} \right) \delta ^{{{\chi }}}_\mu \delta ^\rho _\nu \nonumber \\&- \left( {\varGamma }^{{{{\chi }}}}_{\mu \nu } - {{B}}^{{{{\chi }}}}_{\mu \nu } \right) \delta ^{{\rho }}_{{{{\varphi }}} } \Big ] \delta {\varGamma }^{{{{\varphi }}}}_{{{{\chi }}}\rho } + \frac{\partial \mathscr {L}}{\partial g_{\mu \nu }} \delta g_{\mu \nu }.\nonumber \\ \end{aligned}$$

The point is that $\mathscr {L}$ depends upon the derivatives of the metric only through $\delta {\varGamma }^{{{{\varphi }}}}_{{{{\chi }}}\rho }$, so that the formula allows us to calculate ${\pi }^{\beta \gamma \alpha }$:

2.101 $$\begin{aligned} {\pi }^{\beta \gamma \alpha }= & {} {\mathfrak {g}}^{\mu \nu } \Big [ 2 \left( {\varGamma }^{{{{\chi }}}}_{{{{\varphi }}}\nu }- {{B}}^{{{{\chi }}}}_{{{{\varphi }}}\nu } \right) \delta ^\rho _\mu - \left( {\varGamma }^{{{{\psi }}}}_{{{{\varphi }}}{{{\psi }}}} -{{B}}^{{{{\psi }}}}_{{{{\varphi }}}{{{\psi }}}} \right) \nonumber \\&\delta ^{{{\chi }}}_\mu \delta ^\rho _\nu - \left( {\varGamma }^{{{{\chi }}}}_{\mu \nu } - {{B}}^{{{{\chi }}}}_{\mu \nu } \right) \delta ^{{\rho }}_{{{{\varphi }}} } \Big ] \frac{ \partial {\varGamma }^{{{{\varphi }}}}_{{{{{{\chi }}}}}\rho } }{\partial (\partial _\alpha g_{\beta \gamma })} \,. \end{aligned}$$

Denoting by ${\mathring{\nabla }}$ the covariant derivative associated with the background metric ${b}$, one has

2.102 $$\begin{aligned} \varGamma ^\sigma _{\mu \nu } - {B}^\sigma _{\mu \nu } = \frac{1}{2} g^{\sigma \rho } ( {\mathring{\nabla }}_\mu g_{\nu \rho } + {\mathring{\nabla }}_\nu g_{\mu \rho } - {\mathring{\nabla }}_\rho g_{\mu \nu } ) \,. \end{aligned}$$

A somewhat lengthy calculation allows one to rewrite (2.101) as

2.103 $$\begin{aligned} {\pi }^{\beta \gamma \alpha }= & {} {\frac{1}{16 \pi } } \sqrt{|\det g|} P^{ \alpha (\beta \gamma ) \delta (\epsilon \sigma ) } {\mathring{\nabla }}_{ \delta } g_{\epsilon \sigma } \,, \end{aligned}$$

with

2.104 $$\begin{aligned} P^{\alpha \beta \gamma \delta \epsilon \sigma }= & {} \frac{1}{2} \Big ( {{g}}^{\alpha \epsilon } {{g}}^{\delta \beta } {{g}}^{\gamma \sigma } + {{g}}^{\alpha \epsilon } {{g}}^{\sigma \beta } {{g}}^{\gamma \delta } - {{g}}^{\alpha \delta } {{g}}^{\beta \epsilon } {{g}}^{\sigma \gamma } \nonumber \\&- {{g}}^{\alpha \beta } {{g}}^{\gamma \delta } {{g}}^{\epsilon \sigma } - {{g}}^{\beta \gamma } {{g}}^{\alpha \epsilon } {{g}}^{\sigma \delta } + {{g}}^{\beta \gamma } {g}^{\alpha \delta } {{g}}^{\epsilon \sigma } \Big ).\nonumber \\ \end{aligned}$$

Note that most expressions that follow in this paper involve contractions of $P^{\alpha \beta \gamma \delta \epsilon \sigma }$ with tensors which are symmetric both in the pairs $\beta \gamma$ and $\epsilon \sigma$, and in such expressions there is no need to symmetrise $P^{\alpha \beta \gamma \delta \epsilon \sigma }$ as in (2.103), since such a symmetrisation is done automatically when the contraction is performed.

Incidentally, since $\mathscr {L}$ is quadratic in ${\mathring{\nabla }}g$, (2.104) implies

2.105 $$\begin{aligned} \mathscr {L}= & {} \frac{1}{32 \pi } \sqrt{|\det g|}\nonumber \\&\times \left( P^{ \alpha \beta \gamma \delta \epsilon \sigma } {\mathring{\nabla }}_{ \alpha } g_{\beta \gamma } {\mathring{\nabla }}_{ \delta } g_{\epsilon \sigma } + 2 g^{\mu \nu } ( r_{\mu \nu } -\frac{2\varLambda }{d} g_{\mu \nu } ) \right) ,\nonumber \\ \end{aligned}$$

which shows that it makes sense to require

2.106 $$\begin{aligned} P^{\alpha (\beta \gamma ) \delta (\epsilon \sigma )} = P^{ \delta (\epsilon \sigma ) \alpha (\beta \gamma )} \,. \end{aligned}$$

This last equation is not obvious by staring at (2.104), but can be checked by a direct calculation.

Recall that we are interested in the linearised theory. For this, it is clearly convenient to choose the background metric ${b}$ to be the metric *g* at which we are linearising. Denoting by $h_{\mu \nu }$ the linearised metric field, the Lagrangian ${\,\,\,\widetilde{\mathscr {L}}}$ for the linearised theory is thus

2.107 $$\begin{aligned} {\,\,\,\widetilde{\mathscr {L}}}= \frac{1}{32 \pi } \sqrt{|\det g|} \big ( P^{ \alpha \beta \gamma \delta \epsilon \sigma } {\mathring{\nabla }}_{ \alpha } h_{\beta \gamma } {\mathring{\nabla }}_{ \delta } h_{\epsilon \sigma } + Q(h) ) \big ) \,, \end{aligned}$$

where *Q* arises from the quadratic terms in the Taylor expansion, at the background metric, of

2.108 $$\begin{aligned} F{:}{=} \sqrt{|\det g|} g^{\mu \nu } \big ( r_{\mu \nu } -\frac{2\varLambda }{d} g_{\mu \nu } \big ) \,. \end{aligned}$$

We have, ignoring the usual issues related to the symmetry of $g_{\alpha \beta }$ as this will be taken care of by itself when calculating the Taylor expansion below,

2.109 $$\begin{aligned}&\frac{\partial F}{\partial g_{\alpha \beta }} = -\sqrt{|\det g|} \left( g^{\alpha \mu }g^{\beta \nu }r_{\mu \nu } + \big (\varLambda - \frac{g^{\mu \nu }r_{\mu \nu }}{2}\big ) g^{\alpha \beta } \right) \,, \nonumber \\&\frac{\partial ^2 F}{\partial g_{\rho \sigma }\partial g_{\alpha \beta }}\nonumber \\&\quad = -\sqrt{|\det g|}\left( \frac{1}{2} \left( g^{\alpha \mu }g^{\beta \nu }r_{\mu \nu } + \big (\varLambda - \frac{g^{\mu \nu }r_{\mu \nu }}{2}\big ) g^{\alpha \beta }\right) g^{\rho \sigma }\right. \nonumber \\&\qquad - (g^{\alpha \rho }g^{\mu \sigma }g^{\beta \nu } + g^{\alpha \mu }g^{\beta \rho }g^{\nu \sigma } ) r_{\mu \nu } - \varLambda g^{\alpha \rho } g^{\beta \sigma } \nonumber \\&\qquad \left. + \frac{1}{2} \big ( g^{\mu \rho } g^{\nu \sigma } g^{\alpha \beta } + g^{\mu \nu }g^{\alpha \rho } g^{\beta \sigma } \big )r_{\mu \nu } \right) \,. \end{aligned}$$

Replacing *g* by $g+h$ in the right-hand side of (2.108) we thus obtain the Taylor expansion, after replacing $r_{\mu \nu }$ by $R_{\mu \nu }$ in the result,

2.110 $$\begin{aligned} F= & {} \sqrt{|\det g|} \left[ R - 2 \varLambda - \big (R^{\alpha \beta } + \big (\varLambda -\frac{R}{2}\big ) g^{\alpha \beta }\big ) h_{\alpha \beta } \right. \nonumber \\&- \frac{1}{2}\left( \big (R^{\alpha \beta } +\frac{1}{2} \big (\varLambda - \frac{R}{2}\big ) g^{\alpha \beta }\big )g^{\rho \sigma }\right. \nonumber \\&\left. \left. - 2\big (R^{\alpha \rho } +\frac{1}{2} \big (\varLambda - \frac{R}{2}\big ) g^{\alpha \rho }\big )g^{\beta \sigma } \right) h_{\alpha \beta }h_{\rho \sigma } \right] + O(h^3). \nonumber \\ \end{aligned}$$

Hence

2.111 $$\begin{aligned} Q(h)= & {} \left( 2\big (R^{\alpha \rho } +\frac{1}{2} \big (\varLambda - \frac{R}{2}\big ) g^{\alpha \rho }\big )g^{\beta \sigma }\right. \nonumber \\&\left. - \big (R^{\alpha \beta } +\frac{1}{2} \big (\varLambda - \frac{R}{2}\big ) g^{\alpha \beta }\big )g^{\rho \sigma } \right) h_{\alpha \beta }h_{\rho \sigma } \,. \nonumber \\ \end{aligned}$$

Assuming that the background satisfies the Einstein vacuum equations,

2.112 $$\begin{aligned} R_{\alpha \beta } + \left( \varLambda -\frac{R}{2}\right) g_{\alpha \beta } =0 \quad \Longleftrightarrow \quad R_{\alpha \beta } = \frac{2\varLambda }{d-2} g_{\alpha \beta } \,, \end{aligned}$$

Equation (2.111) simplifies to

2.113 $$\begin{aligned} Q(h) = \frac{2 \varLambda }{(d-2)} \bigg [g^{\alpha \rho }g^{\beta \sigma }h_{\alpha \beta }h_{\rho \sigma }-\frac{1}{2} (g^{\alpha \beta } h_{\alpha \beta })^2\bigg ] \,. \end{aligned}$$

In view of (2.103), the variation of ${\pi }^{\beta \gamma \alpha }$ at $g={b}$ equals

2.114 $$\begin{aligned} \delta {\pi }^{\beta \gamma \alpha }= & {} {\mathfrak {g}}^{\mu \nu } \Big [ 2 \delta {\varGamma }^{{{{{{\chi }}}}}}_{{{{\varphi }}}\nu } \delta ^\rho _\mu - \delta {\varGamma }^{{{{\psi }}}}_{{{{\varphi }}}{{{\psi }}}} \delta ^{{{{{\chi }}}}}_\mu \delta ^\rho _\nu - \delta {\varGamma }^{{{{{{\chi }}}}}}_{\mu \nu } \delta ^{{\rho }}_{{{{\varphi }}} } \Big ] \frac{ \partial {\varGamma }^{{{{\varphi }}}}_{{{{{{\chi }}}}}\rho } }{\partial (\partial _\alpha g_{\beta \gamma })} \nonumber \\= & {} {\frac{1}{16 \pi } } \sqrt{|\det g|} P^{ \alpha (\beta \gamma ) \delta (\epsilon \sigma ) } {\mathring{\nabla }}_{ \delta } \delta g_{\epsilon \sigma } \,. \end{aligned}$$

Given two solutions $\delta _i g$, $i=1,2$, of linearised Einstein equations, the presymplectic current of vacuum Einstein gravity with a cosmological constant therefore reads

2.115 $$\begin{aligned} \omega ^\alpha\equiv & {} \omega ^\alpha (\delta _1 g ,\delta _2 g)\nonumber \\&= {\frac{1}{16 \pi } \sqrt{|\det g|} } P^{ \alpha (\beta \gamma ) \delta (\epsilon \sigma ) }\nonumber \\&\left( \delta _2 g_{ \beta \gamma } { {\mathring{\nabla }}}_{ \delta } \delta _1 g_{\epsilon \sigma } - \delta _1 g_{\beta \gamma } { {\mathring{\nabla }}}_{\delta } \delta _2 g_{\epsilon \sigma }\right) \,. \nonumber \\ \end{aligned}$$

(Because of the symmetrisations occurring in (2.115), one can use there instead an equivalent version of (2.104) given by Wald and Zoupas in [56]:

2.116 $$\begin{aligned} P^{\alpha \beta \gamma \delta \epsilon \sigma }_{WZ}= & {} {{g}}^{\alpha \epsilon } {{g}}^{\sigma \beta } {{g}}^{\gamma \delta } - \frac{1}{2} {{g}}^{\alpha \delta } {{g}}^{\beta \epsilon } {{g}}^{\sigma \gamma } - \frac{1}{2} {{g}}^{\alpha \beta } {{g}}^{\gamma \delta } {{g}}^{\epsilon \sigma }\nonumber \\&- \frac{1}{2} {{g}}^{\beta \gamma } {{g}}^{\alpha \epsilon } {{g}}^{\sigma \delta } + \frac{1}{2} {{g}}^{\beta \gamma } {g}^{\alpha \delta } {{g}}^{\epsilon \sigma } \,. \end{aligned}$$

One can check that the part of the Lagrangian density which contains Christoffel symbols can be reduced to four terms. Indeed, we have

2.117 $$\begin{aligned} P^{ \alpha \beta \gamma \delta \epsilon \sigma } {\mathring{\nabla }}{}_{ \alpha } g_{\beta \gamma } {\mathring{\nabla }}{}_{ \delta } g_{\epsilon \sigma } = \widetilde{P}^{ \alpha \beta \gamma \delta \epsilon \sigma } {\mathring{\nabla }}{}_{ \alpha } g_{\beta \gamma } {\mathring{\nabla }}{}_{ \delta } g_{\epsilon \sigma } \,, \end{aligned}$$

where

2.118 $$\begin{aligned} \widetilde{P}^{\alpha \beta \gamma \delta \epsilon \sigma }&{:}{=}g^{\alpha \sigma } g^{\beta \epsilon } g^{\gamma \delta } -g^{\alpha \beta } g^{\gamma \delta } g^{\epsilon \sigma } +\frac{1}{2} g^{\alpha \delta } g^{\beta \gamma } g^{\epsilon \sigma }\nonumber \\&-\frac{1}{2} g^{\alpha \delta } g^{\beta \sigma } g^{ \gamma \epsilon } \, . \end{aligned}$$

Using (2.117), it follows from (2.105) that

2.119 $$\begin{aligned} {\pi }^{\beta \gamma \alpha } = {\frac{1}{32 \pi } } \sqrt{|\det g|}\left( \widetilde{P}^{\alpha \beta \gamma \delta \epsilon \sigma }+\widetilde{P}^{\delta \epsilon \sigma \alpha \beta \gamma }\right) {\mathring{\nabla }}{}_{\delta } g_{\epsilon \sigma } \; . \end{aligned}$$

Note that one of the terms constituting $\widetilde{P}^{\alpha \beta \gamma \delta \epsilon \sigma }$ is not invariant under exchange of the first three indices with the three last ones:

2.120 $$\begin{aligned} \widetilde{P}^{\alpha \beta \gamma \delta \epsilon \sigma }-\widetilde{P}^{\delta \epsilon \sigma \alpha \beta \gamma }=-g^{\alpha \beta } g^{\gamma \delta } g^{\epsilon \sigma }- \Big (-g^{\delta \epsilon } g^{\sigma \alpha } g^{\beta \gamma }\Big ) \, , \end{aligned}$$

which prevents us to express the canonical momenta in a simple form using $\widetilde{P}^{\alpha \beta \gamma \delta \epsilon \sigma }$.

The following relations hold:

2.121 $$\begin{aligned} P^{\alpha (\beta \gamma ) \delta (\epsilon \sigma )}= & {} P^{\delta (\epsilon \sigma ) \alpha (\beta \gamma )} \,, \end{aligned}$$

2.122 $$\begin{aligned} P^{\alpha (\beta \gamma ) \delta (\epsilon \sigma )}= & {} P^{\alpha (\beta \gamma ) \delta (\epsilon \sigma )}_{WZ} \,,\end{aligned}$$

2.123 $$\begin{aligned} P^{\alpha (\beta \gamma ) \delta (\epsilon \sigma )}= & {} \frac{1}{2} \big ( \widetilde{P}^{\alpha (\beta \gamma ) \delta (\epsilon \sigma )} + \widetilde{P}^{\delta (\epsilon \sigma ) \alpha (\beta \gamma )} \big ) \,. \end{aligned}$$

#### Canonical energy of weak gravitational fields

In the gravitational case (2.292.31) reads

2.124 $$\begin{aligned} {\,\,\,\widetilde{\mathscr {H}}}[\mathscr {S}, X]= & {} \frac{1}{2} \left( \int _\mathscr {S}\omega ^\mu (\delta g , {\mathcal {L}}_X \delta g) \, dS_\mu \right. \nonumber \\&\left. {-} \int _{\partial \mathscr {S}} \tilde{{\pi }}^{ \alpha \beta [\mu } X^{{{\sigma }}]} \delta g_{\alpha \beta } dS_{\sigma \mu } \right) . \end{aligned}$$

From (2.8) and (2.114) we find

2.125 $$\begin{aligned} (\tilde{{\pi }}_A{}^\alpha (\delta \phi ))&\equiv (\tilde{{\pi }}^{\beta \gamma \alpha } (\delta g) )\nonumber \\&= {\frac{1}{16 \pi } } \sqrt{|\det g|} P^{ \alpha (\beta \gamma ) \delta (\epsilon \sigma ) } { {\mathring{\nabla }}}_{ \delta } \delta g_{\epsilon \sigma }. \end{aligned}$$

If $\partial \mathscr {S}$ is a spacelike surface given by the equation

$$\begin{aligned} \{u\equiv x^{0}=\mathrm{const}\,, \ r\equiv x^{ 1}=\mathrm{const}'\} \,, \end{aligned}$$

and if *X* equals $\partial _u$, then the boundary integral in (2.124) reads

2.126 $$\begin{aligned}&- {\frac{1}{32 \pi } } \int _{\partial \mathscr {S}} X^{[\sigma } P^{ \mu ] (\beta \gamma ) \delta (\epsilon \sigma ) } { {\mathring{\nabla }}}_{ \delta } \delta g_{\epsilon \sigma } \, \delta g_{\beta \gamma } \, \sqrt{|\det g|} \, dS_{{{\sigma }}\mu }\nonumber \\&\quad = - {\frac{1}{32 \pi } } \int _{\partial \mathscr {S}} P^{{ r} (\beta \gamma ) \delta (\epsilon \sigma ) } { {\mathring{\nabla }}}_{ \delta } \delta g_{\epsilon \sigma } \, \delta g_{\beta \gamma } \, \sqrt{|\det g|} \, dx^2\wedge dx^3 . \nonumber \\ \end{aligned}$$

If we denote by $h_{\mu \nu }$ the linearised metric field, the “generating equation” (2.18) reads, again with $X=\partial _u$,

2.127 $$\begin{aligned}&{{d {\,\,\,\widetilde{\mathscr {H}}}{[\mathscr {S},X]} }\over {d\lambda }} = \int _\mathscr {S}\big ( {\mathcal {L}}_X h_{\alpha \beta } {{d \tilde{{\pi }}^{\alpha \beta \mu }}\over {d\lambda }} - {\mathcal {L}}_X \tilde{{\pi }}^{\alpha \beta \mu } {{d h_{\alpha \beta }}\over {d\lambda }} \big ) dS_\mu \nonumber \\&\quad {-} {\frac{1}{16 \pi } } \int _{\partial \mathscr {S}} \underbrace{ X^{[\sigma } P^{ \mu ] (\beta \gamma ) \delta (\epsilon \sigma ) } { {\mathring{\nabla }}}_{ \delta } h_{\epsilon \sigma } \, {{d h_{\beta \gamma }}\over {d\lambda }} \, \sqrt{|\det g|} \, dS_{{{\sigma }} \mu } }_ { P^{r (\beta \gamma ) \delta (\epsilon \sigma ) } { {\mathring{\nabla }}}_{ \delta } h_{\epsilon \sigma } \, {{d h_{\beta \gamma }}\over {d\lambda }} \, {\sqrt{|\det g|} \, dx^2\wedge dx^3} } \,. \nonumber \\ \end{aligned}$$

#### Energy flux

In the setting just described, the linearised-fields version of the flux formula (2.36) takes the form

2.128 $$\begin{aligned} \frac{d{\,\,\,\widetilde{\mathscr {H}}}[\mathscr {S}_\tau , X]}{d\tau }= & {} {-} {\frac{1}{16 \pi } } \int _{\partial \mathscr {S}} X^{[\sigma } P^{ \mu ] (\beta \gamma ) \delta (\epsilon \sigma ) } { {\mathring{\nabla }}}_{ \delta } \delta g_{\epsilon \sigma }\nonumber \\&\times {\mathcal {L}}_X \delta g_{\beta \gamma } \, \sqrt{|\det g|} \, dS_{{{\sigma }}\mu } \nonumber \\= & {} {-} {\frac{1}{16 \pi } } \int _{\partial \mathscr {S}_\tau } P^{{ r} (\beta \gamma ) \delta (\epsilon \sigma ) } { {\mathring{\nabla }}}_{ \delta } \delta g_{\epsilon \sigma } \,\nonumber \\&\times {\mathcal {L}}_X\delta g_{\beta \gamma } \, {\sqrt{|\det g|} \, dx^2\wedge dx^3} \,. \end{aligned}$$

#### Gauge invariance

It is shown in [15, Equations (5.19)–(5.20)] that for *solutions of the field equations and for all vector fields* *Y* the current $\mathscr {H}^\mu [Y]$ takes the form $\mathscr {H}^\mu =\partial _\alpha \mathbb {U}^{\mu \alpha } + \mathscr {G}^\mu$, where

2.129 $$\begin{aligned} \mathbb {U}^{\nu \lambda }= & {} {\mathbb {U}^{\nu \lambda }}_{\beta }Y^\beta - \displaystyle \frac{1}{8\pi } \sqrt{|\det g_{\rho \sigma }|} g^{\alpha [\nu }\delta ^{\lambda ]}_\beta {Y^\beta }_{;\alpha } \,, \end{aligned}$$

2.130 $$\begin{aligned} {\mathbb {U}^{\nu \lambda }}_\beta= & {} \displaystyle {\frac{2|\det {b}_{\mu \nu }|}{ 16\pi \sqrt{|\det g_{\rho \sigma }|}}} g_{\beta \gamma }(e^2 g^{\gamma [\lambda }g^{\nu ]\kappa })_{;\kappa } \,, \end{aligned}$$

where a semicolon denotes the covariant derivative of the metric ${b}$, with

2.131 $$\begin{aligned} e\equiv \displaystyle \frac{\sqrt{|\det g_{\rho \sigma }|}}{\sqrt{|\det {b}_{\mu \nu }|}}, \end{aligned}$$

and where $\mathscr {G}^\mu$ does not depend upon the derivatives of *g*.

Under our conditions, inspection of the analysis in [15, Section 5.1] leads to the formula

2.132 $$\begin{aligned} \int _\mathscr {S}{\omega ^\mu ({\mathcal {L}}_Yg,\delta g)} \, dS_\mu= & {} {{d \mathscr {H}_{\mathrm {boundary}} {[\mathscr {S},Y]} }\over {d\lambda }} \nonumber \\\equiv & {} \delta { \mathscr {H}_{\mathrm {boundary}} {[\mathscr {S},Y]} } \,, \end{aligned}$$

where

2.133 $$\begin{aligned} \mathscr {H}_{\mathrm {boundary}} {[\mathscr {S},Y]}&{:}{=}\frac{1}{2} \int _{\partial \mathscr {S}} \mathbb {U}^{\mu \nu } dS_{\mu \nu } \,. \end{aligned}$$

A direct proof of (2.132) will be provided shortly. Thus, for all vector fields *Y* *which vanish together with their first derivatives at* $\partial \mathscr {S}$ and for all variations $\delta g$ of the metric satisfying the linearised field equations it holds that

2.134 $$\begin{aligned} \int _\mathscr {S}{\omega ^\mu ({\mathcal {L}}_Yg,\delta g)} \, dS_\mu =0 \,, \end{aligned}$$

as already established by different arguments in [22, 28, 45]. Since ${\mathcal {L}}_Yg$ is the variation of the metric *g* corresponding to infinitesimal coordinate-transformations, this is interpreted as the statement that *the form obtained by integrating the presymplectic current is gauge-invariant*.

For our purposes the key significance of (2.134) is:

##### Theorem 2

The total Noether charge ${\,\,\,\widetilde{\mathscr {H}}}[\mathscr {S}, X]$ of the linearised gravitational field associated with a compact hypersurface $\mathscr {S}$ with smooth boundary is invariant under the “gauge transformation”

$$\begin{aligned} \delta g \mapsto \delta g + {\mathcal {L}}_Y g \end{aligned}$$

as long as the vector field *Y* satisfies $Y= 0 ={ {\mathring{\nabla }}} Y= [X,Y]={ {\mathring{\nabla }}}([X,Y])$ at $\partial \mathscr {S}$.

##### Proof

Using (2.134) we have

2.135 $$\begin{aligned}&\int _\mathscr {S}\omega ^\mu (\delta g +{\mathcal {L}}_Y g, {\mathcal {L}}_X (\delta g +{\mathcal {L}}_Y g)) \, dS_\mu \nonumber \\&\quad = \int _\mathscr {S}\omega ^\mu (\delta g , {\mathcal {L}}_X (\delta g +{\mathcal {L}}_Y g)) \, dS_\mu \nonumber \\&\qquad +\underbrace{ \int _\mathscr {S}\omega ^\mu ({\mathcal {L}}_Y g , {\mathcal {L}}_X (\delta g +{\mathcal {L}}_Y g)) \, dS_\mu }_{=0} \nonumber \\&\quad = \int _\mathscr {S}\omega ^\mu (\delta g , {\mathcal {L}}_X \delta g ) \, dS_\mu \nonumber \\&\qquad + \int _\mathscr {S}\omega ^\mu (\delta g , { \underbrace{{\mathcal {L}}_Y {\mathcal {L}}_X g + {\mathcal {L}}_{[X,Y]} g) }_{\equiv {\mathcal {L}}_X {\mathcal {L}}_Y g} } \, dS_\mu \nonumber \\&\quad = \int _\mathscr {S}\omega ^\mu (\delta g , {\mathcal {L}}_X \delta g ) \, dS_\mu +\underbrace{\int _\mathscr {S}\omega ^\mu (\delta g , {\mathcal {L}}_Y {\mathcal {L}}_X g )}_{=0}\nonumber \\&\qquad +\underbrace{\int _\mathscr {S}\omega ^\mu (\delta g , {\mathcal {L}}_{[X,Y]} g ) \, dS_\mu }_{=0} \,. \end{aligned}$$

The result follows now from (2.134). $\square$

##### Remark 3

There is an obvious version of Theorem 2 for non-compact $\mathscr {S}$’s, when suitable asymptotic conditions, ensuring the vanishing of the boundary integrals at the right-hand side of (2.132), are imposed on all objects involved. $\square$

#### Proof of (2.132)

The remainder of this section will be devoted to the proof of (2.132). For this it is convenient to write

2.136 $$\begin{aligned} {w}^\mu {:}{=} \frac{16 \pi }{\sqrt{|\det g |}} \omega ^\mu \,. \end{aligned}$$

The presymplectic form on a light cone $\mathscr {C}_u$, which we denote by $\varOmega _{\mathscr {C}_u}$, is obtained by integrating the presymplectic current:

2.137 $$\begin{aligned} \varOmega _{\mathscr {C}_u}(\delta _1g ,\delta _2 g)&{:}{=} \int _{\mathscr {C}_u} \omega ^\mu (\delta _1g ,\delta _2 g) \, dS_\mu \nonumber \\&\equiv \frac{1}{16 \pi } \int _{\mathscr {C}_u} {w}^u (\delta _1g ,\delta _2 g) \underbrace{\sqrt{|\det {g}|} \, dr \, d^2 x^A}_{{=}{:}d\mu _{\mathscr {C}}} \,, \end{aligned}$$

where the light cone is given by the equation $$\{u=0\}$$, and coordinatised by coordinates $$(r,x^A)$$. Thus, to determine $$ \varOmega _{\mathscr {C}_u}(\delta _1g ,\delta _2 g)$$, which in turn determines the volume part of the Noether charge (2.124), we need to calculate $${w}^u $$.

We define

2.138 $$\begin{aligned} b^\mu (\delta _1 g, \delta _2 g){:}{=} P^{ \mu (\beta \gamma ) \delta (\epsilon \sigma ) } \delta _1 g_{\beta \gamma } \nabla _{ \delta } \delta _2 g_{\epsilon \sigma } \,, \end{aligned}$$

so that the vector field ${w}$ of (2.136) equals

2.139 $$\begin{aligned} {w}^\mu (\delta _1 g,\delta _2 g) = b^\mu (\delta _2 g, \delta _1 g) - b^\mu (\delta _1 g, \delta _2 g) \,. \end{aligned}$$

We consider the following gauge transformations

2.140 $$\begin{aligned}&\delta _{1} g \rightarrow \delta _{1} g + {\mathcal {L}}_{\xi _{1}}g \, , \end{aligned}$$

2.141 $$\begin{aligned}&\delta _{2} g \rightarrow \delta _{2} g + {\mathcal {L}}_{\xi _{2}}g \, . \end{aligned}$$

A gauge transformation of the vector field ${w}$ of (2.136) leads to

2.142 $$\begin{aligned}&{w}^{\alpha }(\delta _{1} g + {\mathcal {L}}_{\xi _{1}} g ,\delta _{2} g + {\mathcal {L}}_{\xi _{2}}g )=b^{\alpha } (\delta _2 g,\delta _1 g) \nonumber \\&\quad -b^\alpha (\delta _1 g, \delta _2 g) \nonumber \\&\quad +[ b^{\alpha } (\delta _2 g, {\mathcal {L}}_{\xi _{1}} g) - b^\alpha ({\mathcal {L}}_{\xi _{1}} g, \delta _2 g) ] \nonumber \\&\quad -[ b^{\alpha }(\delta _1 g, {\mathcal {L}}_{\xi _{2}} g) - b^\alpha ({\mathcal {L}}_{\xi _{2}} g, \delta _1 g) ] \nonumber \\&\quad +[ b^{\alpha } ({\mathcal {L}}_{\xi _{2}} g, {\mathcal {L}}_{\xi _{1}} g) - b^\alpha ({\mathcal {L}}_{\xi _{1}} g, {\mathcal {L}}_{\xi _{2}} g) ] \, . \end{aligned}$$

In order to avoid a notational confusion between fields such as $\delta g^{\mu \nu }$, understood as a variation of $g^{\mu \nu }$, and $g^{\mu \alpha }g^{\nu \beta }\delta g_{\alpha \beta }$, as before we will write $h_{\mu \nu }$ for $\delta g_{\mu \nu }$. It is convenient introduce

2.143 $$\begin{aligned} \overline{h}_{\mu \nu }{:}{=} h_{\mu \nu } - \frac{1}{2} g_{\mu \nu } g^{\alpha \beta } h_{\alpha \beta } \, . \end{aligned}$$

Indices on $h_{\mu \nu }$ and $\overline{h}_{\mu \nu }$ are of course raised and lowered with the metric *g*. Each term in square brackets in (2.142) can be rewritten using the identity

2.144 $$\begin{aligned}&[ b^\alpha (h, {\mathcal {L}}_{\xi } g) - b^\alpha ({\mathcal {L}}_{\xi } g, h) ] \nonumber \\&\quad = \nabla _{\beta }\Big [ \big ( g^{\beta \gamma }{\delta ^{\alpha }}_{\sigma } \overline{h}_{\gamma \delta } - g^{\alpha \gamma }{\delta ^{\beta }}_{\sigma }\overline{h}_{\gamma \delta } \big ) \nabla ^{\delta }{\xi }^{\sigma } \Big ]\nonumber \\&\qquad - \nabla _{\beta } \Big [\nabla _{\delta }U^{\alpha \beta }{}_{\gamma }{}^{ \delta } \xi ^{\gamma } \Big ] \nonumber \\&\qquad +2 R_{\delta \gamma } \xi ^{\gamma } \overline{h}^{\alpha \delta } - \!g^{\alpha \beta } ( 2 \delta R_{ \beta \gamma } \!-\! g^{\nu \rho } \delta R_{\nu \rho }g_{\beta \gamma } ) \xi ^{\gamma }, \,\,\,\, \end{aligned}$$

where

2.145 $$\begin{aligned} U^{\alpha \beta \gamma \delta } {:}{=} g^{\beta \delta }{\overline{h}^{\alpha \gamma }} +g^{\alpha \gamma }{\overline{h}^{\beta \delta }} -g^{\beta \gamma }{\overline{h}^{\alpha \delta }} -g^{\alpha \delta }{\overline{h}^{\beta \gamma }} \, . \end{aligned}$$

The tensor $U^{\alpha \beta \gamma \delta }$ fulfills $U^{\mu \lambda \nu \kappa }=U^{[\mu \lambda ][\nu \kappa ]}=U^{\nu \kappa \mu \lambda } \, .$

Note that the last line in (2.144) vanishes on a background which satisfies the vacuum Einstein equations (2.112) and for metric perturbation satisfying the linearised vacuum Einstein equations:

2.146 $$\begin{aligned}&\underbrace{ \nabla ^{\mu } \nabla _{\alpha } \overline{h}_{\beta \mu } +\nabla ^{\mu } \nabla _{\beta } \overline{h}_{\alpha \mu } -\nabla _{\mu } \nabla ^{\mu } \overline{h}_{ \alpha \beta } -g_{\alpha \beta } \nabla ^{\kappa } \nabla ^{\lambda } \overline{h}_{\kappa \lambda } }_{2 \delta R_{\alpha \beta } - g_{\alpha \beta } g^{\nu \rho } \delta R_{\nu \rho }}\nonumber \\&\quad = 2 \varLambda \overline{h}_{\alpha \beta } \, , \end{aligned}$$

Indeed, (2.146) is equivalent to the linearised Einstein equations

2.147 $$\begin{aligned} \delta \big (G_{\mu \nu }+\varLambda g_{\mu \nu } \big ) =0 \, , \end{aligned}$$

when $R_{\mu \nu }=\varLambda g_{\mu \nu }$ holds; see Appendix C.

In order to show (2.144) we start by noting that, by definition of $b^\alpha$, we have

2.148 $$\begin{aligned} b^\alpha (h, {\mathcal {L}}_{\xi } g)= & {} \big ( g^{\beta \gamma }{\delta ^{\alpha }}_{\sigma } \overline{h}_{\gamma \delta } - g^{\alpha \gamma }{\delta ^{\beta }}_{\sigma }\overline{h}_{\gamma \delta } \big ) \nabla _{\beta } \nabla ^{\delta }{\xi }^{\sigma } \nonumber \\&+R^{\alpha }{}_{\delta \beta \sigma } \xi ^{\beta } \overline{h}^{\delta \sigma } +R_{\beta \delta } \xi ^{\beta } \overline{h}^{\delta \alpha } \,. \end{aligned}$$

In order to find $b^\alpha ({\mathcal {L}}_{\xi } g, h)$, we calculate $\nabla _{\beta }\nabla _{\delta }U^{\alpha \beta \gamma \delta }$ and use (2.146) to obtain

2.149 $$\begin{aligned}&\nabla _{\beta }\nabla _{\delta }U^{\alpha \beta \gamma \delta } \nonumber \\&\quad = g^{\beta \delta } \nabla _{\beta } \nabla _{\delta } {\overline{h}^{\alpha \gamma }} +g^{\alpha \gamma } \nabla _{\beta } \nabla _{\delta }{\overline{h}^{\beta \delta }} -g^{\beta \gamma } \nabla _{\beta } \nabla _{\delta }{\overline{h}^{\alpha \delta }} \nonumber \\&\qquad -g^{\alpha \delta } \nabla _{\beta } \nabla _{\delta }{\overline{h}^{\beta \gamma }} \nonumber \\&\quad = -\big (2 \delta R_{\mu \beta } - g_{\mu \beta } g^{\sigma \rho } \delta R_{\sigma \rho }\big ) g^{\mu \alpha } g^{\gamma \beta } -R^{\alpha }{}_{\beta }{}^{\gamma }{}_{\delta } \overline{h}^{\beta \delta }\nonumber \\&\quad + R_{\delta }{}^{\gamma } \overline{h}^{\alpha \delta }.\nonumber \\ \end{aligned}$$

Thus

$$\begin{aligned}&\nabla _{\beta } \Big [\nabla _{\delta }U^{\alpha \beta }{}_{\gamma }{}^{ \delta } \xi ^{\gamma } \Big ] \\&\quad = \nabla _{\beta }\nabla _{\delta }U^{\alpha \beta }{}_{\gamma }{}^{ \delta } \xi ^\gamma + ( \nabla _{\delta }U^{\alpha \beta }{}_{\gamma }{}^{ \delta } ) \nabla _{\beta } \xi ^{\gamma }\\&\quad = -\big (2 \delta R_{\mu \nu } - g_{\mu \nu } g^{\sigma \rho } \delta R_{\sigma \rho }\big ) g^{\mu \alpha } \xi ^\nu \\&\qquad -R^{\alpha }{}_{\beta \gamma \delta } h^{\beta \delta } \xi ^\gamma + R_{\delta \gamma } h^{\alpha \delta } \xi ^\gamma +\big ( \nabla _{\delta }U^{\alpha \beta }{}_{\gamma }{}^{ \delta } \big ) \nabla _{\beta } \xi ^{\gamma }\\&\quad = -\big (2 \delta R_{\mu \nu } - g_{\mu \nu } g^{\sigma \rho } \delta R_{\sigma \rho }\big ) g^{\mu \alpha } \xi ^\nu -R^{\alpha }{}_{\beta \gamma }{}_{\delta } h^{\beta \delta } \xi ^\gamma \\&\qquad + R_{\delta \gamma } h^{\alpha \delta } \xi ^\gamma \\&\qquad + \underbrace{ \big ( g^{\beta \delta } \nabla _{\delta }{\overline{h}^{\alpha }{}_{ \gamma }} +\delta ^{\alpha }{}_{\gamma } \nabla _{\delta }{\overline{h}^{\beta \delta }} -\delta ^{\beta }{}_{ \gamma } \nabla _{\delta }{\overline{h}^{\alpha \delta }} -g^{\alpha \delta } \nabla _{\delta } {\overline{h}^{\beta }{}_{\gamma }} \big ) \nabla _{\beta } \xi ^{\gamma } }_{{=}{:}I } . \end{aligned}$$

Next

2.150 $$\begin{aligned}&\nabla _{\beta }\Big [ \big ( g^{\beta \gamma }{\delta ^{\alpha }}_{\sigma } \overline{h}_{\gamma \delta } - g^{\alpha \gamma }{\delta ^{\beta }}_{\sigma }\overline{h}_{\gamma \delta } \big ) \nabla ^{\delta }{\xi }^{\sigma } \Big ] \nonumber \\&\quad = \underbrace{ \big ( g^{\beta \gamma }{\delta ^{\alpha }}_{\sigma } \nabla _{\beta } \overline{h}_{\gamma \delta } - g^{\alpha \gamma }{\delta ^{\beta }}_{\sigma } \nabla _{\beta } \overline{h}_{\gamma \delta } \big ) \nabla ^{\delta }{\xi }^{\sigma } }_{{=}{:} II} \,,\nonumber \\&\qquad + \big ( g^{\beta \gamma }{\delta ^{\alpha }}_{\sigma } \overline{h}_{\gamma \delta } - g^{\alpha \gamma }{\delta ^{\beta }}_{\sigma }\overline{h}_{\gamma \delta } \big ) \nabla _{\beta } \nabla ^{\delta }{\xi }^{\sigma } \,. \end{aligned}$$

A calculation gives

2.151 $$\begin{aligned} b^\alpha ({\mathcal {L}}_{\xi } g, h)= & {} I - II \end{aligned}$$

Subtracting (2.151) from (2.148) gives (2.144)

To finish the argument it remains to compare (2.132) with (2.144). The variation of the boundary Hamiltonian (2.133) reads

2.152 $$\begin{aligned}&\delta \mathscr {H}_{\mathrm {boundary}} {[\mathscr {S},Y]} = \frac{1}{2} \int _{\partial \mathscr {S}} \delta \mathbb {U}^{\mu \nu } dS_{\mu \nu }= \frac{1}{2} \int _{\partial \mathscr {S}} \Big [ \delta {\mathbb {U}^{\alpha \beta }}_{\gamma }Y^{\gamma } \nonumber \\&\quad - \frac{1}{8\pi } \sqrt{|\det g_{\rho \kappa }|} g^{\mu [\alpha }\delta _{} \overline{g}_{\mu \gamma } \delta ^{\beta ]}_{\sigma } Y^{\sigma ;\gamma } \Big ] dS_{\alpha \beta }\, . \end{aligned}$$

The linearisation of (2.130) gives

2.153 $$\begin{aligned}&\frac{16 \pi }{\sqrt{|\det g_{\rho \kappa }|}} Y^{\gamma } {\mathbb {U}^{\alpha \beta }}_{\gamma }\nonumber \\&= \nabla _{\delta } \Big ( g^{\beta \gamma }{\overline{h}^{\alpha \delta }} +g^{\alpha \delta }{\overline{h}^{\beta \gamma }} -g^{\beta \delta }{\overline{h}^{\alpha \gamma }} -g^{\alpha \gamma }{\overline{h}^{\beta \delta }} \Big ) Y_{\gamma } \, , \end{aligned}$$

2.154 $$\begin{aligned}&\frac{16 \pi }{\sqrt{|\det g_{\rho \kappa }|}} Y^{\gamma } {\mathbb {U}^{\alpha \beta }}_{\gamma } = -\nabla _{\delta }U^{\alpha \beta }{}_{\gamma }{}^{ \delta } \xi ^{\gamma } \, , \end{aligned}$$

which shows that $\delta \mathscr {H}_{\mathrm {boundary}}$ equals the second line in (2.144).

### Adding matter fields

We consider now Einstein equations interacting with matter fields. The fields $\phi ^A$ under consideration take the form

2.155 $$\begin{aligned} \phi ^A = (g_{\mu \nu }, {\phi }^{a}) \,, \end{aligned}$$

where ${\phi }^{a}$ are matter fields. We write the Lagrangian in the form

2.156 $$\begin{aligned} \mathscr {L}= \mathscr {L}_g + \mathscr {L}_m \end{aligned}$$

where $\mathscr {L}_g$ is the Lagrangian (2.98) and $\mathscr {L}_m$ is the Lagrangian describing matter fields, which is assumed to depend upon the metric but not its derivatives. The examples of main interest in the current context would be the Einstein-Maxwell equations, as well as the equations for gravitating elastic bodies.

Assuming that ${\phi }^{a}$ and $\partial _{\mu } {\phi }^{{a}}$ are independent fields, the variation of $\mathscr {L}$ reads

2.157 $$\begin{aligned} \delta \mathscr {L}= \delta \mathscr {L}_H + \frac{\partial \mathscr {L}_m}{\partial \left( \partial _{\mu }{{\phi }^{a}}\right) } \delta \left( \partial _{\mu }{{\phi }^{a}} \right) + \frac{\partial \mathscr {L}_m}{\partial {{\phi }^{a}} } \delta {\phi }^{a}+ \frac{\partial \mathscr {L}_m}{\partial g_{\mu \nu }} \delta g_{\mu \nu } \,, \end{aligned}$$

where $\delta \mathscr {L}_g$ is given by (2.100).

The momenta ${\pi }_A$ split into gravitational and matter parts, with the gravitational momenta given by (2.103), and the matter ones defined as before:

2.158 $$\begin{aligned}&\displaystyle {\pi }_{a}{}^\mu {:}{=} \frac{\partial \mathscr {L}_m}{\partial {\phi }^{a}{}_\mu } \,, \quad {\pi }_{a}{:}{=} \frac{\partial \mathscr {L}_m}{\partial {\phi }^{a}} \,, \end{aligned}$$

2.159 $$\begin{aligned}&\displaystyle {\pi }_{a}{}^\mu {}_{b}{}^\nu {:}{=} \frac{\partial ^2 \mathscr {L}_m}{\partial {\phi }^{a}{}_\mu \partial {\phi }^{b}{}_\nu } \,, \ {\pi }_{a}{}^\mu {}_{b}{:}{=} \frac{\partial ^2 \mathscr {L}_m}{\partial {\phi }^{a}{}_\mu \partial {\phi }^{b}} \,, \ {\pi }_{{a}{b}} {:}{=} \frac{\partial ^2 \mathscr {L}_m}{ \partial {\phi }^{a}\partial {\phi }^{b}} \,. \end{aligned}$$

The Hamiltonian density of linearised fields equals now

2.160 $$\begin{aligned} {\,\,\,\widetilde{\mathscr {H}}}[\mathscr {S}, X]={\,\,\,\widetilde{\mathscr {H}}}_{g} [\mathscr {S}, X]+{\,\,\,\widetilde{\mathscr {H}}}_{m} [\mathscr {S}, X] \,. \end{aligned}$$

According to (2.29), the contribution to the total energy arising from the linearised matter fields equals

$$\begin{aligned} {\,\,\,\widetilde{\mathscr {H}}}_m [\mathscr {S}, X]= & {} \frac{1}{2} \left( \int _\mathscr {S}\omega ^\mu (\tilde{{\phi }}, {\mathcal {L}}_X \tilde{{\phi }}) \, dS_\mu \right. \\&\left. - \int _{\partial \mathscr {S}} X^{[{{\sigma }}} \tilde{{\pi }}_{a}{}^{\mu ]} \tilde{{\phi }}^{a}dS_{{{\sigma }}\mu } \right) \,, \end{aligned}$$

where $\omega ^\mu (\tilde{{\phi }}, {\mathcal {L}}_X \tilde{{\phi }})= {\mathcal {L}}_X \tilde{{\phi }}^{a}\, \tilde{{\pi }}_{a}{}^\mu - {\mathcal {L}}_X \tilde{{\pi }}_{a}{}^\mu \tilde{{\phi }}^{a}$. From (2.36), the energy flux formula for matter fields is equal to

2.161 $$\begin{aligned} \frac{d\mathscr {H}_m (\mathscr {S}_\tau , X)}{d\tau } = - \int _{\partial \mathscr {S}_\tau } X^{[{{\sigma }}} {\pi }_{a}{}^{\mu ]} {\mathcal {L}}_X{\phi }^{a}dS_{{{\sigma }} \mu } \,. \end{aligned}$$

#### The presymplectic form on null hypersurfaces

#### General gauge

While we are mainly interested in families of light cones in this work, the calculations that follow apply to any null hypersurfaces. Hence we consider a set of coordinates which is adapted to a space-time foliation by null hypersurfaces. The null generator of each surface is proportional to $\partial _{r}$. We will write the space-time metric in adapted coordinates as

2.162 $$\begin{aligned} g = g_{uu} du^2-2 e^{2\beta }du\,dr + 2 g_{uA} dx^A +g_{AB} dx^A dx^B \, . \nonumber \\ \end{aligned}$$

In terms of coordinate components, the field $b^{u}$ defined in (2.138) takes the form

2.163 $$\begin{aligned}&b^u(\delta _1 g,\delta _2 g)\nonumber \\&\quad = \frac{ e^{-4 \beta }\delta _{1}g_{u r} }{2}g^{A B} \left( \nabla _{r}\delta _{2} g_{A B}-2 \nabla _{A} \delta _{2}g_{r B} \right) \nonumber \\&\qquad +\frac{e^{-2 \beta }\delta _{1} g_{A B}}{2 } \Big [ g^{A B} \left( g^{C D}\left( \nabla _{C}\delta _{2} g_{r D}- \nabla _{r} \delta _{2} g_{C D} \right) \right. \nonumber \\&\qquad \left. +e^{-2 \beta } \nabla _{r} \delta _{2} g_{u r} \right) \nonumber \\&\qquad + g^{A C} g^{B D} \left( \nabla _{r} \delta _{2} g_{CD}- 2 \nabla _{C}\delta _{2}g_{r D} \right) \Big ] \,. \end{aligned}$$

While we use the notation (2.162) for the components of the metric at which the variations are taking place, most of the time we simply use $\delta g_{\mu \nu }$ for the variations, which are assumed to satisfy

$$\begin{aligned} \delta g_{rr}=\delta g_{rA}=0 \,. \end{aligned}$$

Note that $\nabla _{A} \delta _{2}g_{r B}$ will not be zero in general even though $\delta g_{rA}$ vanishes. Writing-out the Christoffel symbols, we find

2.164 $$\begin{aligned}&b^{u}(\delta _1 g,\delta _2 g)\nonumber \\&\quad = \frac{ e^{-6 \beta }\delta _1 g_ {u r}}{2 } g^{AB} \left( \delta _2 g_ {u r} \partial _{r} g_{AB}+e^{2 \beta } \partial _r \delta _{2}g_{AB} \right) \nonumber \\&\qquad + e^{-4 \beta }\delta _{1}g_{AB} \left\{ g^{AB} \left[ \frac{1}{2} \partial _{r} \delta _{2} g_{u r} -\delta _{2} g_{u r}\right. \right. \nonumber \\&\qquad \times \left( \frac{1}{4} g^{CD}\partial _{r} g_{CD} +\partial _{r} \beta \right) +\frac{e^{2 \beta }}{4}\nonumber \\&\qquad \times \left. \left( \delta _{2}g_{CD}g^{CE}g^{DF} \partial _{r} g_{EF}-2 g^{CD} \partial _{r} \delta _{2}g_{CD} \right) \right] \nonumber \\&\qquad + \left. \frac{g^{AC} g^{BD}}{2} \left( e^{2 \beta } \partial _{r} \delta _{2} g_{CD}+ \delta _{2} g_{u r} \partial _{r} g_{CD} \right) \right\} \,. \end{aligned}$$

Antisymmetrising over $\delta _1g$ and $\delta _2g$ to obtain the field ${w}^u$ of (2.136), the first term at the right-hand side drops out, which is the only obvious simplification. If we assume moreover the Bondi condition

2.165 $$\begin{aligned} g^{AB}\delta g_{AB} = 0 \,, \end{aligned}$$

we find

2.166 $$\begin{aligned}&{w}^u (\delta _1 g,\delta _2 g)\nonumber \\&\quad = \frac{ e^{-4 \beta }}{2 } \bigg [\delta _2 g_ {u r} g^{AB} \partial _r \delta _{1}g_{AB}- \delta _1 g_ {u r} g^{AB} \partial _r \delta _{2}g_{AB} \nonumber \\&\qquad + g^{AC} g^{BD}\big ( \delta _{2}g_{AB}(e^{2 \beta } \partial _{r} \delta _{1} g_{CD}+ \delta _{1} g_{u r} \partial _{r} g_{CD} ) \nonumber \\&\qquad \left. -\delta _{1}g_{AB} (e^{2 \beta } \partial _{r} \delta _{2} g_{CD}+ \delta _{2} g_{u r} \partial _{r} g_{CD} )\big ) \right] \,. \end{aligned}$$

Using

$$\begin{aligned}&\delta _2 g_ {u r} g^{AB} \partial _r \delta _{1}g_{AB}-\delta _{1}g_{AB} \delta _{2} g_{u r} g^{AC} g^{BD} \partial _{r} g_{CD}\\&\quad =\delta _2 g_ {u r} \partial _{r} \left( g^{AB}\delta _{1}g_{AB}\right) =0 \end{aligned}$$

we obtain

2.167 $$\begin{aligned}&{w}^u (\delta _1 g,\delta _2 g)\nonumber \\&\quad =\frac{ e^{-2 \beta }}{2 } g^{AC} g^{BD} \left( \delta _{2}g_{AB} \partial _{r} \delta _{1} g_{CD}-\delta _{1}g_{AB} \partial _{r} \delta _{2} g_{CD}\right) \,. \nonumber \\ \end{aligned}$$

Explicit formulae for the field $b^r$ can be found in Appendix B, see also (5.21).

#### The nonlinear theory

Let us denote by *K* a family of future directed generators of $\mathscr {N}$. We choose the orientation of Bondi coordinates so that $K=f \partial _{r}$ where $f>0$. For each point $p \in \mathscr {N}$ the tangent space $T_p \mathscr {N}$ may be quotiented by the subspace spanned by *K*. This quotient space $T_p \mathscr {N}/K$ carries a non-degenerate Riemannian metric *h* and, therefore, is equipped with a volume form $\omega$. Consider a two-form $\mathbf{L}$ which is equal to the pull-back of $\omega$ from the quotient space $T_p \mathscr {N}/K$ to $T_p \mathscr {N}$

$$\begin{aligned} \pi : T_p \mathscr {N}\longrightarrow T_p \mathscr {N}/K \, , \quad \mathbf{L}{:}{=}\pi ^*\omega \, . \end{aligned}$$

We choose a one-form $\alpha$ on *N*, such that $<K,\alpha > \equiv 1$, and define a three-form $\mathbf{v}_K$ as the product

$$\begin{aligned} \mathbf{v}_K = \alpha \wedge \mathbf{L} \, . \end{aligned}$$

Note that $\mathbf{v}_K$ does not depend upon $\alpha$ because $K\wedge \mathbf{L} =0$. We can write

2.168 $$\begin{aligned} \mathbf{v}_K =v_{K} dr\wedge dx^2\wedge dx^3\,, \ \text{ where } \ v_K=\frac{\sqrt{\mathrm {det} \, g_{AB}}}{f}. \end{aligned}$$

We define the following vector density

2.169 $$\begin{aligned} {\varPi }=v_{K} K \equiv \sqrt{\mathrm {det} \, g_{AB}} \partial _r \, , \end{aligned}$$

which is equivalent to the equation

2.170 $$\begin{aligned} \mathbf{L} = {\varPi }^a \left( \partial _a \ \rfloor \ dr \wedge dx^2 \wedge dx^3 \right) \, . \end{aligned}$$

Following [39], we define the tensor density

2.171 $$\begin{aligned} {{ {Q}}^a}_b (K) {:}{=} -s \left\{ v_K \left( \nabla _b K^a - \delta _b^a \nabla _c K^c \right) + \delta _b^a \partial _c { {\varPi }^c} \right\} \, , \end{aligned}$$

where

$$\begin{aligned} s{:}{=}\mathrm{sgn}g^{u r}=\pm 1 \,, \end{aligned}$$

thus *s* equals minus one if both $\partial _u$ and $\partial _r$ are causal and consistently time-oriented. (In the case where $\partial _u$ changes type, as happens for light cones in the de Sitter metric when $\partial _u$ is taken to be a timelike Killing vector near the vertex of the cone, the value of *s* is determined near the vertex and extended to the light cone by continuity.) Choosing

$$\begin{aligned} K{:}{=}\mathrm{e}^{-2\beta } \partial _r \end{aligned}$$

and assuming that the variations of the metric preserve the Bondi form of the metric (including the determinant condition), the pre-symplectic form can be rewritten as [39]

2.172 $$\begin{aligned}&\varOmega _{\mathscr {N}} (\delta _1g, \delta _2 g)\nonumber \\&\quad = \frac{1}{16 \pi } \int _{\mathscr {N}} ( \delta _1 {Q}^{AB} \delta _2 g_{AB} - \delta _2 {Q}^{AB} \delta _1 g_{AB} ) \, d^3 x \,, \end{aligned}$$

where ${Q}$ is given by

2.173 $$\begin{aligned} {Q}^{A B}&{:}{=}g^{BC} {Q}^A{}_C(K) \equiv \frac{s}{2} {\sqrt{\mathrm {det} \, g_{EF}}} \, g^{AC}g^{BD} \partial _r g_{CD} .\nonumber \\ \end{aligned}$$

The reader is warned that the field ${{Q}^{a}}_{b}$ is not invariant under rescalings of the null generator *K*. Linearising (2.172)–(2.173) provides another derivation of (2.167). Compare [44].

## Bondi gauge

In this section we show how to put a linearised metric perturbation in Bondi gauge, and analyse the gauge freedom remaining.

### Coordinate transformations, gauge freedom

Linearised gravitational fields are defined up to a gauge transformation

3.1 $$\begin{aligned} h\mapsto h+ {\mathcal {L}}_{\zeta }g \end{aligned}$$

determined by a vector field ${\zeta }$. The aim of this section is to analyse the gauge transformations which bring a smooth linearised solution *h* of the vacuum Einstein equations to the Bondi gauge. We will assume that, near the conformal boundary at future infinity, the linearised solution behaves as if it arose from a one-parameter of smoothly conformally compactifiable solutions near the de Sitter metric. Thus we take a background of the form

3.2 $$\begin{aligned} g= \epsilon {N}^2 du^2 - 2 du \, dr + r^2 {\mathring{{\gamma }}}_{AB}dx^A dx^B \,, \end{aligned}$$

where ${N}$ depends only upon *r*, with $\epsilon \in \{\pm 1\}$, and where $\partial _u {\mathring{{\gamma }}}_{AB}=0=\partial _r{\mathring{{\gamma }}}_{AB}$.

It turns out that the transformation to Bondi gauge introduces singularities at the vertex. For this reason in this section in formulae where ambiguities might arise, *and only these formulae*, we will write ${h^{\mathrm{reg}}}$ for the metric perturbation in the original manifestly smooth gauge (where “reg” stands for “regular”) in the cone-adapted coordinates $(u,r,x^A)$, and we will write ${h^{\mathrm{Bo}}}$ for the metric in the Bondi gauge. For instance, in order not to overburden the notation we will continue to write $h_{tt}$, $h_{ti}$ and $h_{ij}$ instead of ${h^{\mathrm{reg}}}_{tt}$, ${h^{\mathrm{reg}}}_{ti}$ and ${h^{\mathrm{reg}}}_{ij}$ in the original manifestly smooth coordinates $(t,x^i)$, since the metric in the Bondi coordinates will only be considered in the $(u,r,x^A)$–coordinate system.

The “infinitesimal coordinate transformations” (3.1) should transform the metric perturbation to the Bondi gauge:

3.3 $$\begin{aligned} {\mathcal {L}}_{{\zeta }} g_{{r}{r}} + {h^{\mathrm{reg}}}_{{r}{r}}= & {} 0 \,, \end{aligned}$$

3.4 $$\begin{aligned} {\mathcal {L}}_{{\zeta }} g_{{r}A} + {h^{\mathrm{reg}}}_{{r}A}= & {} 0 \,,\end{aligned}$$

3.5 $$\begin{aligned} g^{AB} ({\mathcal {L}}_{{\zeta }} g_{AB} + {h^{\mathrm{reg}}}_{AB} )= & {} 0 \, . \end{aligned}$$

The last condition deserves a justification. For this, consider a one-parameter family of metrics, say $\lambda \mapsto g(\lambda )$ in Bondi coordinates. (In the current case of interest $\lambda$ is the flow parameter along the vector field $\zeta$, but the argument applies to any such family.) We then have

3.6 $$\begin{aligned} g(\lambda )_{22} g(\lambda )_{33} - g_{23}(\lambda )^2 = r^4 \sin ^2\theta \,. \end{aligned}$$

Differentiating with respect to $\lambda$ one finds

3.7 $$\begin{aligned} g^{AB}h_{AB}\equiv g^{AB} \frac{d g_{AB}}{d\lambda }= 0 \,. \end{aligned}$$

After performing a gauge transformation (3.1), in the new gauge we must likewise have

3.8 $$\begin{aligned} g^{AB}(h_{AB} + {\mathcal {L}}_{{\xi }} g_{AB}) =0 \,, \end{aligned}$$

which explains (3.5).

The conditions (3.3)–(3.4) are equivalent to:

$$\begin{aligned} \partial _{{r}} {\zeta }^{{u}} - \frac{1}{2} {h^{\mathrm{reg}}}_{{r}{r}}= & {} 0 \,,\\ \partial _{{r}}{\zeta }^{A}- \frac{{{\mathring{{\gamma }}}}^{AB}}{{r}^2} \left( \partial _{B} {\zeta }^{{u}} - {h^{\mathrm{reg}}}_{{r}B} \right)= & {} 0 \,, \end{aligned}$$

which is solved by

3.9 $$\begin{aligned} {\zeta }^{{u}}(u,r,x^A)= & {} {\xi }^{{u}}({u},x^A) + \frac{1}{2} \int _0^r {h^{\mathrm{reg}}}_{rr} (u,s,x^A) \, ds \,, \end{aligned}$$

3.10 $$\begin{aligned} {\zeta }^{B}(u,r,x^A)= & {} {\xi }^{B} ({u},x^A) -{{\mathring{{\gamma }}}}^{BC} \left( \frac{1}{{r}} \partial _{C} {\xi }^{{u}}({u},x^A) \right. \nonumber \\&+ \int _{r_0}^r \frac{1}{s^{2}} \left( {h^{\mathrm{reg}}}_{rC}(u,s,x^A) \right. \nonumber \\&\left. \left. - \frac{1}{2} \int _0^s\!\partial _{C} {h^{\mathrm{reg}}}_{rr}(u,\rho , x^A)\, d\rho \right) ds \right) , \, \end{aligned}$$

for some fields ${\xi }^{u} ({u},x^A)$, ${\xi }^{B} ({u},x^A)$, and where $r_0$ can be chosen conveniently according to the context.

In order to address (3.5) we will use the symbol $\mathring{{\mathcal {L}}}_{\zeta }$ to denote Lie-derivation in the $x^A$-variables with respect to the vector field ${\zeta }^A\partial _A$. We have

3.11 $$\begin{aligned}&{r}^{-2} {{\mathring{{\gamma }}}}^{AB} {\mathcal {L}}_{{\zeta }} g_{AB}\nonumber \\&\quad = {r}^{-2} {{\mathring{{\gamma }}}}^{AB} \left( 2 {r}{\zeta }^{{r}} {{\mathring{{\gamma }}}}_{AB} + {\zeta }^{C}\partial _{C}g_{AB} + 2 {\zeta }^{C}{}_{,(A}g_{B) C} \right) \nonumber \\&\quad = {r}^{-2} {{\mathring{{\gamma }}}}^{AB} \left( 2 {r}{\zeta }^{{r}} {{\mathring{{\gamma }}}}_{AB} + {r}^2 \mathring{{\mathcal {L}}}_{{\zeta }} {{\mathring{{\gamma }}}}_{AB} \right) \nonumber \\&\quad = {r}^{-2} \left( 4 {r}{\zeta }^{{r}} + 2 {r}^2 \mathring{D}_{B} {\zeta }^{B} \right) \nonumber \\&\quad = - {r}^{-2} {{\mathring{{\gamma }}}}^{AB} {h^{\mathrm{reg}}}_{AB} \, , \end{aligned}$$

where in the last line we used (3.5). Hence

3.12 $$\begin{aligned} {\zeta }^{{r}} = -\frac{1}{2} {r}\mathring{D}_{B} {\zeta }^{B} - \frac{1}{4} {r}^{-1} {{\mathring{{\gamma }}}}^{AB} {h^{\mathrm{reg}}}_{AB} \,. \end{aligned}$$

We will denote by

$$\begin{aligned} {\zeta }[{h^{\mathrm{reg}}}] \equiv {\zeta }|_{{\xi }^A={\xi }^u =0} \end{aligned}$$

the part of ${\zeta }$ which depends explicitly upon *h*, and write

3.13 $$\begin{aligned} {\zeta }^{\mu }= {\zeta }^{\mu } [{h^{\mathrm{reg}}}] + \mathring{{\zeta }}^{\mu } \,. \end{aligned}$$

We note that $\mathring{{\zeta }}$ still contains a part which depends upon *h*, as needed to satisfy the asymptotic boundary conditions. This is discussed in more detail in Sect. 3.1.2. The remaining part of $\mathring{{\zeta }}$ describes asymptotic symmetries, we return to this in Sect. 3.2.1.

#### Small *r*

An analysis of the behavior of the metric at the tip of the light cone is in order. For definiteness consider a smooth metric perturbation of Minkowski, Anti de Sitter or de Sitter spacetime. After transforming to Bondi coordinates of the background metric we have for small *r*

3.14 $$\begin{aligned}&{h^{\mathrm{reg}}}_{rr} = O(1) \,, \quad {h^{\mathrm{reg}}}_{rA} = O(r ) \,, \quad {h^{\mathrm{reg}}}_{AB} = O(r^2) \,, \nonumber \\&{h^{\mathrm{reg}}}_{ur} = O(1) \,, \quad {h^{\mathrm{reg}}}_{uA} = O(r ) \,, \quad {h^{\mathrm{reg}}}_{uu} = O(1) \,. \end{aligned}$$

Equation (3.9) gives, for small *r*,

3.15 $$\begin{aligned} {\zeta }[{h^{\mathrm{reg}}}]^u = O(r) \,. \end{aligned}$$

Now, there could be a 1/*r* term for small *r* in the integral defining $\zeta ^A$, which could lead to logarithmic terms. To see that there is a cancellation, we note that

3.16 $$\begin{aligned} {h^{\mathrm{reg}}}_{rr}= & {} h_{tt} + 2 h_{ti}\frac{x^i}{r} + h_{ij}\frac{x^i x^j}{r^2} \,, \end{aligned}$$

3.17 $$\begin{aligned} {h^{\mathrm{reg}}}_{rA}= & {} \left( h_{tj} + h_{ij} \frac{x^i}{r} \right) \frac{\partial x^j}{\partial x^A} \,,\end{aligned}$$

3.18 $$\begin{aligned} {h^{\mathrm{reg}}}_{ur}= & {} h_{tt} +h_{ti}\frac{x^i}{r} \,. \end{aligned}$$

Then

3.19 $$\begin{aligned}&{h^{\mathrm{reg}}}_{rC} - \frac{1}{2} \int _{0}^{r }\partial _C {h^{\mathrm{reg}}}_{rr} dr \nonumber \\&\quad = \left( h_{tj} + h_{ij} \frac{x^i}{r} \right) \frac{\partial x^j}{\partial x^A} \nonumber \\&\qquad - \frac{1}{2} \int _0^r \partial _C \left( h_{tt} + 2 h_{ti}\frac{x^i}{r} + h_{ij}\frac{x^i x^j}{r^2} \right) dr \nonumber \\&\quad = \int _0^r \left[ \frac{d}{dr } \left( \left( h_{tj} + h_{ij} \frac{x^i}{r} \right) \frac{\partial x^j}{ \partial x^C} \right) \right. \nonumber \\&\qquad \left. - \frac{1}{2} \partial _C \left( h_{tt} + 2 h_{ti}\frac{x^i}{r} + h_{ij}\frac{x^i x^j}{r^2} \right) \right] dr \nonumber \\&\quad = \int _0^r \bigg ( (\partial _k h_{t j}) \frac{x^k}{r} \frac{\partial x^j}{ \partial x^C} +\frac{1}{r} h_{t j} \frac{\partial x^j}{ \partial x^C} - \frac{1}{2} (\partial _k h_{tt}) \frac{\partial x^{k}}{\partial x^C} \nonumber \\&\qquad - (\partial _k h_{tj}) \frac{x^{j}}{r} \frac{\partial x^{k}}{\partial x^C} -\frac{1}{r} h_{t j} \frac{\partial x^j}{ \partial x^C} \bigg ) dr + \int _0^r \bigg ( (\partial _k h_{ij}) \frac{x^k }{r }\frac{x^i }{r } \nonumber \\&\qquad \times \frac{\partial x^j}{ \partial x^C}+h_{ij} \frac{x^i }{r^2 } \frac{\partial x^j}{ \partial x^C} -\frac{1}{2} \partial _ C\big ( h_{ij } \frac{x^ix^j }{r^2 } \big ) \bigg ) dr \nonumber \\&\quad = \int _0^r \bigg ( 2(\partial _{[k} h_{ j] t}) \frac{x^k}{r} \frac{\partial x^j}{ \partial x^C} - \frac{1}{2} (\partial _k h_{tt}) \frac{\partial x^{k}}{\partial x^C} \bigg ) dr \nonumber \\&\qquad + \int _0^r \bigg ( (\partial _k h_{ij}) \bigg ( \frac{x^k }{r } \frac{\partial x^j}{ \partial x^C} - \frac{1}{2} \frac{x^j }{r } \frac{\partial x^k }{\partial x^C }\bigg )\frac{x^i }{r } \bigg )dr \nonumber \\&\quad = O(r^2) \,, \end{aligned}$$

and (3.10) gives, again for small *r*,

3.20 $$\begin{aligned}&{\zeta }^{B}(u,r,x^A) \nonumber \\&\quad = {\xi }^{B} ({u},x^A) -{{\mathring{{\gamma }}}}^{BC} \bigg [ \frac{1}{{r}} \partial _{C} {\xi }^{{u}}({u},x^A) \nonumber \\&\qquad + \frac{1}{2} \partial _k h_{ij}\big |_{r=0} \bigg ( \frac{x^k }{r} \frac{\partial x^j}{ \partial x^C} - \frac{1}{2} \frac{x^j }{r} \frac{\partial x^k }{\partial x^C }\bigg )\frac{x^i }{r } \bigg ] + O(r^2) \nonumber \\&\quad = O(r^{-1}) \,, \end{aligned}$$

so that

3.21 $$\begin{aligned} {\zeta }[h]^A = O(r) \,. \end{aligned}$$

Equations (3.9)–(3.10) together with (3.12) lead to, again for small *r*,

3.22 $$\begin{aligned}&{\zeta }[h]^r = O(r ) \,, \end{aligned}$$

3.23 $$\begin{aligned}&{\xi }^u = O(1) \,, \quad {\xi }^A = O(1 ) \,, \quad \zeta ^u = {\xi }^u + O(r) \,, \end{aligned}$$

3.24 $$\begin{aligned}&\displaystyle \zeta ^A = {\xi }^{A} ({u},x^B) - \frac{1}{{r}} \mathring{D}^A {\xi }^{{u}}({u},x^B) + O(r) \,. \end{aligned}$$

Inserting (3.10) in (3.12) we find

3.25 $$\begin{aligned}&\mathring{\zeta }^r= \frac{{\varDelta _{{{\mathring{{\gamma }}}}}}{\xi }^{{u}}}{2} - \frac{{r}\mathring{D}_{B} {\xi }^{B}}{2} = O(1) \, , \quad \nonumber \\&{\zeta }^r = \frac{1}{2} \varDelta _{{\mathring{{\gamma }}}}\xi ^u + O(r ) = O(1) \,, \end{aligned}$$

where ${\varDelta _{{{\mathring{{\gamma }}}}}}$ is a Laplace operator associated with the metric ${{\mathring{{\gamma }}}}_{AB}$.

The behaviour of various derivatives should be clear from the above.

We emphasise that the original behaviour (3.14) of *h* near the vertex will not be true in general for the metric coefficients in Bondi coordinates. For instance:

3.26 $$\begin{aligned} {h^{\mathrm{Bo}}}_{uA}= & {} {h^{\mathrm{reg}}}_{uA} + \mathscr {L}_\zeta g_{uA} = {h^{\mathrm{reg}}}_{uA} + \partial _A\zeta ^\mu \eta _{u\mu } + \partial _u\zeta ^\mu \eta _{\mu A} \nonumber \\= & {} {h^{\mathrm{reg}}}_{uA} + \partial _A(\epsilon N^2 \zeta ^u - \zeta ^r) + r^2 {{\mathring{{\gamma }}}}_{AB} \partial _u\zeta ^B \nonumber \\= & {} - \mathring{D}_A \left( 1+ \frac{1}{2} \varDelta _{{\mathring{{\gamma }}}}\right) \xi ^u + O(r) \, \nonumber \\= & {} O(1) \,, \end{aligned}$$

3.27 $$\begin{aligned} {h^{\mathrm{Bo}}}_{ur}= & {} {h^{\mathrm{reg}}}_{ur} + \mathscr {L}_\zeta g_{ur} = {h^{\mathrm{reg}}}_{ur} - \partial _u \zeta ^u + \epsilon N^2 \partial _r \zeta ^u - \partial _r \zeta ^r \nonumber \\= & {} {h^{\mathrm{reg}}}_{ur} - \partial _u \xi ^u - \frac{1}{2 } {h^{\mathrm{reg}}}_{rr} + \frac{1}{2} \mathring{D}^A \xi _A \nonumber \\&+ \frac{1}{4} \partial _r(r^{-1} {{\mathring{{\gamma }}}}^{AB}{h^{\mathrm{reg}}}_{AB}) + O(r) \,, \end{aligned}$$

3.28 $$\begin{aligned} { {h^{\mathrm{Bo}}}_{uu}}= & {} { {h^{\mathrm{reg}}}_{uu} + \mathscr {L}_\zeta g_{uu} = {h^{\mathrm{reg}}}_{uu} + \epsilon {\zeta }^{{r}} \partial _r N^2 } \nonumber \\&+2 \partial _u(\epsilon N^2 \zeta ^u - \zeta ^r) \nonumber \\= & {} {h^{\mathrm{reg}}}_{uu} - (2 + \varDelta _{{\mathring{{\gamma }}}}) \partial _u\xi ^u + O(r) \,, \end{aligned}$$

3.29 $$\begin{aligned} {h^{\mathrm{Bo}}}_{AB}= & {} {h^{\mathrm{reg}}}_{AB} + \mathscr {L}_\zeta g_{AB} = {h^{\mathrm{reg}}}_{AB} +2 {r}{\zeta }^{{r}} {{\mathring{{\gamma }}}}_{AB} + {r}^2 \mathring{{\mathcal {L}}}_{{\zeta }} {{\mathring{{\gamma }}}}_{AB}\nonumber \\= & {} {r}(\varDelta _{{\mathring{{\gamma }}}}\xi ^u {{\mathring{{\gamma }}}}_{AB} - 2 \mathring{D}_A\mathring{D}_B \xi ^u ) + O(r^2) \,. \end{aligned}$$

Summarising, for small *r*,

3.30 $$\begin{aligned}&{h^{\mathrm{Bo}}}_{uu}= O(1) \,, \quad {h^{\mathrm{Bo}}}_{ur} = O(1) \,, \quad {h^{\mathrm{Bo}}}_{uA} = O(1) \,, \quad \nonumber \\&{h^{\mathrm{Bo}}}_{AB} =O(r) \,. \end{aligned}$$

For further reference we emphasise that

3.31 $$\begin{aligned} \mathring{D}^A {h^{\mathrm{Bo}}}_{uA}|_{r=0} = - \frac{1}{2} (\varDelta _{{\mathring{{\gamma }}}}+ 2) \varDelta _{{\mathring{{\gamma }}}}\xi ^u \,. \end{aligned}$$

Using

3.32 $$\begin{aligned}&\mathring{\gamma }^{AB}\frac{\partial x^{i}}{\partial x^{A}}\frac{\partial x^{j}}{\partial x^{B}} = \left( \delta ^{ij} - \frac{x^{i}x^{j}}{r^{2}}\right) r^{2} \end{aligned}$$

3.33 $$\begin{aligned}&\varDelta _{{\mathring{{\gamma }}}}x^i = -2 x^i \,, \quad 0 = \varDelta _{{\mathring{{\gamma }}}}(\underbrace{x^ix^i}_{r^2}) = 2 \mathring{D}^A(x^i \mathring{D}_A x^i) \,, \end{aligned}$$

3.34 $$\begin{aligned}&\displaystyle \mathring{D}^C \bigg ( \frac{x^{j} }{r^2}\frac{\partial x^{i}}{\partial x^C} \bigg ) = \frac{ \mathring{D}^C x^{j} }{r^2}\frac{\partial x^{i}}{\partial x^C} + \frac{x^{j} }{r^2} \varDelta _{{\mathring{{\gamma }}}}x^{i} = \delta ^{ij} - \frac{3 x^i x^j}{r^2} \,, \nonumber \\ \end{aligned}$$

3.35 $$\begin{aligned}&\displaystyle \mathring{D}^C \bigg ( \frac{x^{(j} }{r^2}\frac{\partial x^{i)}}{\partial x^C} \bigg ) = \delta ^{ij} - \frac{3 x^i x^j}{r^2}\,, \quad \varDelta _{{\mathring{{\gamma }}}}\frac{x^i x^j}{r^2}= 2 \delta ^{ij} -6 \frac{x^i x^j}{r^2} \,, \nonumber \\ \end{aligned}$$

Equation (3.27) can be rewritten as

3.36 $$\begin{aligned} {h^{\mathrm{Bo}}}_{ur}= & {} {h^{\mathrm{reg}}}_{ur}|_{r=0} - \partial _u \xi ^u + \frac{1}{2} \mathring{D}^A \xi _A \nonumber \\&- \frac{1}{2} \left( h_{tt}|_{r=0} + 2 h_{ti}|_{r=0}\frac{x^i}{r} + h_{ij}|_{r=0}\frac{x^i x^j}{r^2} \right) \nonumber \\&+ \frac{1}{4} \partial _r \bigg ( \underbrace{ r^{-1} {{\mathring{{\gamma }}}}^{AB}h_{ij}\frac{\partial x^{i}}{\partial x^{A}}\frac{\partial x^{j}}{\partial x^{B}} }_{r h_{ij} \left( \delta ^{ij} -\frac{x^ix^j}{r^2}\right) } \bigg ) + O(r) \nonumber \\= & {} {h^{\mathrm{reg}}}_{ur}|_{r=0} - \partial _u \xi ^u + \frac{1}{2} \mathring{D}^A \xi _A \nonumber \\&- \frac{1}{2} \Big ( h_{tt}|_{r=0} + 2 h_{ti}|_{r=0}\frac{x^i}{r} \Big ) \nonumber \\&+\frac{1}{4} h_{ij}|_{r=0} \left( \delta ^{ij} -\frac{x^ix^j}{r^2}\right) - \frac{1}{2} h_{ij}|_{r=0}\frac{x^ix^j}{r^2} + O(r) \nonumber \\= & {} \frac{1}{2} h_{tt}|_{r=0} - \partial _u \xi ^u + \frac{1}{2} \mathring{D}^A \xi _A \nonumber \\&+\frac{1}{4} h_{ij}|_{r=0} \left( \delta ^{ij} -3\frac{x^ix^j}{r^2}\right) + O(r) \,. \end{aligned}$$

#### From smooth to Bondi

In view of the formulae so far, one proceeds as follows. Given a smooth linearised metric perturbation *h* we perform the “infinitesimal coordinate transformation” ${\zeta }[h]$ as defined above to obtain a metric in Bondi gauge, still denoted by *h* but occasionally by ${h^{\mathrm{Bo}}}$. It still remains to take care of the boundary conditions. In particular, after the above, the asymptotic field $\overset{{ (0)}}{h}_{AB}(u,\cdot )$ will not necessarily vanish, regardless of whether or not $\overset{{ (0)}}{h}_{AB}(u,\cdot )$ was zero before the ${\zeta }[h]$-transformation. In order to remedy this we take the *u*-parameterised family of covector fields ${\xi }_B(u ,\cdot )$ to be any family of solutions, smooth in *u*, of the equations (cf., e.g., [10, Théorème 3.4])

3.37 $$\begin{aligned} \mathring{D}_A {\xi }_B + \mathring{D}_B {\xi }_A - \mathring{D}^C {\xi }_C {{\mathring{{\gamma }}}}_{AB} + \overset{{ (0)}}{\check{h}}_{AB}(u,\cdot )- \frac{1}{2} \overset{{ (0)}}{\check{h}}{}^{C}{}_{C}(u,\cdot ) {{\mathring{{\gamma }}}}_{AB} =0 \,, \end{aligned}$$

where

$$\begin{aligned} \overset{{ (0)}}{\check{h}}_{\mu \nu }{:}{=} \lim _{r\rightarrow \infty } r^{-2}h_{\mu \nu } \,. \end{aligned}$$

These solutions will be denoted by $\xi ^A[h]$.

It follows from the first line of (3.29) that the gauge-transformed fields $\overset{{ (0)}}{\check{h}}_{AB}(u,\cdot )$ will vanish.

Equation (3.37) determines ${\xi }_A[h](u,x^B)$ up to a *u*-dependent family of conformal Killing vectors of the round two-dimensional sphere; we will return to this freedom shortly.

We let ${\xi }^u[h]$ be a solution of (compare (3.27) and (3.36))

3.38 $$\begin{aligned} \partial _{{u}} {\xi }^{{u}}[h]({u},x^A)= & {} \left( {h^{\mathrm{reg}}}_{ur} + \frac{1}{2 }\epsilon N^2 {h^{\mathrm{reg}}}_{rr} + \frac{1}{2} \mathring{D}^A \xi _A [h] \right) \big |_{r=0} \nonumber \\= & {} \frac{1}{2} {h^{\mathrm{reg}}}_{tt}|_{r=0} +\frac{1}{4} {h^{\mathrm{reg}}}_{ij}|_{r=0}\nonumber \\&\times \left( \delta ^{ij} - 3 \frac{x^ix^j}{r^2}\right) + \frac{1}{2} \mathring{D}^A \xi _A[h] \,, \end{aligned}$$

with smooth initial data ${\xi }^u(u_0,\cdot )$ for some $u_0$. In vacuum (cf. (4.12) below) this leads to a gauge-transformed field for which

$$\begin{aligned} h_{ur} \equiv 0 \,. \end{aligned}$$

This procedure leads to a metric perturbation satisfying all Bondi gauge conditions together with

3.39 $$\begin{aligned} \overset{{ (0)}}{\check{h}}_{AB}\equiv 0 \equiv h_{ur} \,, \end{aligned}$$

where the second equality requires $T_{ur}\equiv 0$.

### Residual gauge

The freedom of choosing the vector field $\mathring{\zeta }$ of (3.13) describes the freedom to perform coordinate transformations preserving the Bondi form of the metric. These will be referred to as *residual gauge-transformations*. An example is provided by the vector field $\xi [h]$ just defined.

Under these $\mathring{\zeta }$-transformations, the linearised metric components acquire the following terms:

3.40 $$\begin{aligned} h_{uu} : \ {\mathcal {L}}_{\mathring{\zeta }} g_{uu}= & {} \mathring{\zeta }^r \epsilon \partial _r {N}^2 +2 \epsilon {N}^2 \partial _{{u}} \mathring{\zeta }^{{u}} -2 \partial _{{u}}\mathring{\zeta }^{{r}} \nonumber \\= & {} -(2+{\varDelta _{{{\mathring{{\gamma }}}}}}) \partial _{u} \xi ^{u} +{r}(\mathring{D}_{B} \partial _{{u}}{\xi }^{B}+\alpha ^{2} {\varDelta _{{{\mathring{{\gamma }}}}}}{\xi }^{{u}}) \nonumber \\&+\alpha ^{2}r^{2}(2\partial _{{u}}{\xi }^{{u}} - \mathring{D}_{B}{\xi }^{B}) \,, \end{aligned}$$

3.41 $$\begin{aligned} h_{ur}: \ {\mathcal {L}}_{\mathring{\zeta }} g_{ur}= & {} - \partial _{{u}} \mathring{\zeta }^{{u}}- \partial _{{r}} \mathring{\zeta }^{{r}} \nonumber \\= & {} - \partial _{{u}} {\xi }^{{u}} + \frac{ \mathring{D}_{B} {\xi }^{B} }{2} \,, \end{aligned}$$

3.42 $$\begin{aligned} h_{uA} : \ {\mathcal {L}}_{\mathring{\zeta }} g_{uA}= & {} {r}^2 {{\mathring{{\gamma }}}}_{AB}\partial _{{u}}{ \mathring{{\zeta }}^{B}} - \partial _{A} \mathring{\zeta }^{{r}} + \epsilon {N}^2 \partial _{A} \mathring{\zeta }^{{u}} \nonumber \\= & {} - \frac{1}{2}\partial _{A} \big [ \big ({\varDelta _{{{\mathring{{\gamma }}}}}}{\xi }^{{u}}+ 2 \xi ^{u}\big ) + {r }( \mathring{D}_{B} {\xi }^{B}-2\partial _{u} \xi ^{u}) \big ] \nonumber \\&+ r^{2}({{\mathring{{\gamma }}}}_{AB}\partial _{{u}} {\xi }^{B}+\alpha ^{2}\partial _{A}\xi ^{u}) \,, \end{aligned}$$

3.43 $$\begin{aligned} h_{AB} : \ {\mathcal {L}}_{\mathring{\zeta }} g_{AB}= & {} 2 {r}\mathring{\zeta }^{{r}} {{\mathring{{\gamma }}}}_{AB} + {r}^2 \mathring{{\mathcal {L}}}_{\mathring{\zeta }} {{\mathring{{\gamma }}}}_{AB} \nonumber \\= & {} {r}^2 ( \mathring{{\mathcal {L}}}_{{\xi }} {{\mathring{{\gamma }}}}_{AB} - {{\mathring{{\gamma }}}}_{AB} \mathring{D}_{C} {\xi }^{C} ) \nonumber \\&- {r}( 2 \mathring{D}_{A}\mathring{D}_{B} {\xi }^{{u}}- {{\mathring{{\gamma }}}}_{AB} {\varDelta _{{{\mathring{{\gamma }}}}}}{\xi }^{{u}} ) \,. \end{aligned}$$

The residual gauge transformations are thus defined by a *u*-parameterised family of vector fields ${\xi }^A(u,\cdot )$ on $S^2$ together with

3.44 $$\begin{aligned} \partial _{{u}} {\xi }^{{u}}({u},x^A) = \frac{ \mathring{D}_{B} {\xi }^{B}({u}, x^{A})}{2} \,, \end{aligned}$$

and (3.25). Explicitly:

3.45 $$\begin{aligned} \nonumber \mathring{\zeta }= & {} \left( \int \frac{ \mathring{D}_{B} {\xi }^{B}(u, x^{A})}{2} du + \mathring{{\xi }}{}^u(x^ A) \right) \partial _u\nonumber \\&+ \frac{1}{2} \left( {{\varDelta _{{{\mathring{{\gamma }}}}}}{\xi }^{{u}}} - {{r}\mathring{D}_{B} {\xi }^{B}} \right) \partial _r \nonumber \\&+ \left( {\xi }^B(u,x^A) - \frac{1}{{r}} \mathring{D}^B {\xi }^{{u}}({u},x^A) \right) \partial _B \,, \end{aligned}$$

with an arbitrary function $\mathring{{\xi }}^u(x^A)$.

#### Asymptotic symmetries

Unless explicitly indicated otherwise, in the remainder of this work we suppose that the metric has been transformed to Bondi form with $\overset{{ (0)}}{\check{h}}_{AB} \equiv 0$. The residual gauge transformation which preserve this condition will take the form (3.45) with, at each *u*, ${\xi }^B(u, x^A)\partial _B$ being a conformal Killing vector field of ${{\mathring{{\gamma }}}}$.

The conformal Killing vectors of $S^2$ are related to the Lorentz group, and the remaining freedom in ${\xi }^u$ corresponds to translations and supertranslations.

All these gauge transformations are interpreted as governing *asymptotic symmetries*, defined here as transformation which preserve the Bondi gauge as well as the asymptotic fall off condition for linearised fields.

In the asymptotically flat case (thus $\varLambda =0$) the fields ${\xi }^A$ become *u*-independent by (consistently) requiring in addition that $\overset{{ (0)}}{\check{h}}_{uA} \equiv 0$.

However, when $\varLambda >0$, the requirement $\overset{{ (0)}}{\check{h}}_{uA} \equiv 0$ is not consistent with (3.39) for general metric perturbations considered so far. Indeed, it follows from (3.42) that under the ${\xi }$-gauge transformations, the $r^2$-terms in $h_{uA}$ transform as

3.46 $$\begin{aligned} { \overset{{ (0)}}{\check{h}}_{uA}}({u},x^A)\mapsto & {} { \overset{{ (0)}}{\check{h}}_{uA}}({u},x^A) + {{\mathring{{\gamma }}}}_{AB}\partial _{{u}} {\xi }^{B} ({u},x^A)\nonumber \\&+ \alpha ^2 \partial _{A} {\xi }^{{u}}({u}, x^{A}) \,, \end{aligned}$$

keeping in mind that $\epsilon =1$ for large *r* While this shows that we can always choose $\partial _u{\xi }^B$ so that the gauge-transformed field ${ \overset{{ (0)}}{\check{h}}_{uB}}$ vanishes identically, such a choice will not be compatible with the requirement that $\overset{{ (0)}}{\check{h}}_{AB}\equiv 0$ in general when $\varLambda \ne 0$. Indeed, we have the transformation law

3.47 $$\begin{aligned} \overset{{ (0)}}{\check{h}}_{AB}(u,\cdot ) \mapsto \left( \mathring{D}_A {\xi }_B + \mathring{D}_B {\xi }_A - \mathring{D}^C {\xi }_C {{\mathring{{\gamma }}}}_{AB} + \overset{{ (0)}}{\check{h}}_{AB} \right) (u,\cdot ) . \end{aligned}$$

One could therefore choose ${\xi }^B$ so that

3.48 $$\begin{aligned} \overset{{ (0)}}{\check{h}}_{AB}(u_0,\cdot ) \equiv 0 \equiv \overset{{ (0)}}{\check{h}}_{uB}(u.\cdot ) \,, \end{aligned}$$

but there does not seem to be any reason why $\overset{{ (0)}}{\check{h}}_{AB}(u ,\cdot )$ should then be zero in general for $u\ne u_0$.

In the gauge (3.48) the canonical energy on $\mathscr {C}_{u,R}$ diverges as $R^3$ when *R* tends to infinity for $u\ne u_0$, while $R^2$ is replaced by *R* when (3.39) holds. This property makes the gauge $\overset{{ (0)}}{\check{h}}_{AB}\equiv 0$ more attractive from our perspective.

#### Rigid transport of sections of $\mathscr {I}^{+}$

When $\varLambda \ne 0$, a prescription to reduce the set of asymptotic symmetries has been presented in [12]. For the sake of completeness we reproduce the construction here.

The Hodge–Kodaira decomposition of one-forms on $S^2$ shows that there exist functions $\hat{\chi }(u)$ and $\check{\chi }(u)$ on $S^2$ such that

3.49 $$\begin{aligned} \overset{{ (0)}}{\check{h}}_{uA}({u},\cdot )=\mathring{D}_{A}\hat{\chi }(u)+{\varepsilon _{A}}^{B} \mathring{D}_{B}\check{\chi }(u) \,, \end{aligned}$$

where ${\varepsilon _{B}}^{C}$ is the two-dimensional Levi–Civita tensor. We can similarly write $\xi _B$ as

3.50 $$\begin{aligned} \xi _{B}(u,\cdot )=\mathring{D}_{B}\iota (u)+{\varepsilon _{B}}^{C} \mathring{D}_{C} \upsilon (u) \,, \end{aligned}$$

where the functions $\iota (u)$ and $\upsilon (u)$ are linear combinations of $\ell =1$ spherical harmonics (see Appendix A). Equation (3.46) can be rewritten as

3.51 $$\begin{aligned} \hat{\chi }(u)&\mapsto \hat{\chi }(u) + \partial _u \iota (u) + \alpha ^2 {\xi }^{{u}}({u}, \cdot ) \,, \end{aligned}$$

3.52 $$\begin{aligned} \check{\chi }(u)&\mapsto \check{\chi }(u) + \partial _u \upsilon (u) \,. \end{aligned}$$

Let $P_1$ denote the $L^2(S^2)$-orthogonal projection on the space of $\ell =1$ spherical harmonics. We can arrange that $P_1 \big (\check{\chi }\big )$ vanishes by solving the linear ODE

3.53 $$\begin{aligned} \partial _u \upsilon = - P_1 \big (\check{\chi }\big ) \,, \end{aligned}$$

which leaves the freedom of choosing $\upsilon (u_0)$.

Next, using (3.51) and (3.44) we obtain

3.54 $$\begin{aligned} \partial _u\hat{\chi }(u)&\mapsto \partial _u \hat{\chi }(u) + \partial ^2_u \iota (u) + \alpha ^2 \partial _u{\xi }^{{u}}({u}, \cdot ) \nonumber \\&= \partial _u \hat{\chi }(u) + \partial ^2_u \iota (u) - \alpha ^2 \iota (u) \,. \end{aligned}$$

We can arrange that $\partial _u \big (P_1(\hat{\chi })\big )$ vanishes by solving the equation

3.55 $$\begin{aligned} \partial ^2_u \iota - \alpha ^2 \iota = P_1 \big ( \partial _u \hat{\chi }\big ) \,. \end{aligned}$$

Equation (3.51) shows that $P_1\hat{\chi }$ will vanish if

3.56 $$\begin{aligned} \partial _u \iota (u_0) + P_1\big ( \hat{\chi }(u_0) + \alpha ^2 {\xi }^{{u}}({u}_0, \cdot ) \big )= 0 \,. \end{aligned}$$

There remains the freedom of choosing $\iota (u_0)$, with the solutions of the homogeneous equation (3.55) taking the form

3.57 $$\begin{aligned} \iota (u, \cdot ) = e^{\alpha u} \iota _+(\cdot ) + e^{-\alpha u} \iota _-(\cdot ) \,, \end{aligned}$$

where $\iota _\pm$ are linear combinations for $\ell =1$ spherical harmonics.

Summarising, we can achieve a *rigid transport* of the Bondi coordinates from one sphere to the other by requiring that the potentials $\hat{\chi }$ and $\check{\chi }$ of (3.50) satisfy

3.58 $$\begin{aligned} P_1(\hat{\chi })\equiv 0 \equiv P_1(\check{\chi }) \,. \end{aligned}$$

We will refer to (3.58) as the *rigid transport condition*.

Under the rigid transport conditions, we have the freedom of choosing $\big (\iota (u_0), \partial _u \iota (u_0), \upsilon (u_0)\big )$, which is related to the freedom of rotating and boosting the initial light cone $\mathscr {C}_{u_0}$, and of choosing ${\xi }^u(u_0,\cdot )$, which is the equivalent of the supertranslations that arise in the case $\varLambda =0$, subject to the constraint

3.59 $$\begin{aligned} \partial _u \iota (u_0) + \alpha ^2 P_1\big ( {\xi }^{{u}}({u}_0, \cdot ) \big )= 0 \,. \end{aligned}$$

After imposing (3.58), the residual gauge transformations which also preserve the rigid transport condition (3.58) take the form (3.45) with an arbitrary function $\mathring{{\xi }}^u(x^A)$, and where $\mathring{{\xi }}^B (u,x^A)\partial _B$ is the angular part of a Killing vector field of de Sitter spacetime as in (3.50), thus $\upsilon$ is a *u*-independent linear combination of $\ell =1$ spherical harmonics, the potential $\iota$ takes the form (3.57), with $\partial _u \iota (u_0)$ satisfying (3.59).

## The linearised Einstein equations

### Linearised metric perturbations in Bondi coordinates

Let $\mathscr {N}$ be a null hypersurface given by $u=\mathrm {const}$. We will use *Bondi-type coordinates* and a *Bondi parameterisation* of the metric on $\mathscr {N}$:

4.1 $$\begin{aligned} g_{\alpha \beta }dx^{\alpha }dx^{\beta }= & {} -\frac{V}{r}e^{2\beta } du^2-2 e^{2\beta }dudr \nonumber \\&+r^2{\gamma }_{AB}\Big (dx^A-U^Adu\Big )\Big (dx^B-U^Bdu\Big ) \, . \nonumber \\ \end{aligned}$$

Here it is also assumed that $\det \gamma _{AB}$ takes a canonical, *r*- and *u*-independent value, namely

4.2 $$\begin{aligned} \det \gamma = \sin ^2\theta \,. \end{aligned}$$

In spacetime dimension four, the Euler–Lagrange equations for the Lagrangian (2.107) with ${\mathring{\nabla }}$ replaced by $\nabla$ are

4.3 $$\begin{aligned}&\nabla ^{\mu } \nabla _{\alpha } \delta g_{\beta \mu } +\nabla ^{\mu } \nabla _{\beta } \delta g_{\alpha \mu } +g_{\alpha \beta }\nabla _{\kappa }\nabla ^{\kappa } \delta {g_{\lambda }}^{\lambda } -\nabla _{\mu } \nabla ^{\mu } \delta g_{ \alpha \beta }\nonumber \\&\quad -g_{\alpha \beta } \nabla ^{\kappa } \nabla ^{\lambda } \delta g_{\kappa \lambda } -\nabla _{\alpha } \nabla _{\beta } \delta {g^{\kappa }}_{\kappa } = 2 \varLambda \left( \delta g_{\alpha \beta } - \frac{1}{2} \delta g^\kappa {}_\kappa g_{\alpha \beta } \right) .\nonumber \\ \end{aligned}$$

Assuming $\delta {g^{\kappa }}_{\kappa }=0$, which is the case in the Bondi gauge, we obtain

4.4 $$\begin{aligned} \mathscr {E}_{\alpha \beta }:= & {} \nabla ^{\mu } \nabla _{\alpha } \delta g_{\beta \mu } +\nabla ^{\mu } \nabla _{\beta } \delta g_{\alpha \mu } -\nabla _{\mu } \nabla ^{\mu } \delta g_{ \alpha \beta }\nonumber \\&-g_{\alpha \beta } \nabla ^{\kappa } \nabla ^{\lambda } \delta g_{\kappa \lambda } - 2 \varLambda \delta g_{\alpha \beta } =0 \, . \end{aligned}$$

Taking a trace we find

4.5 $$\begin{aligned} \nabla ^{\kappa } \nabla ^{\lambda } \delta g_{\kappa \lambda } =0 \, , \end{aligned}$$

which simplifies (4.4) further to

4.6 $$\begin{aligned} \nabla ^{\mu } \nabla _{\alpha } \delta g_{\beta \mu } +\nabla ^{\mu } \nabla _{\beta } \delta g_{\alpha \mu } -\nabla _{\mu } \nabla ^{\mu } \delta g_{ \alpha \beta } - 2 \varLambda \delta g_{\alpha \beta } =0 \, . \end{aligned}$$

These equations are still unpleasant enough so that it appears simpler to instead linearise the equations as written down in [46], and we will do so. Nevertheless, to be on the safe side, we have checked, using Maple, that the set of equations ${\mathscr {E}^{u}}_{r}={\mathscr {E}^{u}}_{u}={\mathscr {E}^{u}}_{A}=0$ is equivalent to the linearised equations (4.12), (4.14) and (4.32) below, obtained from the equations in [46].

Our asymptotic conditions on the linearised perturbations of the metric will be modelled on the asymptotic behaviour of the full solutions of the Einstein vacuum field equations with positive cosmological constant and with smooth initial Cauchy data on $S^3$, as constructed by Friedrich in [29]. The resulting spacetimes have a smooth conformal completion with a (necessarily spacelike) boundary at (timelike) infinity $\mathscr {I}^{+}$ (denoted by $I^+$ by some authors). It is shown in [13, Section 2.1] that, given a foliation of $\mathscr {I}^{+}$ by a function $y^{ 0}$ (in our case, this foliation will be provided by the intersections of a family of null light cones emanating from a world-line in spacetime), there exists a neighborhood of $\mathscr {I}^{+}$ on which the metric takes the Bondi form (4.1), where $u_{}|_{\mathscr {I}^{+}} = y^{ 0}$. Now, Bondi et al. consider the case $\varLambda =0$ and assume

4.7 $$\begin{aligned} \lim _{r_{{}} \rightarrow \infty } U^A_{}= 0 \,, \quad \lim _{r_{{}} \rightarrow \infty } \beta = 0 \,, \quad \lim _{r_{{}} \rightarrow \infty } \left( r_{{}}^{-2} {g}^{}_{AB} \right) = {\mathring{{\gamma }}}_{AB} \,, \end{aligned}$$

where ${\mathring{{\gamma }}}^{}_{AB}$ is the standard metric on $S^2$. It follows e.g. from [13, Section 2.1] that the last equation in (4.7) is justified under the hypothesis of existence of a smooth conformal completion at infinity regardless of the value of $\varLambda$. However, it is not clear at all whether the first two equations (4.7) can be assumed to hold *for all retarded times* in general: When $\varLambda <0$ this is part of asymptotic conditions which one is free to impose, and which are usually imposed in this context, but which one might *not* want to impose in *some* situations. When $\varLambda =0$ these conditions can be realised by choosing the function $y^{0}$ suitably. However, when $\varLambda >0$ there is little doubt that all three conditions in (4.7) can be simultaneously satisfied for all retarded times by a *restricted class of metrics* only. In the linearised theory this will be clear from the calculations that follow.

We have mentioned above the results of Friedrich on the spacelike relativistic Cauchy problem, as they guarantee existence of a large class of vacuum spacetimes with a positive cosmological constant, near the de Sitter or Anti de Sitter spacetime, with a smooth conformal completion at Scri. As such, in our context it is more natural to think of the characteristic rather than the spacelike Cauchy problem (cf., e.g., [18]). In this context, in the linearised theory we are free to prescribe arbitrarily the angular part $h_{AB} dx^A dx^B$ of the linearised metric perturbation on the light cone, with the remaining fields, and their asymptotics, determined by these free data and the residual gauge conditions to which we return in Sect. 3.1. This follows quite generally from the analysis in e.g. [18], but can also be deduced directly from the considerations that we are about to present. Friedrich’s results just mentioned guarantee, e.g. by taking data induced on light cones from his solutions, that there exists a large class of free data $h_{AB} dx^A dx^B$ on the initial light cone with an evolution which is smoothly conformally compactifiable at $\mathscr {I}^{+}$, and we restrict our attention to such data.

Writing interchangeably

$$\begin{aligned} h_{\mu \nu } \text{ for } \delta g_{\mu \nu }, \text{ and } \check{h}_{\mu \nu } \text{ for } r^{-2}h_{\mu \nu }, \end{aligned}$$

we thus assume the following large-*r* expansion

4.8 $$\begin{aligned} h_{AB}= & {} r^2 \underbrace{\overset{{ (2)}}{h}_{AB}}_0 + r \overset{{ (1)}}{h}_{AB} + \overset{{ (0)}}{h}_{AB} + r^{-1} \overset{{ (-1)}}{h}_{AB} + o(r^{-1}) \big ) \nonumber \\\equiv & {} r^2 \big ( \underbrace{\overset{{ (0)}}{\check{h}}_{AB}}_0 + r^{-1} \overset{{ (-1)}}{\check{h}}_{AB} + r^{-2} \overset{{ (-2)}}{\check{h}}_{AB} + r^{-3} \overset{{ (-3)}}{\check{h}}_{AB}\nonumber \\&+ o(r^{-3}) \big ) \,, \end{aligned}$$

where the expansion tensors $\overset{{ (1)}}{h}_{AB}\equiv \overset{{ (-1)}}{\check{h}}_{AB}$, etc., are independent of *r*.

A comment on our hypothesis that $\overset{{ (0)}}{\check{h}}_{AB}\equiv 0$ is in order. As discussed in detail in Sect. 3.1, after transforming the metric perturbation to the Bondi form we can always use the remaining coordinate freedom to achieve $\overset{{ (0)}}{\check{h}}_{AB}=0$. An alternative possibility is $\overset{{ (0)}}{\check{h}}_{uA}=0$. A key fact is that $\overset{{ (0)}}{\check{h}}_{AB}=0$ and $\overset{{ (0)}}{\check{h}}_{uA}=0$ cannot be achieved simultaneously in general. As already mentioned, the condition $\overset{{ (0)}}{\check{h}}_{uA}=0$ (in which case $\overset{{ (0)}}{\check{h}}_{AB}\ne 0$ in general) leads to energy integrals on balls of radius *R* which diverge as $R^3$, and therefore we have opted for $\overset{{ (0)}}{\check{h}}_{AB}=0$ which leads to a slower divergence.

Smooth compactifiability of the solution guarantees that there will be no logarithmic terms in the asymptotic expansion, which in turn requires

4.9 $$\begin{aligned} \overset{{ (-2)}}{\check{h}}_{AB} \equiv 0 \,, \end{aligned}$$

as follows from our calculations below (compare [13]). However, we will not assume (4.9) at this stage, to be able to track down the role of this term in the equations that follow.

We will derive precise information on the asymptotic behaviour of the remaining linearised fields using the characteristic constraint equations in Bondi coordinates, which read [46]

4.10 $$\begin{aligned}&\partial _r \beta = \frac{r}{16}{\gamma }^{AC}{\gamma }^{BD} (\partial _r {\gamma }_{AB})(\partial _r {\gamma }_{CD}) + 2\pi r T_{rr} \,, \end{aligned}$$

4.11 $$\begin{aligned}&\partial _r \left[ r^4 e^{-2\beta }{\gamma }_{AB}(\partial _r U^B)\right] = 2r^4\partial _r \Big (\frac{1}{r^2}{ D}_A\beta \Big ) \nonumber \\&\quad -r^2{\gamma }^{EF} { D}_E (\partial _r {\gamma }_{AF}) +16\pi r^2 T_{rA} \,, \end{aligned}$$

as well as (4.31) below. Here ${ D}_A$ is the covariant derivative of the 2-metric ${\gamma }_{AB}$.

#### $h_{ur}$

Since the right-hand side of (4.10) is quadratic in $\partial _r\gamma _{AB}$, assuming a vacuum spacetime everywhere, after linearising we find

4.12 $$\begin{aligned} \partial _r \delta \beta =0 \quad \Longleftrightarrow \quad \delta \beta = \delta \beta (u,x^A) \,. \end{aligned}$$

Hence we can use the ${\xi }$-gauge transformation (3.41) to obtain

4.13 $$\begin{aligned} \delta \beta \equiv 0 \quad \Longleftrightarrow \quad \delta g_{ur } \equiv 0 \,. \end{aligned}$$

#### $h_{uA}$

The linearisation of (4.11) at the de Sitter metric gives now, in vacuum,

4.14 $$\begin{aligned} \partial _r \left[ r^4 \partial _r(r^{-2} \delta g_{uA})\right] = r^2 \mathring{D}_E \left( {\mathring{{\gamma }}}^{EF}\partial _r \left( r^{-2}\delta g_{AF}\right) \right) \,. \end{aligned}$$

Let $\psi _A$ denote the right-hand side of the last equation,

4.15 $$\begin{aligned} \psi _A{:}{=} r^2 \mathring{D}_E \left( {\mathring{{\gamma }}}^{EF}\partial _r \left( r^{-2}\delta g_{AF}\right) \right) \,. \end{aligned}$$

Equation (4.8) gives, for large *r*,

4.16 $$\begin{aligned} \psi _A = - \mathring{D}^B \overset{{ (-1)}}{\check{h}}_{AB} -2 r^{-1}\mathring{D}^B \overset{{ (-2)}}{\check{h}}_{AB} -3 r^{-2} \mathring{D}^B \overset{{ (-3)}}{\check{h}}_{AB} + {o(r^{-2}) } \,. \end{aligned}$$

while for small *r* we have, from (3.29),

4.17 $$\begin{aligned} \psi _A = \mathring{D}^B ( 2 \mathring{D}_{A}\mathring{D}_{B} {\xi }^{{u}} - {\varDelta _{{{\mathring{{\gamma }}}}}}{\xi }^{{u}} {{\mathring{{\gamma }}}}_{AB} ) + O(r ) \,. \end{aligned}$$

Here ${\xi }$ is the gauge field of (3.9), cf. (3.1).

As $\psi _A$ tends to a non-zero covector field as *r* goes to infinity in general, it turns out to be convenient to write the general solution of (4.14) as

4.18 $$\begin{aligned} r^{-2} \delta g_{u A}= & {} {\mu }_A(u,x^B) + \frac{{\lambda }_A(u,x^B)}{r^3 }\nonumber \\&+ \int _{1}^{r}\left[ \frac{1}{\rho ^{4}} \int _{0}^{\rho } \psi _{A}(s) d s\right] d \rho \nonumber \\= & {} {\mu }_A(u,x^B) + \frac{{\lambda }_A(u,x^B)}{r^3 } \nonumber \\&+ \underbrace{ \int _{1}^{\infty }\left[ \frac{1}{\rho ^{4}} \int _{0}^{\rho } \psi _{A}(s) d s\right] d \rho }_{{=}{:}\mathring{{\mu }}_A} \nonumber \\&-\int _{r}^{\infty } \left[ \frac{1}{\rho ^{4}} \int _{0}^{\rho } \psi _{A}(s) d s\right] d \rho \,, \end{aligned}$$

with fields ${\mu }_A$ and ${\lambda }_A$ depending upon the arguments indicated. Here $\psi _A(s)$ stands for $\psi _A(u,s,x^A)$. It follows from (3.42) that the requirement, that $\delta g$ is obtained by an infinitesimal coordinate transformation from a metric perturbation which is smooth near the vertex of the cone, enforces boundedness of $r^{-2} \delta g_{u A}$. This, together with (4.17) shows that

4.19 $$\begin{aligned} {\lambda }_A \equiv 0 \,. \end{aligned}$$

The field ${\mu }_A$ has a gauge character and can be determined by imposing convenient conditions at infinity, as follows from the results in Sect. 3.2.

We have for large $\rho$

4.20 $$\begin{aligned} \int _{0}^{\rho } \psi _{A}(s) d s= & {} - \mathring{D}^B \overset{{ (-1)}}{\check{h}}_{AB} \, \rho -2 \mathring{D}^B \overset{{ (-2)}}{\check{h}}_{AB} \, \ln \rho -3 \mathring{{\lambda }}_A \nonumber \\&+3 \rho ^{-1} \mathring{D}^B \overset{{ (-3)}}{\check{h}}_{AB} + { o(\rho ^{-1}) } \,, \end{aligned}$$

where

4.21 $$\begin{aligned} \mathring{{\lambda }}_A:= & {} - \frac{1}{3} \lim _{r\rightarrow \infty } \bigg ( \int _{0}^{r} \psi _{A}(s) d s + \mathring{D}^B \overset{{ (-1)}}{\check{h}}_{AB} \, r \nonumber \\&+2 \mathring{D}^B \overset{{ (-2)}}{\check{h}}_{AB}\, \ln r \bigg ) \,. \end{aligned}$$

This leads to the following expansion, for large *r*,

4.22 $$\begin{aligned}&-\int _{r}^{\infty } \left[ \frac{1}{\rho ^{4}} \int _{0}^{\rho } \psi _{A}(s) d s\right] d \rho \nonumber \\&\quad = \frac{1}{2} \mathring{D}^B \overset{{ (-1)}}{\check{h}}_{AB} \, r^{-2} + \frac{2}{9} \mathring{D}^B \overset{{ (-2)}}{\check{h}}_{AB} \, (3 \ln r + 1) r^{-3} \nonumber \\&\qquad + \mathring{{\lambda }}_A r^{-3} - \frac{3}{4} \mathring{D}^B \overset{{ (-3)}}{\check{h}}_{AB} r^{-4} + { o(r^{-4}) } \,, \end{aligned}$$

resulting in^2^

4.23 $$\begin{aligned} {\check{h}_{uA}} {:}{=}&r^{-2} \delta g_{u A} = {\mu }_A + \mathring{{\mu }}_A + \frac{1}{2} \mathring{D}^B \overset{{ (-1)}}{\check{h}}_{AB} \, r^{-2} \nonumber \\&+ \left( \mathring{{\lambda }}_A + \frac{2}{9} \mathring{D}^B \overset{{ (-2)}}{\check{h}}_{AB} \, (3 \ln r + 1) \right) r^{-3} \nonumber \\&- \frac{3}{4} \mathring{D}^B \overset{{ (-3)}}{\check{h}}_{AB} \, r^{-4} + { o(r^{-4}) } \,. \end{aligned}$$

#### $\partial _u h_{AB}$

We continue with an analysis of the asymptotics of $\partial _u h_{AB}$, which can be determined from [46, Equation (32)]. Denoting the traceless symmetric part of a tensor on the sphere by

$$\begin{aligned} TS[\cdot ] \,, \end{aligned}$$

we have

4.24 $$\begin{aligned}&TS\Bigg [ r\partial _r [r (\partial _u {\gamma }_{AB})] - \frac{1}{2} \partial _r[ rV (\partial _r {\gamma }_{AB})] -2e^{\beta } { D}_A { D}_B e^\beta \nonumber \\&\quad + {\gamma }_{CA} { D}_B[ \partial _r (r^2U^C) ] - \frac{1}{2} r^4 e^{-2\beta }{\gamma }_{AC}{\gamma }_{BD} (\partial _r U^C) (\partial _r U^D) \nonumber \\&\quad + \frac{r^2}{2} (\partial _r {\gamma }_{AB}) ({ D}_C U^C ) +r^2 U^C { D}_C (\partial _r {\gamma }_{AB}) \nonumber \\&\quad - r^2 (\partial _r {\gamma }_{AC}) {\gamma }_{BE} ({ D}^C U^E -{ D}^E U^C) -8\pi e^{2\beta }T_{AB} \Bigg ] =0.\nonumber \\ \end{aligned}$$

Linearising around the de Sitter background one obtains

4.25 $$\begin{aligned}&r\partial _r [r (\partial _u \check{h}_{AB})] + \frac{\epsilon }{2} \partial _r[ r^2 {N}^2 (\partial _r \check{h}_{AB})] - TS \big [ \mathring{D}_A\big ( \partial _r (r^2\check{h}_{uB}) \big )\big ] \nonumber \\&\quad = 8\pi TS[ \delta T_{AB} ] \,. \end{aligned}$$

Integrating in vacuum, we find for large *r*

4.26 $$\begin{aligned} \partial _u \check{h}_{AB} (r,\cdot )= & {} - \frac{1}{r} \int _{0}^r \frac{1}{s} \left( \frac{\epsilon }{2} \partial _r[r^2 {N}^2 (\partial _r \check{h}_{AB})] \right. \nonumber \\&\quad \left. - TS \big [ \mathring{D}_A\big ( \partial _r (r^2\check{h}_{uB}) \big )\big ] \right) (s,\cdot ) ds \nonumber \\= & {} \partial _u \overset{{ (0)}}{\check{h}}_{AB}(\cdot ) + \frac{ \partial _u \overset{{ (-1)}}{\check{h}}_{AB}(\cdot ) }{r } \nonumber \\&\quad + \frac{ \partial _u \overset{{ (-3)}}{\check{h}}_{AB}(\cdot ) }{r^3} + o(r^{-3}) \,, \end{aligned}$$

where

4.27 $$\begin{aligned}&\partial _u \overset{{ (0)}}{\check{h}}_{AB}(\cdot )= \alpha ^2 \overset{{ (-1)}}{\check{h}}_{AB} + \big ( \mathring{D}_A { \overset{{ (0)}}{\check{h}}_{uB}} + \mathring{D}_B { \overset{{ (0)}}{\check{h}}_{uA}}\nonumber \\&\quad - \mathring{D}^C \overset{{ (0)}}{\check{h}}_{u C} {{\mathring{{\gamma }}}}_{AB} \big ) \,, \end{aligned}$$

4.28 $$\begin{aligned}&\partial _u \overset{{ (-1)}}{\check{h}}_{AB}(\cdot ) = - \lim _{R\rightarrow \infty }\bigg ( \int _{0}^{R}\frac{1}{s} \Big ( \frac{\epsilon }{2} \partial _r[ r^2{N}^2 (\partial _r \check{h}_{AB})] \nonumber \\&\quad - TS \big [ \mathring{D}_A\big ( \partial _r (r^2\check{h}_{uB}) \big )\big ] \Big )(s,\cdot ) ds + \partial _u \overset{{ (0)}}{\check{h}}_{AB}(\cdot ) R \bigg ) \,, \end{aligned}$$

4.29 $$\begin{aligned}&\partial _u \overset{{ (-3)}}{\check{h}}_{\!}(\cdot ) = \alpha ^2 \overset{{ (-4)}}{\check{h}}_{AB} + \big ( \mathring{D}_A { \overset{{ (-3)}}{\check{h}}_{uB}} + \mathring{D}_B { \overset{{ (-3)}}{\check{h}}_{uA}}\nonumber \\&\quad - \mathring{D}^C \overset{{ (-3)}}{\check{h}}_{u C} {{\mathring{{\gamma }}}}_{AB} \big ), \,\,\,\, \end{aligned}$$

and note that the limit in (4.284.29) exists and is finite under the current conditions. Also note that (4.26) has no $r^{-2}$ terms, which shows that the expansion coefficients $\overset{{ (-2)}}{\check{h}}_{AB}$ are constants of motion.

The choice of asymptotic gauge $\overset{{ (0)}}{\check{h}}_{AB} \equiv 0$ implies $\partial _u \overset{{ (0)}}{\check{h}}_{AB}\equiv 0$, equivalently

4.30 $$\begin{aligned} \mathring{D}_A { \overset{{ (0)}}{\check{h}}_{uB}} + \mathring{D}_B { \overset{{ (0)}}{\check{h}}_{uA}} - \mathring{D}_C \overset{{ (0)}}{\check{h}}_u{}^C {{\mathring{{\gamma }}}}_{AB} = - \alpha ^2 \overset{{ (-1)}}{\check{h}}_{AB} \,. \end{aligned}$$

#### $h_{uu}$

The *V* function occurring in the Bondi form of the metric solves the equation [46]

4.31 $$\begin{aligned}&2 e^{-2\beta } \partial _r V = \mathscr {R} -2{\gamma }^{AB} \Big [{ D}_A { D}_B \beta + ({ D}_A\beta ) ({ D}_B \beta )\Big ]\nonumber \\&\quad +\frac{e^{-2\beta }}{r^2 }{ D}_A \Big [ \partial _r (r^4U^A)\Big ] -\frac{1}{2}r^4 e^{-4\beta }{\gamma }_{AB}(\partial _r U^A)(\partial _r U^B) \nonumber \\&\quad - 8\pi \Big [ {\gamma }^{AB}T_{AB}-r^2 T^\alpha {}_\alpha \Big ] - {2 \varLambda r^2}\,, \end{aligned}$$

where $\mathscr {R}$ is the Ricci scalar of the conformal two-metric ${\gamma }_{AB}$. Assuming $\delta \beta \equiv 0$, the linearised version of (4.31) reads

4.32 $$\begin{aligned} 2 \partial _r \delta V = \delta \mathscr {R} - \frac{1}{r^2 }\mathring{D}^A \Big [ \partial _r (r^4\check{h}_{uA})\Big ] + 8\pi \delta \Big [ {\gamma }^{AB}T_{AB}-r^2 T^{a}_{a}\Big ] \,. \end{aligned}$$

Let $\mathring{R}_{AB}$ denote the Ricci tensor of the metric ${{\mathring{{\gamma }}}}_{AB}$. As $h_{AB}$ is ${{\mathring{{\gamma }}}}$-traceless we have

4.33 $$\begin{aligned} r^2\delta \mathscr {R}= & {} -\mathring{D}^A\mathring{D}_A( {{\mathring{{\gamma }}}}^{BC }h_{BC})+\mathring{D}^A\mathring{D}^B h_{AB}-\mathring{R}^{AB}h_{AB} \nonumber \\= & {} \mathring{D}^A\mathring{D}^B h_{AB} \,. \end{aligned}$$

This leads to, in vacuum,

4.34 $$\begin{aligned} 2 \partial _r \delta V = \mathring{D}^A \left( \mathring{D}^B \check{h}_{AB} -\frac{1}{r^2 } \big [ \partial _r (r^4\check{h}_{uA })\big ] \right) . \end{aligned}$$

It follows from (4.23) that the $r^{-1}$ terms in the large-*r* expansion of the right-hand side of (4.34) cancel out, and that for large *r* we have

4.35 $$\begin{aligned} 2 \partial _r \delta V = \mathring{D}^A \big ( -4 r \overset{{ (0)}}{\check{h}}_{uA } - r^{-2} \overset{{ (-3)}}{\check{h}}_{uA } + o (r^{-2}) \big ) \,. \end{aligned}$$

Hence

4.36 $$\begin{aligned} \delta V= & {} \frac{1}{2} \mathring{D}^A \left( \int _0^r \left( \mathring{D}^B \check{h}_{AB} -\frac{1}{r^2 } \big [ \partial _r (r^4\check{h}_{uA })\big ] \right) \big |_{r=\rho } d\rho \right) \nonumber \\= & {} -\mathring{D}^{A} \left( r^2 \overset{{ (0)}}{\check{h}}_{uA } - \nu _A - \frac{1}{2 r} \overset{{ (-3)}}{\check{h}}_{uA } + o(1/r) \right) \end{aligned}$$

as *r* goes to infinity, where we used that $\delta V|_{r=0}=0$ vanishes when transforming to Bondi coordinates a field which was originally smooth near the tip of the light cone; compare (3.40). Here $\nu _A= \nu _A(u,x^B)$ equals

4.37 $$\begin{aligned} \nu _A= & {} \lim _{r\rightarrow \infty } \left( \frac{1}{2} \int _0^r \left( \mathring{D}^B \check{h}_{AB} -\frac{1}{r^2 } \big [ \partial _r (r^4\check{h}_{uA })\big ] \right) \big |_{r=\rho } d\rho \right. \nonumber \\&\left. + r^2 \overset{{ (0)}}{\check{h}}_{uA }\right) . \end{aligned}$$

We note that the individual terms in the integrands of (4.36) and (4.37) have, for small *r*, potentially dangerous 1/*r* terms which could lead to a logarithmic divergence of the integral near $r=0$. However, these terms have to cancel out for fields obtained by a coordinate transformation from metric perturbations which are smooth near the vertex. This can be seen by a direct calculation from (3.42) and (3.43). A simpler argument is to notice that a pure gauge field satisfies the linearised field equations, and that there are no logarithmic terms in the pure gauge fields (3.40)–(3.43).

#### The remaining Einstein equations

The remaining Einstein equations are irrelevant for our analysis in this paper, in the following sense (see [46]):

1. The *uu* part of the Einstein equations is an equation involving $\partial _u V$, which did not occur in the equations above and therefore cannot put further constraints on the expansion coefficients so far.
2. Similarly for the *uA* part of the Einstein equations, which involves $\partial _u U^A$.
3. The trace part of the angular part of the Einstein equations is automatically satisfied in Bondi coordinates once the remaining equations are satisfied.

We present detailed derivations for completeness.

The equation $\mathscr {E}_{u u}=0$ reads

4.38 $$\begin{aligned}&\frac{1}{ r^2}\bigg [2\left( \partial _{u} +(\alpha ^2 r^2-1) \partial _{r} -\frac{1}{r} \right) \mathring{D}^{A} {h}_{u A} - \mathring{D}^{A} \mathring{D}_{A} {h}_{u u} \nonumber \\&\quad -(\alpha ^2 r^2-1) \bigg (\frac{\mathring{D}^{A} \mathring{D}^{B} {h}_{A B}}{r^2}\bigg ) -2 r \partial _{u} {h}_{u u} \nonumber \\&\quad -2 (\alpha ^2 r^2-1) \partial _{r}(r {h}_{u u}) \bigg ] =0. \nonumber \\ \end{aligned}$$

We insert the asymptotic expansion of $h_{\mu \nu }$ into (4.38), obtaining

4.39 $$\begin{aligned}&4\alpha ^{2}r\big (\mathring{D}^{A}\overset{{ (2)}}{h}_{uA}-\overset{{ (1)}}{h}_{uu}\big ) + 2\partial _{u}\big (\mathring{D}^{A}\overset{{ (2)}}{h}_{uA}-\overset{{ (1)}}{h}_{uu}\big ) \nonumber \\&\quad +\frac{1}{r} \big ( 4 \overset{{ (1)}}{h}_{uu}- 6 \mathring{D}^{A}\overset{{ (2)}}{h}_{u A}-{\varDelta _{{{\mathring{{\gamma }}}}}}\overset{{ (1)}}{h}_{uu} - \alpha ^2 D^{A} D^{B} \overset{{ (1)}}{h}_{A B} \big ) \nonumber \\&\frac{2}{r^2} \big ( \partial _{u} \mathring{D}^{A} \overset{{ (0)}}{h}_{u A} -\alpha ^2 \mathring{D}^{A} \overset{{ (-1)}}{h}_{u A} - \partial _{u} \overset{{ (-1)}}{h}_{u u}+ \alpha ^2 \overset{{ (-2)}}{h}_{u u} \big )\nonumber \\&\quad = o \Big (\frac{1}{r^2} \Big ) \, . \end{aligned}$$

Hence

4.40 $$\begin{aligned}&\alpha ^{2} \big (\mathring{D}^{A}\overset{{ (2)}}{h}_{uA}-\overset{{ (1)}}{h}_{uu}\big ) = 0 \,, \end{aligned}$$

4.41 $$\begin{aligned}&\partial _{u}\big (\mathring{D}^{A}\overset{{ (2)}}{h}_{uA}-\overset{{ (1)}}{h}_{uu}\big ) = 0 \,, \end{aligned}$$

4.42 $$\begin{aligned}&4 \overset{{ (1)}}{h}_{uu}- 6 \mathring{D}^{A}\overset{{ (2)}}{h}_{u A}-{\varDelta _{{{\mathring{{\gamma }}}}}}\overset{{ (1)}}{h}_{uu} - \alpha ^2 D^{A} D^{B} \overset{{ (1)}}{h}_{A B} = 0 \,, \end{aligned}$$

4.43 $$\begin{aligned}&\partial _{u} \mathring{D}^{A} \overset{{ (0)}}{h}_{u A} -\alpha ^2 \mathring{D}^{A} \overset{{ (-1)}}{h}_{u A} - \partial _{u} \overset{{ (-1)}}{h}_{u u}+ \alpha ^2 \overset{{ (-2)}}{h}_{u u}= 0, \nonumber \\ \end{aligned}$$

and note that (4.40) implies (4.41) only if $\alpha \ne 0$; however, with our boundary conditions, the latter is trivially satisfied when $\alpha$ vanishes. Now, (4.36) gives

4.44 $$\begin{aligned} \overset{{ (-2)}}{h}_{u u} = - \frac{1}{2} \mathring{D}^{A} \overset{{ (-1)}}{h}_{u A} \, , \end{aligned}$$

while from (4.23) we obtain

4.45 $$\begin{aligned} \mathring{D}^{A} \overset{{ (0)}}{h}_{u A}=\frac{1}{2} \mathring{D}^{A} \mathring{D}^{B} \overset{{ (1)}}{h}_{A B} \,. \end{aligned}$$

Substituting (4.44) and (4.45) into (4.43) we are led to

4.46 $$\begin{aligned} \partial _{u} \overset{{ (-1)}}{h}_{u u}= \frac{1}{2} \partial _{u} \mathring{D}^{A} \mathring{D}^{B} \overset{{ (1)}}{h}_{A B} + 3\alpha ^2 \overset{{ (-2)}}{h}_{u u} \end{aligned}$$

Suppose, first, that $\alpha \ne 0$. Inserting (4.40) into (4.42) gives

4.47 $$\begin{aligned} \mathring{D}^{A} \mathring{D}^{B} \overset{{ (1)}}{h}_{A B}=- \frac{1}{\alpha ^2} \big ({\varDelta _{{{\mathring{{\gamma }}}}}}\overset{{ (1)}}{h}_{uu} +2 \overset{{ (1)}}{h}_{uu}\big ) \end{aligned}$$

Combining (4.46) and (4.47) leads to

4.48 $$\begin{aligned} \displaystyle \partial _{u} {\overset{{ (-1)}}{h}}_{u u}= & {} - \frac{1}{ 2 \alpha ^2 } ({\varDelta _{{{\mathring{{\gamma }}}}}}+ 2) \partial _u \overset{{ (1)}}{h}_{uu} + 3 \alpha ^2 {\overset{{ (-2)}}{h}}_{uu} \nonumber \\= & {} \frac{1}{2} \mathring{D}^{A} \mathring{D}^{B} \partial _{u} \overset{{ (1)}}{h}_{AB} + 3 \alpha ^2 {\overset{{ (-2)}}{h}}_{uu} \,. \end{aligned}$$

Similar manipulations show that (4.48) remains valid for $\alpha =0$:

4.49 $$\begin{aligned} \displaystyle \partial _{u} {\overset{{ (-1)}}{h}}_{u u}= & {} \frac{1}{2} \mathring{D}^{A} \mathring{D}^{B} \partial _{u} {\overset{{ (1)}}{h}}_{AB} \,, \end{aligned}$$

which is the linearisation of the usual equation for the time-evolution of the mass aspect function (cf., e.g. [15, Equation (5.102)]).

We continue with an analysis of the equations $\mathscr {E}_{u A}=0$, which read

4.50 $$\begin{aligned} 0= & {} \frac{1}{ r^2} \Bigg [ \mathring{D}^{B}\mathring{D}_{A}{h}_{uB} -\mathring{D}^{B}\mathring{D}_{B}{h}_{uA} +\partial _{u} \mathring{D}^{B}{h}_{A B} \nonumber \\&{-}r^{2}\left( \left( 1-r^{2}\alpha ^{2}\right) \partial _{r}^{2}h_{uA} + 2\alpha ^{2}h_{uA}\nonumber \right. \\&\left. -r^{2}\partial _{r}\partial _{u}\left( \frac{h_{uA}}{r^{2}}\right) + \partial _{r}\mathring{D}_{A}{h}_{uu} \right) \Bigg ] \,. \end{aligned}$$

An asymptotic expansion of the above equation takes the form

4.51 $$\begin{aligned} \mathscr {E}_{uA}= & {} \mathring{D}^{B}\mathring{D}_{A} \overset{{ (2)}}{h}_{uB} -\mathring{D}^{B}\mathring{D}_{B} \overset{{ (2)}}{h}_{uA} - 2 \overset{{ (2)}}{h}_{uA} -2\alpha ^{2} \overset{{ (0)}}{h}_{uA} \nonumber \\&-\mathring{D}_{A}\overset{{ (1)}}{h}_{uu} + \frac{1}{r}\partial _{u}\big (\mathring{D}^{B} \overset{{ (1)}}{h}_{AB} -2 \overset{{ (0)}}{h}_{uA} \big ) \nonumber \\&+\frac{1}{r^2} \big ( \mathring{D}^{B} \mathring{D}_{A} \overset{{ (0)}}{h}_{u B} -\mathring{D}^{B} \mathring{D}_{B} \overset{{ (0)}}{h}_{u A} - 3 \partial _{u} \overset{{ (-1)}}{h}_{u A} \nonumber \\&+4 \alpha ^2 \overset{{ (-2)}}{h}_{u A} +\mathring{D}_{A} \overset{{ (-1)}}{h}_{u u} \big )+ o\left( r^{-2}\right) \,. \end{aligned}$$

Hence

4.52 $$\begin{aligned}&\mathring{D}^{B}\mathring{D}_{A} \overset{{ (2)}}{h}_{uB} -\mathring{D}^{B}\mathring{D}_{B} \overset{{ (2)}}{h}_{uA} - 2 \overset{{ (2)}}{h}_{uA} -2\alpha ^{2} \overset{{ (0)}}{h}_{uA} -\mathring{D}_{A}\overset{{ (1)}}{h}_{uu}\nonumber \\&\quad = 0 \, , \end{aligned}$$

4.53 $$\begin{aligned}&\partial _{u}\big (\mathring{D}^{B} \overset{{ (1)}}{h}_{AB} -2 \overset{{ (0)}}{h}_{uA} \big ) = 0 \, , \end{aligned}$$

4.54 $$\begin{aligned}&\mathring{D}^{B} \mathring{D}_{A} \overset{{ (0)}}{h}_{u B} -\mathring{D}^{B} \mathring{D}_{B} \overset{{ (0)}}{h}_{u A} - 3 \partial _{u} \overset{{ (-1)}}{h}_{u A} +4 \alpha ^2 \overset{{ (-2)}}{h}_{u A} \nonumber \\&\quad +\mathring{D}_{A} \overset{{ (-1)}}{h}_{u u} =0. \end{aligned}$$

Using the identity

4.55 $$\begin{aligned} \mathring{D}^{B}\mathring{D}_{A} \overset{{ (2)}}{h}_{uB}=\mathring{D}_{A}\mathring{D}^{B} \overset{{ (2)}}{h}_{uB} +\overset{{ (2)}}{h}_{uA} \, , \end{aligned}$$

(4.52) becomes

4.56 $$\begin{aligned} -\big ( {\varDelta _{{{\mathring{{\gamma }}}}}}+ 1 \big ) \overset{{ (2)}}{h}_{uA} +\mathring{D}_{A} \mathring{D}^{B} \overset{{ (2)}}{h}_{uB} -2\alpha ^{2} \overset{{ (0)}}{h}_{uA} -\mathring{D}_{A}\overset{{ (1)}}{h}_{uu} = 0 \,. \end{aligned}$$

For $\alpha \ne 0$, substituting (4.40) we obtain

4.57 $$\begin{aligned} -\big ( {\varDelta _{{{\mathring{{\gamma }}}}}}+ 1 \big ) \overset{{ (2)}}{h}_{uA} -2\alpha ^{2} \overset{{ (0)}}{h}_{uA} = 0 \,. \end{aligned}$$

Regardless of whether $\alpha$ vanishes or not, we similarly substitute (4.55) into (4.54) to obtain an evolution equation for $\partial _{u} \overset{{ (-1)}}{h}_{u A}$

4.58 $$\begin{aligned} 3 \partial _{u} \overset{{ (-1)}}{h}_{u A}= & {} \mathring{D}_{A} \mathring{D}^{B} \overset{{ (0)}}{h}_{u B} -\big ( {\varDelta _{{{\mathring{{\gamma }}}}}}+ 1 \big )\overset{{ (0)}}{h}_{u A}\nonumber \\&+2 \overset{{ (0)}}{h}_{u A} +4 \alpha ^2 \overset{{ (-2)}}{h}_{u A} +\mathring{D}_{A} \overset{{ (-1)}}{h}_{u u} \,. \end{aligned}$$

Recall that $\overset{{ (0)}}{h}_{uA}= \mathring{D}^B \overset{{ (1)}}{h}_{AB}/2$ and $\overset{{ (-2)}}{h}_{uA}= -3/4 \mathring{D}^B \overset{{ (-1)}}{h}_{AB}$, both by (4.23), which allows us to rewrite (4.58) as

4.59 $$\begin{aligned} 3 \partial _{u} \overset{{ (-1)}}{h}_{u A}= & {} \frac{1}{2} \mathring{D}_{A} \mathring{D}^{B} \mathring{D}^{C} \overset{{ (1)}}{h}_{CB} + \frac{1}{2}\mathring{D}^B \overset{{ (1)}}{h}_{AB} -\frac{1}{2} {\varDelta _{{{\mathring{{\gamma }}}}}}\mathring{D}^B \overset{{ (1)}}{h}_{AB} \nonumber \\&-3 \alpha ^2 \mathring{D}^B \overset{{ (-1)}}{h}_{AB} +\mathring{D}_{A} \overset{{ (-1)}}{h}_{u u} \,. \end{aligned}$$

The asymptotically flat case is obtained by setting $\alpha =0$, compare [15, Equation (5.103)].

An alternative analysis of the equations $\mathscr {E}_{uu}=0$ and $\mathscr {E}_{uA}=0$ can be found in Appendix D.

### An example: the Blanchet–Damour solutions

An interesting class of linearised solutions of the linearised Einstein equations with $\varLambda =0$ has been introduced in [8]. We use these solutions to provide an explicit example of the behaviour both at the origin and at infinity of a vacuum metric perturbation in Bondi coordinates. In particular we calculate the news tensor for the Blanchet–Damour metrics, see (4.82).

In [8] the solutions are presented in harmonic coordinates, where the trace-reversed tensor

$$\begin{aligned} \bar{h}_{\mu \nu } {:}{=} h_{\mu \nu } - \frac{1}{2 } \eta ^{\alpha \beta } h_{\alpha \beta } \eta _{\mu \nu } \end{aligned}$$

satisfies

4.60 $$\begin{aligned} \Box _\eta \bar{h}_{\mu \nu } = 0 \,, \quad \partial _\mu \bar{h}^{\mu \nu } = 0 \,. \end{aligned}$$

Here $\eta$ is the Minkowski metric, taken to be $-(dx^0)^2+(dx^1)^2+(dx^2)^2+(dx^3)^2$ in the coordinates of (4.60), and $\Box _\eta$ the associated wave operator.

Given a collection of smooth functions $I_{ij}:\mathbb {R}\rightarrow \mathbb {R}$ such that $I_{ij}=I_{ji}$, the tensor field

4.61 $$\begin{aligned} \bar{h}_{tt}= & {} \partial _i \partial _j \left( \frac{I_{ij}(t-r) - I_{ij}(t+r)}{r} \right) \nonumber \\= & {} \big ( \ddot{I}_{ij}(t-r) - \ddot{I}_{ij}(t+r) \big ) \frac{x^i x^j }{r^3} + O(r^{-2}) \,, \end{aligned}$$

4.62 $$\begin{aligned} \bar{h}_{ti}= & {} \partial _j \left( \frac{\dot{I}_{ij}(t-r) - \dot{I}_{ij}(t+r)}{r} \right) \nonumber \\= & {} -\big ( \ddot{I}_{ij}(t-r) + \ddot{I}_{ij}(t+r) \big ) \frac{x^j }{r^2} + O(r^{-2}) \,, \end{aligned}$$

4.63 $$\begin{aligned} \bar{h}_{ij}= & {} \frac{\ddot{I}_{ij}(t-r) - \ddot{I}_{ij}(t+r)}{r} \,, \end{aligned}$$

where each dot represents a derivative with respect to the argument of $I_{ij}$, is a smooth tensor field on Minkowski spacetime solving (4.60).

Since the operators appearing in (4.60) commute with partial differentiation, further solutions can be constructed by applying $\partial _{\mu _1}\cdots \partial _{\mu _\ell }$ to $\bar{h}_{\mu \nu }$, and by applying Poincaré transformations.

Transforming to the cone-adapted coordinates $(u=t-r,r,x^A)$ one has

4.64 $$\begin{aligned} \partial _u = \partial _t \,, \quad \partial _r = \partial _t + \frac{x^i}{r}\partial _i \,, \quad \partial _A = \frac{\partial x^i}{\partial x^A} \partial _i\ \,. \end{aligned}$$

It follows that

$$\begin{aligned} h_{ti}= & {} \bar{h}_{ti} = \partial _j \left( \frac{\dot{I}_{ij}(t-r) - \dot{I}_{ij}(t+r)}{r} \right) \nonumber \\= & {} -\left( \frac{ \ddot{I}_{ij}(u) + \ddot{I}_{ij}(u+2r)}{r} + \frac{ \dot{I}_{ij}(u) - \dot{I}_{ij}(u+2r)}{ r^2} \right) \frac{ x^j}{r} , \nonumber \\ \bar{h}_{tt}= & {} ``\partial _i h_{ti} \text{ where } \text{ in } h_{ti} \text{ the } \text{ functions } \dot{I}_{ij} \text{ have } \text{ been }\nonumber \\&\text{ replaced } \text{ by } I_{ij}'' \nonumber \\= & {} \frac{\left( \ddot{I}_{ij}(u) - \ddot{I}_{ij}(u+2r)\right) }{ r} \frac{x^i x^j}{r^2} + \frac{\dot{I}_{ij}(u)+ \dot{I}_{ij}(u+2r)}{r^2} \nonumber \\&\times \left( 3 \frac{x^i x^j}{r^2} - \delta ^{ij} \right) + \frac{ I_{ij}(u) - I_{ij}(u+2r)}{ r^3}\nonumber \\&\times \left( 3 \frac{x^i x^j}{r^2} - \delta ^{ij} \right) \,, \nonumber \\ h_{uu}= & {} h_{tt} = \bar{h}_{tt} + \frac{1}{2} \bar{h}^{\mu }{}_\mu = \frac{1}{2} \big ( \bar{h}_{tt} +\bar{h}^{i}{}_i \big ) \nonumber \\= & {} \frac{ \ddot{I}_{ij}(u) - \ddot{I}_{ij}(u+2r) }{2 r} \left( \delta ^{ij} + \frac{x^i x^j}{r^2} \right) \nonumber \\&+ \frac{\dot{I}_{ij}(u)+ \dot{I}_{ij}(u+2r)}{2r^2} \left( 3 \frac{x^i x^j}{r^2} - \delta ^{ij} \right) \nonumber \\&+ \frac{ I_{ij}(u) - I_{ij}(u+2r)}{2 r^3} \left( 3 \frac{x^i x^j}{r^2} - \delta ^{ij} \right) \,, \end{aligned}$$

4.65 $$\begin{aligned} \bar{h}^\mu {}_{\mu }= & {} - \bar{h}_{tt} + \frac{\ddot{I}_{ii}(t-r) - \ddot{I}_{ii}(t+r)}{r} \nonumber \\= & {} \frac{\ddot{I}_{ij}(u) - \ddot{I}_{ij}(u+2r)}{r} \left( \delta ^{ij} - \frac{x^i x^j}{r^2} \right) \nonumber \\&- \frac{\dot{I}_{ij}(u)+ \dot{I}_{ij}(u+2r)}{r^2} \left( 3 \frac{x^i x^j}{r^2} - \delta ^{ij} \right) \nonumber \\&- \frac{ I_{ij}(u) - I_{ij}(u+2r)}{ r^3} \left( 3 \frac{x^i x^j}{r^2} - \delta ^{ij} \right) \,, \nonumber \\ h_{rr}= & {} \bar{h}_{rr} = \bar{h}_{tt} + 2 \bar{h}_{ti}\frac{x^i}{r} + \bar{h}_{ij}\frac{x^i x^j}{r^2} \nonumber \\= & {} - \frac{4\ddot{I}_{ij}(u+2r)}{ r} \frac{x^i x^j}{r^2} \nonumber \\&-\frac{\dot{I}_{ij}(u) }{ r^2} \left( \delta ^{ij} - \frac{x^i x^j}{r^2} \right) \nonumber \\&- \frac{\dot{I}_{ij}(u+2r)}{ r^2} \left( \delta ^{ij} - 5 \frac{x^i x^j}{r^2} \right) \nonumber \\&+ \frac{ I_{ij}(u) - I_{ij}(u+2r)}{ r^3} \left( 3 \frac{x^i x^j}{r^2} - \delta ^{ij} \right) \,, \nonumber \\ h_{rA}= & {} \bar{h}_{rA} = \left( \bar{h}_{tj} + \bar{h}_{ij} \frac{x^i}{r} \right) \frac{\partial x^j}{\partial x^A} \nonumber \\= & {} \left( \partial _j \left( \frac{\dot{I}_{ij}(t-r) - \dot{I}_{ij}(t+r)}{r}\right) \right. \nonumber \\&\left. + \frac{\ddot{I}_{ij}(t-r) - \ddot{I}_{ij}(t+r)}{r} \frac{x^j }{r} \right) \frac{\partial x^i}{\partial x^A} \nonumber \\= & {} - \left( \frac{2\ddot{I}_{ij}(u+2r) }{r} + \frac{\dot{I}_{ij}(u) - \dot{I}_{ij}(u+2r)}{r^2} \right) \frac{x^j }{r}\frac{\partial x^i}{\partial x^A} \,, \nonumber \\ \end{aligned}$$

4.66 $$\begin{aligned} h_{AB}= & {} \bar{h}_{AB} - \frac{1}{2} \bar{h}^{\mu }{}_\mu r^2 {{\mathring{{\gamma }}}}_{AB} = \bar{h}_{ij} \frac{\partial x^i}{\partial x^A} \frac{\partial x^j}{\partial x^B}- \frac{1}{2} \bar{h}^{\mu }{}_\mu r^2 {{\mathring{{\gamma }}}}_{AB} \nonumber \\= & {} \left( \frac{\ddot{I}_{ij}(u) - \ddot{I}_{ij}(u+2r)}{r} \right) \frac{\partial x^i}{\partial x^A} \frac{\partial x^j}{\partial x^B} \nonumber \\&- \frac{1}{2} \left( \frac{\ddot{I}_{ij}(u) - \ddot{I}_{ij}(u+2r)}{r} \left( \delta ^{ij} - \frac{x^i x^j}{r^2} \right) \right. \nonumber \\&- \frac{\dot{I}_{ij}(u)+ \dot{I}_{ij}(u+2r)}{r^2} \left( 3 \frac{x^i x^j}{r^2} - \delta ^{ij} \right) \nonumber \\&\left. - \frac{ I_{ij}(u) - I_{ij}(u+2r)}{ r^3} \left( 3 \frac{x^i x^j}{r^2} - \delta ^{ij} \right) \right) r^2 {{\mathring{{\gamma }}}}_{AB} \nonumber \\= & {} \left( \frac{\ddot{I}_{ij}(u) - \ddot{I}_{ij}(u+2r)}{r} \right) \frac{\partial x^i}{\partial x^A} \frac{\partial x^j}{\partial x^B} \nonumber \\&- \frac{\ddot{I}_{ij}(u) - \ddot{I}_{ij}(u+2r)}{2r} \left( \!\delta ^{ij} - \frac{x^i x^j}{r^2} \right) r^2 {{\mathring{{\gamma }}}}_{AB}\nonumber \\&+ O(r^{-2}). \,\,\, \end{aligned}$$

Finally (with all expansions in the last equation and below for large *r*),

4.67 $$\begin{aligned} h_{ur}= & {} \bar{h}_{ur} + \frac{1}{2} \bar{h}^{\mu }{}_\mu = \bar{h}_{tt} + \bar{h}_{ti} \frac{x^i}{r}+ \frac{1}{2} \bar{h}^{\mu }{}_\mu \nonumber \\= & {} \partial _i \partial _j \left( \frac{I_{ij}(t-r) - I_{ij}(t+r)}{r} \right) \nonumber \\&+ \partial _j \left( \frac{\dot{I}_{ij}(t-r) - \dot{I}_{ij}(t+r)}{r} \right) \frac{x^i}{r} \nonumber \\&-\partial _i \partial _j \left( \frac{I_{ij}(t-r) - I_{ij}(t+r)}{2r} \right) \nonumber \\&+ \frac{\ddot{I}_{ii}(t-r) - \ddot{I}_{ii}(t+r)}{2r} \nonumber \\= & {} \frac{\ddot{I}_{ij}(u) - \ddot{I}_{ij}(u+2r)}{2 r} \left( \delta ^{ij} + \frac{x^i x^j}{r^2} \right) \nonumber \\&-\frac{\ddot{I}_{ij}(u) + \ddot{I}_{ij}(u+2r)}{r} \frac{x^i x^j}{r^2} + O(r^{-2}) \,, \nonumber \\ h_{uA}= & {} \bar{h}_{uA} = \bar{h}_{ti} \frac{\partial x^i}{\partial x^A} \nonumber \\= & {} \partial _j \left( \frac{\dot{I}_{ij}(t-r) - \dot{I}_{ij}(t+r)}{r} \right) \frac{\partial x^i}{\partial x^A} \nonumber \\= & {} -\frac{\ddot{I}_{ij}(u) + \ddot{I}_{ij}(u+2r)}{r} \frac{\partial x^i}{\partial x^A} \frac{x^j}{r} + O(r^{-2}) \,. \end{aligned}$$

To continue, for simplicity we assume that all the $I_{ij}$’s vanish for sufficiently large arguments, and that *r* is so large that all the $I_{ij}(u+2r)$’s vanish. The vector field ${\xi }$ which brings the metric perturbation to the Bondi form is (cf. Sect. 3.1)

4.68 $$\begin{aligned} {\zeta }^{{u}}= & {} {\xi }^{{u}}({u},x^A) +\frac{\dot{I}_{ij}(u) }{2 r} \left( \delta ^{ij} - \frac{x^i x^j}{r^2} \right) \nonumber \\&- \frac{ I_{ij}(u) }{4 r^2} \left( 3\frac{x^i x^j}{r^2} - \delta ^{ij} \right) \,, \end{aligned}$$

4.69 $$\begin{aligned} {\zeta }^{B}= & {} {\xi }^{B} ({u},x^A) + \mathring{D}^{B} \bigg [ -\frac{1}{{r}} {\xi }^{{u}}({u},x^A) + \frac{ I_{ij}(u) }{4r^3} \frac{x^i x^j}{r^2} \bigg ] \, ,\nonumber \\ \end{aligned}$$

4.70 $$\begin{aligned} r \mathring{D}_B {\zeta }^{B}= & {} r \mathring{D}_B {\xi }^{B} ({u},x^A) - \!\varDelta _{{{\mathring{{\gamma }}}}} {\xi }^{{u}}({u},x^A) \nonumber \\&+ \frac{ I_{ij}(u) }{4 r^2} \varDelta _{{{\mathring{{\gamma }}}}} \bigg ( \frac{x^i x^j}{r^2} \bigg ), \,\, \end{aligned}$$

4.71 $$\begin{aligned} {\zeta }^{{r}}= & {} -\frac{1}{2} {r}\mathring{D}_{B} {\zeta }^{B} - \frac{1}{4} {r}^{-1} {{\mathring{{\gamma }}}}^{AB} h_{AB} \nonumber \\= & {} -\frac{1}{2} {r}\mathring{D}_{B} {\zeta }^{B} - \frac{r h_{ij}}{4} \bigg ( \delta ^{ij} - \frac{x^ix^j}{r^2} \bigg ) \nonumber \\= & {} -\frac{1}{2} {r}\mathring{D}_{B} {\zeta }^{B} - \frac{\ddot{I}_{ij}(u)}{4} \bigg ( \delta ^{ij} - \frac{x^ix^j}{r^2} \bigg ) + \frac{r\bar{h} ^\mu {}_\mu }{4} \nonumber \\= & {} -\frac{1}{2} {r}\mathring{D}_{B} \xi ^{B} + \frac{1}{2} \varDelta _{{\mathring{{\gamma }}}}\xi ^u - \frac{\dot{I}_{ij}(u) }{4r} \left( 3 \frac{x^i x^j}{r^2} - \delta ^{ij} \right) \,. \nonumber \\ \end{aligned}$$

To obtain the mass aspect function one needs the gauge-transformed $h_{uu}$

4.72 $$\begin{aligned} h_{uu} \rightarrow h_{uu}+ \mathscr {L}_\zeta \eta _{uu} =h_{uu}- 2 \partial _{{u}} \big ( \zeta ^{{u}} +\zeta ^r \big ) \,. \end{aligned}$$

After applying a *u*-derivative to (4.71), we will obtain a solution for which $h_{uu}$ tends to zero as *r* tends to infinity in Bondi gauge if and only if

4.73 $$\begin{aligned} (\varDelta _{{\mathring{{\gamma }}}}+2)\partial _u \xi ^u = 0 \,, \quad \mathring{D}_A\partial _u \xi ^A = 0 \,, \end{aligned}$$

In the calculations that follow the identities (3.32) are useful; we repeat them here for the convenience of the reader:

4.74 $$\begin{aligned}&\mathring{\gamma }^{AB}\frac{\partial x^{i}}{\partial x^{A}}\frac{\partial x^{j}}{\partial x^{B}} = (\delta ^{ij} - \frac{x^{i}x^{j}}{r^{2}})r^{2} \,, \qquad \mathring{D}^A(x^i \mathring{D}_A x^i) = 0 \,, \nonumber \\ \end{aligned}$$

4.75 $$\begin{aligned}&\displaystyle \mathring{D}^C \bigg ( \frac{x^{(j} }{r^2}\frac{\partial x^{i)}}{\partial x^C} \bigg ) = \delta ^{ij} - \frac{3 x^i x^j}{r^2}\,, \quad \varDelta _{{\mathring{{\gamma }}}}\frac{x^i x^j}{r^2}= 2 \delta ^{ij} -6 \frac{x^i x^j}{r^2} \,. \nonumber \\ \end{aligned}$$

One then finds that in the linearised Bondi gauge the original field $h_{uu}$ becomes

4.76 $$\begin{aligned} h_{uu}&\rightarrow h_{uu} - \frac{ \ddot{I}_{ij}(u) }{2 r} \left( 3 \delta ^{ij} - 5\frac{x^i x^j}{r^2} \right) +O(r^{-2}) \nonumber \nonumber \\&= \frac{ \ddot{I}_{ij}(u) }{ r} \left( \frac{3 x^i x^j}{r^2} - \delta ^{ij} \right) +O(r^{-2}) \,. \end{aligned}$$

Let $\delta \mu$ denote the linearised mass aspect function, thus $h_{uu}= 2 \delta \mu /r + O(r^{-2})$. We see that

4.77 $$\begin{aligned} \delta \mu= & {} \frac{\ddot{I}_{ij}(u) }{2} \left( \frac{3 x^i x^j}{r^2} - \delta ^{ij} \right) \,. \end{aligned}$$

The function $\chi$ of (D.4) can be calculated by inspecting the asymptotic behaviour, for large *r*, of the functions appearing there. One finds

4.78 $$\begin{aligned} \chi= & {} 4 \delta \mu - \frac{1}{2} ({\varDelta _{{{\mathring{{\gamma }}}}}}+2){\varDelta _{{{\mathring{{\gamma }}}}}}{\xi }^{{u}} \nonumber \\= & {} 2\ddot{I}_{ij}(u) \left( \frac{3 x^i x^j}{r^2} - \delta ^{ij} \right) - \frac{1}{2} ({\varDelta _{{{\mathring{{\gamma }}}}}}+2){\varDelta _{{{\mathring{{\gamma }}}}}}{\xi }^{{u}} \,. \end{aligned}$$

It then follows from (D.3) that

4.79 $$\begin{aligned} \mathring{D}^A {h}_{uA} \big |_{r=0} = 2 \ddot{I}_{ij}(u) \left( \frac{3 x^i x^j}{r^2} - \delta ^{ij} \right) - \frac{1}{2} ({\varDelta _{{{\mathring{{\gamma }}}}}}+2){\varDelta _{{{\mathring{{\gamma }}}}}}{\xi }^{{u}} \,. \end{aligned}$$

This equation looks surprising at first sight, since the left-hand side is zero for a smooth tensor field in the $(u,r,x^A)$ coordinates. However, (4.79) makes it clear that the linearised Bondi gauge introduces a singular behaviour of the gauge-transformed metric perturbation at $r=0$. This singularity can be removed on some chosen light cone, say $\mathscr {C}_{u_0}$, but cannot be removed for all *u* by a residual gauge transformation (in other words, asymptotic symmetry) in general.

Recall the formula for the gauge-transformed $h_{AB}$:

4.80 $$\begin{aligned} h_{AB}\rightarrow & {} h_{AB}+ 2 r \zeta ^r {{\mathring{{\gamma }}}}_{AB} + {r}^2 \mathring{{\mathcal {L}}}_{\zeta } {{\mathring{{\gamma }}}}_{AB} \,, \end{aligned}$$

where $\mathring{{\mathcal {L}}}_{\zeta }$ denotes the two-dimensional Lie derivative with respect to the field $\zeta ^A\partial _A$. This leads to

4.81 $$\begin{aligned} h_{AB} \rightarrow&\left( \frac{\ddot{I}_{ij}(u) - \ddot{I}_{ij}(u+2r)}{r} \right) \frac{\partial x^i}{\partial x^A} \frac{\partial x^j}{\partial x^B} \nonumber \\&- {{\mathring{{\gamma }}}}^{CD} \left( \frac{\ddot{I}_{ij}(u) - \ddot{I}_{ij}(u+2r)}{2r} \right) \frac{\partial x^i}{\partial x^C} \frac{\partial x^j}{\partial x^D} {{\mathring{{\gamma }}}}_{AB}\nonumber \\&+ O(r^{-3}) \,, \end{aligned}$$

from which the news tensor $\partial _u \overset{{ (1)}}{h}_{AB}$ is readily obtained:

4.82 $$\begin{aligned} \partial _u \overset{{ (1)}}{h}_{AB} = \dddot{I}_{ij}(u) \left( \frac{\partial x^i}{\partial x^A} \frac{\partial x^j}{\partial x^B} - \frac{1}{2} {{\mathring{{\gamma }}}}^{CD} \frac{\partial x^i}{\partial x^C} \frac{\partial x^j}{\partial x^D} {{\mathring{{\gamma }}}}_{AB} \right) \,. \nonumber \\ \end{aligned}$$

## The energy of weak gravitational fields

### Trautman–Bondi mass

When $\varLambda =0$, the Trautman–Bondi mass is determined from the function *V* appearing in the metric. Based on the variational arguments so far, one expects that its linearised-theory equivalent will be determined by the second variation of *V*. Our aim in this section is to derive a formula for this second variation.

Recall that the one-parameter families of vacuum metrics $g_{\mu \nu }(\lambda , \cdot )$ which define the variations considered here satisfy

5.1 $$\begin{aligned} e^{2\beta } |_{\lambda =0}= 1 \,, \quad V |_{\lambda =0}= r - \frac{\varLambda r^3}{3} \,, \quad \partial _r V |_{\lambda =0} = 1 -{\varLambda }r^2 \,. \end{aligned}$$

Using (5.1) and our remaining asymptotic conditions, the second variation equation is obtained by differentiating (4.31) twice with respect to $\lambda$ and reads (cf. (5.10) below)

5.2 $$\begin{aligned}&2 \partial _r \delta ^2 V { + } 4 ( \varLambda r^2 -1) \delta ^2 \beta = \delta ^2 \mathscr {R} - \frac{1}{r^2 }\mathring{D}^A \Big [ \partial _r (r^4\delta \check{h}_{u A})\Big ] \nonumber \\&\quad -2 {{\mathring{{\gamma }}}}^{AB} \mathring{D}_{A} \mathring{D}_{B} \delta ^2 \beta - r^4 {{\mathring{{\gamma }}}}^{AB}(\partial _r \check{h}_{uA})(\partial _r \check{h}_{uB}) \nonumber \\&\quad - 8\pi \delta ^2 \Big [ {\gamma }^{AB}T_{AB}-r^2 T^{a}_{a}\Big ] \nonumber \\&\quad + \frac{2}{r^2}\mathring{D}^A \Big [ \partial _r (r^4 {{\mathring{{\gamma }}}}^{BD} \check{h}_{A B} \check{h}_{u D})\Big ] \,. \end{aligned}$$

We need the second variation of (4.10), which reads

5.3 $$\begin{aligned} \partial _r \delta ^2\beta = \frac{r}{8}{{\mathring{{\gamma }}}}^{AC}{{\mathring{{\gamma }}}}^{BD} \partial _r \check{h}_{AB}\partial _r \check{h}_{CD} + 2\pi r \delta ^2 T_{rr} \,, \end{aligned}$$

and which can be explicitly integrated after requiring that $\delta ^2\beta$ goes to zero as *r* tends to infinity:

5.4 $$\begin{aligned} \delta ^2\beta = - \int _ r^\infty \left( \frac{r}{8}{{\mathring{{\gamma }}}}^{AC}{{\mathring{{\gamma }}}}^{BD} \partial _r \check{h}_{AB}\partial _r \check{h}_{CD} + 2\pi r \delta ^2 T_{rr} \right) dr. \end{aligned}$$

(We note that this is negative if we impose the dominant energy condition.) In vacuum and for large *r* we obtain the expansion

5.5 $$\begin{aligned} \delta ^2 \beta= & {} - \frac{1}{16r^2 }{{\mathring{{\gamma }}}}^{AC}{{\mathring{{\gamma }}}}^{BD} \overset{{ (-1)}}{\check{h}}_{AB} \left( \overset{{ (-1)}}{\check{h}}_{CD} + \frac{3}{ r^2} \overset{{ (-3)}}{\check{h}}_{CD} \right) \nonumber \\&+ O (r^{-5}) \,, \end{aligned}$$

while for small *r* we have, using (3.29),

5.6 $$\begin{aligned} \delta ^2 \beta = -\frac{1}{8 r^2 } \mathring{D}^A\mathring{D}^B \xi ^u ( 2 \mathring{D}_A\mathring{D}_B \xi ^u -\varDelta _{{\mathring{{\gamma }}}}\xi ^u {{\mathring{{\gamma }}}}_{AB} ) + O (r^{-1}) \,. \end{aligned}$$

We see that $\delta ^2\beta$ diverges badly at the origin unless $\xi ^u$ is a linear combination of $\ell =0$ and $\ell =1$ spherical harmonics. Now, as explained in Sect. 3, the field $\xi ^u$ is determined by the linearised metric up to the choice of a *u*-independent initial datum. Hence, given a light cone we can always find a gauge so that the leading-order singularity above vanishes. Equation (3.29) shows that in this gauge the integrand in (5.4) is bounded and $\delta ^2 \beta$ is finite everywhere.

In the remainder of this section we assume a vacuum metric perturbation, and a gauge so that $\delta ^2\beta$ is bounded on the light cone under consideration.

We rewrite (5.2) as

5.7 $$\begin{aligned}&2 \partial _r \delta ^2 V = \delta ^2 \mathscr {R} - \frac{1}{r^2 }\mathring{D}_A \Big [ \partial _r (r^4\delta \check{h}_u{}^A)\Big ] - { 4}( \varLambda r^2 -1) \delta ^2 \beta \nonumber \\&\quad - 2 {{\mathring{{\gamma }}}}^{AB} \mathring{D}_{A} \mathring{D}_{B} \delta ^2 \beta - r^4 {{\mathring{{\gamma }}}}^{AB}\partial _r \check{h}_{uA} \partial _r \check{h}_{uB} \nonumber \\&\quad + \frac{2}{r^2}\mathring{D}^A \Big [ \partial _r (r^4 {{\mathring{{\gamma }}}}^{BD} \check{h}_{A B} \check{h}_{u D}) \Big ] \,. \end{aligned}$$

Recall that for $\varLambda =0$ the Trautman–Bondi mass $m_{{\mathrm {TB}}}$ is defined as [9, 53, 54]

5.8 $$\begin{aligned} m_{\mathrm {TB}}= -\frac{1}{8\pi }\int _{S^2} \overset{{ (0)}}{V} d^2\mu _{{{\mathring{{\gamma }}}}} \,, \end{aligned}$$

where $\overset{{ (0)}}{V}$ is the *r*-independent coefficient in an asymptotic expansion of *V*. It was proposed in [13] to use this definition for $\varLambda \ne 0$, which was motivated by Cauchy-problem considerations.

Before proceeding further, recall that a one-parameter family of metrics $\lambda \mapsto g_{\mu \nu }(\lambda )$ in Bondi gauge satisfies the condition

5.9 $$\begin{aligned} \sqrt{\det g_{AB}(\lambda )} = \sqrt{\det g_{AB}(0)} \,. \end{aligned}$$

This implies in particular

5.10 $$\begin{aligned} \frac{d\sqrt{\det g_{AB}(\lambda )}}{d\lambda } = 0= \frac{d^2 \sqrt{\det g_{AB}(\lambda )}}{d\lambda ^2} \,. \end{aligned}$$

Equivalently,

5.11 $$\begin{aligned} \delta \sqrt{\det g_{AB} } = 0= \delta ^2 \sqrt{\det g_{AB} } \,. \end{aligned}$$

It then follows from the Gauss–Bonnet theorem that

5.12 $$\begin{aligned} \delta \int _{S^2} \mathscr {R} \, d^2\mu _\gamma = 0 = \delta ^2 \int _{S^2} \mathscr {R} \, d^2\mu _\gamma \,, \end{aligned}$$

Integrating (5.7) over a truncated cone

$$\begin{aligned} \mathscr {C}_{u,R} = \mathscr {C}_u \cap \{0\le r \le R\} \end{aligned}$$

one obtains

5.13 $$\begin{aligned} \delta ^2 m_{\mathrm {TB}}(\mathscr {C}_{u,R})&: =&-\frac{1}{8\pi }\int _{r=R} \delta ^2 V d^2\mu _{{{\mathring{{\gamma }}}}} \nonumber \\= & {} \frac{1}{16 \pi } \int _{\mathscr {C}_{u,R}} \left( r^4 {{\mathring{{\gamma }}}}^{AB}\partial _r \check{h}_{uA} \partial _r \check{h}_{uB}\right. \nonumber \\&\left. - { 4}(1- \varLambda r^2 ) \delta ^2 \beta \right) \, dr \, d\mu _{{{\mathring{{\gamma }}}}} \,. \end{aligned}$$

When $\varLambda \le 0$ we have $\delta ^2\beta \le 0$, which proves positivity of $\delta ^2 m_{\mathrm {TB}}(\mathscr {C}_{u,R})$, and of its limit $\delta ^2 m_{\mathrm {TB}}(\mathscr {C}_{u })$ with $R\rightarrow \infty$.

The notation in (5.13) is somewhat misleading, as the limit as *R* goes to infinity of (5.13) will only reproduce the Trautman–Bondi mass if this limit converges. This is the case when $\varLambda \le 0$ (compare (5.105), Sect. 5.8). However, when $\varLambda >0$ the integrand for large *r* behaves as

5.14 $$\begin{aligned} - \frac{\varLambda r}{4} {{\mathring{{\gamma }}}}^{AC}{{\mathring{{\gamma }}}}^{BD} \overset{{ (-1)}}{\check{h}}_{AB} \overset{{ (-1)}}{\check{h}}_{CD} + O (r^{-1}) \,, \end{aligned}$$

so that

5.15 $$\begin{aligned} \delta ^2 m_{\mathrm {TB}}(\mathscr {C}_{u})&{:}{=}-\frac{1}{8\pi }\int _{S^2} \delta ^2 \overset{{ (0)}}{V} d^2\mu _{{{\mathring{{\gamma }}}}} \end{aligned}$$

5.16 $$\begin{aligned}= & {} \displaystyle \lim _{R\rightarrow \infty } \frac{1}{16 \pi } \left( \int _{\mathscr {C}_{u,R}} \displaystyle \left( r^4 {{\mathring{{\gamma }}}}^{AB}\partial _r \check{h}_{uA} \partial _r \check{h}_{uB}\nonumber \right. \right. \\&\left. - { 4}(1- \varLambda r^2 ) \delta ^2 \beta \right) \, dr \, d\mu _{{{\mathring{{\gamma }}}}} \nonumber \\&\left. + \frac{\varLambda R}{4} \int _{S_R} {{\mathring{{\gamma }}}}^{AC}{{\mathring{{\gamma }}}}^{BD} \overset{{ (-1)}}{\check{h}}_{AB} \overset{{ (-1)}}{\check{h}}_{CD} \, d\mu _{{{\mathring{{\gamma }}}}} \right) \,. \nonumber \\ \end{aligned}$$

### Boundary integrand and $\varLambda$

We assume now that the background takes the form

5.17 $$\begin{aligned} {g}_{\alpha \beta } dx^\alpha dx^\beta = \epsilon N^2 du^2-2du \, dr + r^2 \underbrace{(d\theta ^2+\sin ^2 \theta d\phi ^2)}_{{=}{:}{\mathring{{\gamma }}}} \,, \end{aligned}$$

which is compatible both with the Minkowski and the de Sitter metrics. We suppose that the metric perturbations $\delta g_{\mu \nu }$ satisfy the Bondi gauge conditions:

5.18 $$\begin{aligned} \delta g_{rr}=0 = \delta g_{rA}= g^{AB}\delta g_{AB} \,. \end{aligned}$$

This implies

5.19 $$\begin{aligned} \nabla _{ \mu }\delta g_{r r}=0 = \nabla _{u}\delta g_{r A} = \nabla _{r}\delta g_{r A} \,, \end{aligned}$$

and

$$\begin{aligned}&\displaystyle \nabla _{u} \delta g_{u u} = \partial _{u}(\delta g_{u u}) -2 \epsilon {N}^{'} {N}\left( \delta g_{u u}+ \epsilon {N}^2 \delta g_{u r} \right) \,,\\&\quad \nabla _{u}\delta g_{u r} =\partial _{u}(\delta g_{u r}) \,, \\&\nabla _{u}\delta g_{AB} =\partial _{u}(\delta g_{A B})\,, \quad \nabla _{u}\delta g_{u A} =\partial _{u}(\delta g_{u A})- \epsilon {N}^{'} {N}\delta g_{u A} \,, \\&\displaystyle \nabla _{r} \delta g_{u u} = \partial _{r}(\delta g_{u u}) + 2 \epsilon {N}^{'} {N}\delta g_{u r} \,, \quad \nabla _{r} \delta g_{u r} = \partial _{r} ( \delta g_{u r}) \,,\\&\nabla _{r}\delta g_{AB} =\partial _{r}(\delta g_{AB})-\frac{2}{r}\delta g_{AB}\,, \\&\nabla _{r}\delta g_{u A} =\partial _{r}(\delta g_{u A})- \frac{1}{r} \delta g_{u A} \,,\\&\quad \displaystyle \nabla _A \delta g_{u u} = \partial _A (\delta g_{u u}) \,, \nabla _A \delta g_{u r} = \partial _{A} (\delta g_{u r})-\frac{1}{r} \delta g_{u A} \\&\nabla _{A}\delta g_{u B} =\mathring{D}_{A}(\delta g_{u B})- \frac{1}{r} g_{AB} \left( \delta g_{u u}+ \epsilon {N}^2 \delta g_{u r}\right) \,,\\&\nabla _{A}\delta g_{BC} = \mathring{D}_{A} ( \delta g_{BC} ) -\frac{2}{r} g_{A(B} \delta g_{C) u} \,, \\&\quad \nabla _{A}\delta g_{r B} =-\frac{1}{r}\left( \delta g_{AB}+ g_{AB} \delta g_{u r} \right) \,. \end{aligned}$$

Under the above assumptions, the functional $b^r(\delta _1 g,\delta _2 g)$ given by (B.14), Appendix B below becomes

5.20 $$\begin{aligned}&b^{{r}} (\delta _1 g,\delta _2 g) =-\frac{1}{r^2}\delta _1g_{{u}{r}} {\mathring{{\gamma }}}^{A B} \nabla _{B}(\delta _2g_{{u}B} +\epsilon {N}^2 \delta _2g_{{r}B}\nonumber \\&\quad + \frac{1}{2r^4} {\mathring{{\gamma }}}^{A C} {\mathring{{\gamma }}}^{B D}\delta _1g_{C D} \big ( \epsilon {N}^2 \nabla _{{r}}\delta _2g_{AB} + \nabla _{{u}}\delta _2g_{AB} \nonumber \\&\quad -2\nabla _A(\epsilon {N}^2 \delta _2g_{{r}B} + \delta _2g_{{u}B} ) \big )\nonumber \\&\quad + \frac{1}{r^2} {\mathring{{\gamma }}}^{AC}\delta _1 g_{{u}C} \left( \nabla _{{r}} \delta _2 g_{{u}A} + \frac{1}{2r^2} {\mathring{{\gamma }}}^{B D} \nabla _{A} \delta _2 g_{B D} \right) .\nonumber \\ \end{aligned}$$

After some further algebra one obtains

5.21 $$\begin{aligned}&b^{{r}} (\delta _1 g,\delta _2 g)\nonumber \\&\quad =\frac{1}{r}\delta _1g_{{u}{r}} \left[ 2(1+2 \epsilon {N}^2) \delta _2g_{{u}{r}} - \frac{1}{r} {\mathring{{\gamma }}}^{C D} \mathring{D}_C\delta _2 g_{u D} \right] \nonumber \\&\quad + \frac{1}{2r^4} {\mathring{{\gamma }}}^{A C} {\mathring{{\gamma }}}^{B D}\delta _1g_{C D} \big ( \epsilon {N}^2 \partial _{{r}}\delta _2g_{AB} + \partial _{{u}}\delta _2g_{AB}\nonumber \\&\quad - 2 \mathring{D}_A\delta _2 g_{u B} \big )+\frac{1}{r^2} \delta _{1}g_{{u}A} {\mathring{{\gamma }}}^{A B} \left( \partial _{{r}}\delta _{2}g_{{u}B} - \frac{2}{r}\delta _{2}g_{{u}B} \right) \, .\nonumber \\ \end{aligned}$$

Upon antisymmetrisation the first and last terms above drop out:

5.22 $$\begin{aligned}&{w}^{{r}} (\delta _1 g,\delta _2 g) \equiv b^{{r}} (\delta _2 g,\delta _1 g)- b^{{r}} (\delta _1 g,\delta _2 g) \nonumber \\&\quad = \frac{1}{r^2} {\mathring{{\gamma }}}^{C D} \left( \delta _1g_{{u}{r}} \mathring{D}_C\delta _2 g_{u D} - \delta _2g_{{u}{r}} \mathring{D}_C\delta _1 g_{u D} \right) \nonumber \\&\qquad + \frac{1}{2r^4} {\mathring{{\gamma }}}^{A C} {\mathring{{\gamma }}}^{B D}\Big (\delta _2g_{C D} \big ( \epsilon {N}^2 \partial _{{r}}\delta _1g_{AB} + \partial _{{u}}\delta _1g_{AB}\nonumber \\&\qquad - 2 \mathring{D}_A\delta _1 g_{u B } \big )\nonumber \\&\qquad - \delta _1g_{C D}\big ( \epsilon {N}^2 \partial _{{r}}\delta _2g_{AB} + \partial _{{u}}\delta _2g_{AB} - 2 \mathring{D}_A\delta _2 g_{u B} \big ) \Big )\nonumber \\&\qquad +\frac{1}{r^2} {\mathring{{\gamma }}}^{A B} \left( \delta _{2}g_{{u}A} \partial _{{r}}\delta _{1}g_{{u}B} - \delta _{1}g_{{u}A} \partial _{{r}}\delta _{2}g_{{u}B} \right) \, . \end{aligned}$$

#### Large *r*

We use the following asymptotics for the linearised metric components, as derived in Sect. 4.1 under the conditions there:

5.23 $$\begin{aligned} \delta g_{u r}\equiv & {} 0 \ \, \equiv \ \, \delta g_{r A} \ \, \equiv \ \,\delta g_{rr} \,, \end{aligned}$$

5.24 $$\begin{aligned} \delta g_{AB}= & {} \delta \overset{{ (1)}}{g}_{A B} r_{{}}+ o(1) \,, \end{aligned}$$

5.25 $$\begin{aligned} \delta g_{u A}= & {} \delta \overset{{ (2)}}{g}_{u A} r_{{}}^2 + \delta \overset{{ (0)}}{g}_{u A} + o(1) \,, \end{aligned}$$

5.26 $$\begin{aligned} \delta g_{u u}= & {} \delta \overset{{ (1)}}{g}_{u u} r +\frac{\delta \overset{{ (-1)}}{g}_{u u}}{r_{{}}} + o(r_{{}}^{-1}) \,, \end{aligned}$$

one finds

5.27 $$\begin{aligned}&r^2 b^{r}(\delta _1 g,\delta _2 g) \, \nonumber \\&=\quad r \Big [ -2 {\mathring{{\gamma }}}^{AB} \delta _1 \overset{{ (2)}}{g}_{u A} \delta _2 \overset{{ (0)}}{g}_{u B} \nonumber \\&\qquad + {\mathring{{\gamma }}}^{AC} {\mathring{{\gamma }}}^{BD} \Big ( \frac{\alpha ^2}{2 } \delta _1\overset{{ (1)}}{g}_{AB} \delta _2\overset{{ (1)}}{g}_{CD} - \delta _1\overset{{ (1)}}{g}_{AB} \mathring{D}_C\delta _2 \overset{{ (2)}}{g}_{u D} \Big ) \Big ] \nonumber \\&\qquad -3 {\mathring{{\gamma }}}^{AB}\delta _1\overset{{ (2)}}{g}_{u A} \delta _2\overset{{ (-1)}}{g}_{u B} + \frac{1}{2} {\mathring{{\gamma }}}^{AC} {\mathring{{\gamma }}}^{BD} \delta _1\overset{{ (1)}}{g}_{AB} \partial _{u} \delta _2\overset{{ (1)}}{g}_{CD}\nonumber \\&\qquad + o(1) \,. \end{aligned}$$

Hence

5.28 $$\begin{aligned}&r^2 \omega ^{r}(\delta _1 g,\delta _2 g) = r^2 \big ( b^{r}(\delta _2 g,\delta _1 g) - b^{r}(\delta _1 g,\delta _2 g) \big ) \nonumber \\&\quad = r \Big [ 2 {\mathring{{\gamma }}}^{AB} ( \delta _1 \overset{{ (2)}}{g}_{u A} \delta _2 \overset{{ (0)}}{g}_{u B} - \delta _2 \overset{{ (2)}}{g}_{u A} \delta _1 \overset{{ (0)}}{g}_{u B})\nonumber \\&\qquad + {\mathring{{\gamma }}}^{AC} {\mathring{{\gamma }}}^{BD} \Big ( \delta _1\overset{{ (1)}}{g}_{AB} \mathring{D}_C\delta _2 \overset{{ (2)}}{g}_{u D} - \delta _2\overset{{ (1)}}{g}_{AB} \mathring{D}_C\delta _1 \overset{{ (2)}}{g}_{u D} \Big ) \Big ] \nonumber \\&\qquad +3 {\mathring{{\gamma }}}^{AB} ( \delta _1\overset{{ (2)}}{g}_{u A} \delta _2\overset{{ (-1)}}{g}_{u B} -\delta _2\overset{{ (2)}}{g}_{u A} \delta _1\overset{{ (-1)}}{g}_{u B} ) \nonumber \\&\qquad + \frac{1}{2} {\mathring{{\gamma }}}^{AC} {\mathring{{\gamma }}}^{BD} ( \delta _2\overset{{ (1)}}{g}_{AB} \partial _{u} \delta _1\overset{{ (1)}}{g}_{CD} - \delta _1\overset{{ (1)}}{g}_{AB} \partial _{u} \delta _2\overset{{ (1)}}{g}_{CD})\nonumber \\&\qquad + o(1) \,. \end{aligned}$$

### The energy and its flux

We return now to a linearised vacuum gravitation field in Bondi gauge,

5.29 $$\begin{aligned} h&\equiv h_{\mu \nu }dx^\mu dx^\nu = h_{uu} du^2 + 2 h_{uA} dx^A du + h_{AB} dx^A dx^ B \nonumber \\&{=}{:} r^2\big ( \check{h}_{uu} du^2 + \underbrace{{{\mathring{{\gamma }}}}_{AB} {{\check{h}}_{u}{}} ^B}_{{=}{:}{{\check{h}}_{u}{}} _A} dx^ B du + \check{h}_{AB} dx^A dx^ B \big ) \,. \end{aligned}$$

Let, as before, $\mathscr {C}_{u,R}$ denote a light cone $\mathscr {C}_u$ truncated at radius *R*, and let $E_c[h,\mathscr {C}_{u,R}]$ denote the canonical energy contained in $\mathscr {C}_{u,R}$, defined using the vector field $\partial _u$:

5.30 $$\begin{aligned} E_c[h,\mathscr {C}_{u,R}]{:}{=} {\,\,\,\widetilde{\mathscr {H}}}[\mathscr {C}_{u,R}, \partial _u] \,. \end{aligned}$$

Recall that the Bondi gauge introduces singular behaviour at the tip of the light cone in general. When integrating formulae such as (2.30) over $\mathscr {S}$ one obtains a “boundary integral at $r=0$”. One can keep track of this but the resulting formulae do not seem to be very enlightening, so in what follows we can, and will, choose a Bondi gauge so that the (freely specifiable) gauge vector field $\xi$ in (3.9), which is part of the transformation which takes the metric from a smooth gauge to a Bondi gauge, satisfies

5.31 $$\begin{aligned} \mathring{D}_A\mathring{D}_B \xi ^u = \frac{1}{2} \varDelta _{{\mathring{{\gamma }}}}\xi ^u {{\mathring{{\gamma }}}}_{AB} \end{aligned}$$

at *a fixed light cone* $\mathscr {C}_u$ *under consideration*. (Compare (5.41)–(5.40) below and (3.29).) Equivalently, $\xi ^u$ is a linear combination of $\ell =0$ and $\ell =1$ spherical harmonics.

We note that this choice is tied to the chosen light cone $\mathscr {C}_u$, and will *not* be satisfied by nearby light cones in general. This turns out to be irrelevant for the calculations in this section.

We further note that the Bondi gauge is mainly relevant for us for the analysis of the field at large distances. One can sweep under the carpet the problem of the singularity at the origin by using Bondi coordinates for large *r*, and any other coordinates for small *r*. It follows from Sect. 2.3.3 that the total energy does not depend upon the choice of the coordinates for small *r*. The volume integral will then not take the simple form presented here for “non-Bondi” values of *r*, but this would again be irrelevant from the point of view of the large-*r* analysis of the fields.

Putting together our calculations so far, namely (2.124)–(2.126), (2.136), (2.138), (2.167) and (5.27) we find:

5.32 $$\begin{aligned}&E_c[h,\mathscr {C}_{u,R}] \nonumber \\&\quad = \frac{1}{64 \pi } \int _{\mathscr {C}_{u,R}} {{g}}^{BE } {{g}}^{FC} \big (\partial _u h _{BC } \partial _{ r } h_{EF } - h_{BC } \partial _{ r } \partial _u h_{EF } \big )\, d\mu _{\mathscr {C}}\nonumber \\&\qquad + {\frac{R}{64 \pi } } \int _{S^2} \left( 4{{\mathring{{\gamma }}}}^{AB} \overset{{ (0)}}{\check{h}}_{uA} \overset{{ (-2)}}{\check{h}}_{uB}- {{\mathring{{\gamma }}}}^{AB}{{\mathring{{\gamma }}}}^{CD} \right. \nonumber \\&\qquad \left. \times \left( \alpha ^2 \overset{{ (-1)}}{\check{h}}_{AC} \overset{{ (-1)}}{\check{h}}_{BD} - 2 \overset{{ (-1)}}{\check{h}}_{AC} \mathring{D}_B \overset{{ (0)}}{{{\check{h}}_{u}{}} }{}_{D} \right) \right) d\mu _{\mathring{\gamma }} \nonumber \\&\qquad {+ {\frac{1}{64 \pi } } \int _{S^2} \left( 6{{\mathring{{\gamma }}}}^{AB} \overset{{ (0)}}{\check{h}}_{uA} \overset{{ (-3)}}{\check{h}}_{uB} - {{\mathring{{\gamma }}}}^{AB}{{\mathring{{\gamma }}}}^{CD} \overset{{ (-1)}}{\check{h}}_{AC} \partial _{u} \overset{{ (-1)}}{\check{h}}_{BD} \right. }\nonumber \\&\qquad {\left. - {{\mathring{{\gamma }}}}^{AB}{{\mathring{{\gamma }}}}^{CD}\left( {\alpha ^2} \overset{{ (-1)}}{\check{h}}_{AC} \overset{{ (-2)}}{\check{h}}_{BD} - 2 \overset{{ (-2)}}{\check{h}}_{AC} \mathring{D}_B \overset{{ (0)}}{{{\check{h}}_{u}{}} }{}_{D}\right) \right) } \nonumber \\&\qquad \times d\mu _{\mathring{\gamma }}+ o(1) \,. \end{aligned}$$

The associated energy flux formula follows from (2.36) together with the equations just listed:

5.33 $$\begin{aligned}&\frac{d E_c[h,\mathscr {C}_{u,R}]}{du} = -\frac{1}{32 \pi } \int _{S_R} r^2 b^r(\partial _uh, h) \, d\mu _{{{\mathring{{\gamma }}}}} \nonumber \\&\quad ={ {\frac{R}{32 \pi } } \int _{S^2} \left( 4 { {{\mathring{{\gamma }}}}{}^{AB} \overset{{ (-2)}}{\check{h}}_{uA} \partial _{u} \overset{{ (0)}}{\check{h}}_{uB}} \right. }- {{\mathring{{\gamma }}}}^{AB}{{\mathring{{\gamma }}}}^{CD} \nonumber \\&\qquad \times {\left. \left( {\alpha ^2} \overset{{ (-1)}}{\check{h}}_{AC} \partial _{u} \overset{{ (-1)}}{\check{h}}_{BD} - 2 \partial _{u} \overset{{ (-1)}}{\check{h}}_{AC} \mathring{D}_B \overset{{ (0)}}{{{\check{h}}_{u}{}} }{}_{D}\right) \right) d^2 \mu _{{\mathring{{\gamma }}}}} \nonumber \\&\qquad - {\frac{1}{32 \pi } } \int _{S^2} \left( { - 6 {{\mathring{{\gamma }}}}^{AB} \overset{{ (-3)}}{{{\check{h}}_{u}{}} }{}_{A} \partial _{u}\overset{{ (0)}}{{{\check{h}}_{u}{}} }{}_{B}} \right. \nonumber \\&\qquad + {{\mathring{{\gamma }}}}^{AB}{{\mathring{{\gamma }}}}^{CD} \partial _{u} \overset{{ (-1)}}{\check{h}}_{AC} \partial _{u} \overset{{ (-1)}}{\check{h}}_{BD} \nonumber \\&\qquad {\left. + {{\mathring{{\gamma }}}}^{AB}{{\mathring{{\gamma }}}}^{CD} \left( {\alpha ^2} \overset{{ (-1)}}{\check{h}}_{AC} \partial _{u}\overset{{ (-2)}}{\check{h}}_{BD} - 2 \partial _{u} \overset{{ (-2)}}{\check{h}}_{AC} \mathring{D}_B \overset{{ (0)}}{{{\check{h}}_{u}{}} }{}_D\right) \right) } \nonumber \\&\qquad \times d \mu _{{\mathring{{\gamma }}}}+ o(1) \,. \end{aligned}$$

Let us first analyse the convergence, as *R* tends to infinity, of the volume integral in (5.32).

Consider the part of the boundary integral in (5.32) which diverges linearly with *R*; up to a multiplicative coefficient $R/(64\pi )$ this term equals

5.34 $$\begin{aligned}&\int _{S^2} \left( { 4{{\mathring{{\gamma }}}}^{AB} \overset{{ (0)}}{\check{h}}_{uA} \overset{{ (-2)}}{\check{h}}_{uB} } -{{\mathring{{\gamma }}}}^{AB}{{\mathring{{\gamma }}}}^{CD} (\alpha ^2 \overset{{ (-1)}}{\check{h}}_{AC} \overset{{ (-1)}}{\check{h}}_{BD}\right. \nonumber \\&\quad \left. - 2 \overset{{ (-1)}}{\check{h}}_{AC} \mathring{D}_B \overset{{ (0)}}{{{\check{h}}_{u}{}} }{}_{D}) \right) \, d \mu _{{\mathring{{\gamma }}}} \,. \end{aligned}$$

Using (4.23), the first term in (5.34) can be rewritten as

5.35 $$\begin{aligned}&- 4 \int _{S^2} { {{\mathring{{\gamma }}}}^{AB} \overset{{ (0)}}{\check{h}}{}_{uA} \overset{{ (-2)}}{\check{h}}{}_{uB}} \, d \mu _{{\mathring{{\gamma }}}}\nonumber \\&\quad = - 2 \int _{S^2} {{\mathring{{\gamma }}}}^{AB} \overset{{ (0)}}{\check{h}}{}_{uA} \mathring{D}^C \overset{{ (-1)}}{\check{h}}_{BC} \, d \mu _{{\mathring{{\gamma }}}} \nonumber \\&\quad = 2 \int _{S^2} {{\mathring{{\gamma }}}}^{AB} \mathring{D}^C \overset{{ (0)}}{\check{h}}{}_{uA} \overset{{ (-1)}}{\check{h}}_{BC} \, d \mu _{{\mathring{{\gamma }}}} \,, \end{aligned}$$

which cancels-out the last term in (5.34).

Incidentally, from (4.30) and the symmetries of $\overset{{ (-1)}}{\check{h}}_{AC}$ we obtain

5.36 $$\begin{aligned}&- 2\int _{S^2} {{\mathring{{\gamma }}}}^{AB}{{\mathring{{\gamma }}}}^{CD} \overset{{ (-1)}}{\check{h}}_{AC} \mathring{D}_B \overset{{ (0)}}{{{\check{h}}_{u}{}} }{}_{D} \, d \mu _{{\mathring{{\gamma }}}} \nonumber \\&\quad = - \int _{S^2} {{\mathring{{\gamma }}}}^{AB}{{\mathring{{\gamma }}}}^{CD} \overset{{ (-1)}}{\check{h}}_{AC} ( \mathring{D}_B \overset{{ (0)}}{\check{h}}_D + \mathring{D}_D { \overset{{ (0)}}{\check{h}}_{uB}}\nonumber \\&\qquad - \mathring{D}_E \overset{{ (0)}}{\check{h}}{}^E {{\mathring{{\gamma }}}}_{BD} ) d \mu _{\mathring{\gamma }} \nonumber \\&\quad = \alpha ^2 \int _{S^2} {{\mathring{{\gamma }}}}^{AB}{{\mathring{{\gamma }}}}^{CD} \overset{{ (-1)}}{\check{h}}_{AC} \overset{{ (-1)}}{\check{h}}_{BD} \, d \mu _{\mathring{\gamma }} \,, \end{aligned}$$

so all three terms in (5.34) are equal up to signs. Thus (5.32) can be rewritten as

5.37 $$\begin{aligned}&E_c[h,\mathscr {C}_{u,R}] \nonumber \\&\quad = \frac{1}{64 \pi } \int _{\mathscr {C}_{u,R}} {{g}}^{BE } {{g}}^{FC} \big (\partial _u h _{BC } \partial _{ r } h_{EF } - h_{BC } \partial _{ r } \partial _u h_{EF } \big )\, d\mu _{\mathscr {C}}\nonumber \\&\qquad - {\frac{\alpha ^2 R}{64 \pi } } \int _{S^2} {{\mathring{{\gamma }}}}^{AB}{{\mathring{{\gamma }}}}^{CD}\overset{{ (-1)}}{\check{h}}_{AC} \overset{{ (-1)}}{\check{h}}_{BD} \, d\mu _{{{\mathring{{\gamma }}}}} \nonumber \\&\qquad - {\frac{1}{{ 64} \pi } } \int _{S^2} {{\mathring{{\gamma }}}}^{ AB} \Big ( {{\mathring{{\gamma }}}}^{ CD} \overset{{ (-1)}}{\check{h}}_{AC} \partial _{u} \overset{{ (-1)}}{\check{h}}_{BD} - { 6 \overset{{ (0)}}{\check{h}}{}_{uA} \overset{{ (-3)}}{\check{h}}{}_{uB}} \Big ) \nonumber \\&\qquad \times d\mu _{{{\mathring{{\gamma }}}}}+ o(1) \,. \end{aligned}$$

We finish this section by a remark concerning the vanishing of $\overset{{ (0)}}{\check{h}}_{AB}$. If this were not the case, the insertion of the expansion (4.26) into the volume integral in (5.32) would lead to a divergent leading-order behaviour:

5.38 $$\begin{aligned}&\int _{\mathscr {C}_{u,R}} {{g}}^{BE } {{g}}^{FC} \left( h_{BC } \partial _{ r } \partial _u h_{EF } - \partial _u h_{BC } \partial _{ r } h_{EF } \right) \, d\mu _{\mathscr {C}} \nonumber \\&\quad = R \int _{S^2} {{\mathring{{\gamma }}}}^{BE } {{\mathring{{\gamma }}}}^{FC} \left( \overset{{ (-1)}}{\check{h}}_{BC } \partial _{u } \overset{{ (0)}}{\check{h}}_{EF } - \partial _u \overset{{ (-1)}}{\check{h}}_{BC } \overset{{ (0)}}{\check{h}}_{EF } \right) d\mu _{{\mathring{{\gamma }}}} \nonumber \\&\qquad + 2\ln ( R) \int _{S^2} {{\mathring{{\gamma }}}}^{BE } {{\mathring{{\gamma }}}}^{FC} \left( \overset{{ (-2)}}{\check{h}}_{BC } \partial _{u } \overset{{ (0)}}{\check{h}}_{EF } - \partial _u \overset{{ (-2)}}{\check{h}}_{BC } \overset{{ (0)}}{\check{h}}_{EF } \right) \nonumber \\&\qquad \times d\mu _{{\mathring{{\gamma }}}} + O (1) \,. \end{aligned}$$

Further, the boundary term in the energy would diverge as $R^2$. As already pointed out, this provides the justification while it is natural to choose a gauge in which $\overset{{ (0)}}{\check{h}}_{AB}$ vanishes.

#### Energy-loss revisited

In this section we rederive the energy-loss formula by a somewhat more direct calculation. For this we integrate over $\mathscr {C}_{u,R}$ the identity

5.39 $$\begin{aligned} \partial _u \omega ^u = -\partial _i \omega ^i \end{aligned}$$

to obtain

5.40 $$\begin{aligned} \frac{d}{du} \int _{\mathscr {C}_{u,R}} \omega ^u r^2 dr \,d\mu _{{\mathring{{\gamma }}}}= & {} -\int _{S_{u,R}} \omega ^r r^2 \,d\mu _{{\mathring{{\gamma }}}}\nonumber \\&+ \lim _{\epsilon \rightarrow 0} \int _{S_{u,\epsilon }} \omega ^r r^2 \,d\mu _{{\mathring{{\gamma }}}}\,. \end{aligned}$$

One way to guarantee the vanishing of the last integral in (5.40) is to choose a gauge so that

5.41 $$\begin{aligned} \partial _r h_{AB}|_{r=0} = 0 \,. \end{aligned}$$

For simplicity this gauge choice will be made in the rest of this section.

From (2.167) and (5.28) this is equivalent to

5.42 $$\begin{aligned}&\frac{1}{2} \frac{d}{du} \int _{\mathscr {C}_{u,R}} g^{AB}g^{CD}\left( \delta _2 g_ {AC}\partial _{{r}} \delta _{1}g_{BD}-\delta _1 g_ {AC}\partial _{{r}} \delta _{2}g_{BD}\right) r^2\nonumber \\&\qquad \times dr \,d\mu _{{\mathring{{\gamma }}}}\nonumber \\&\quad = \displaystyle -\int _{S_{u,R}} \left[ r \left[ 2 {\mathring{{\gamma }}}^{AB} \left( \delta _1 \overset{{ (2)}}{g}_{u A} \delta _2 \overset{{ (0)}}{g}_{u B} - \delta _2 \overset{{ (2)}}{g}_{u A} \delta _1 \overset{{ (0)}}{g}_{u B} \right) \right. \right. \nonumber \\&\qquad \left. + {\mathring{{\gamma }}}^{AC} {\mathring{{\gamma }}}^{BD} \left( \delta _1\overset{{ (1)}}{g}_{AB} \mathring{D}_C\delta _2 \overset{{ (2)}}{g}_{u D} - \delta _2\overset{{ (1)}}{g}_{AB} \mathring{D}_C\delta _1 \overset{{ (2)}}{g}_{u D} \right) \right] \nonumber \\&\qquad +3 {\mathring{{\gamma }}}^{AB} \left( \delta _1\overset{{ (2)}}{g}_{u A} \delta _2\overset{{ (-1)}}{g}_{u B} -\delta _2\overset{{ (2)}}{g}_{u A} \delta _1\overset{{ (-1)}}{g}_{u B} \right) \nonumber \\&\qquad + {\mathring{{\gamma }}}^{AC} {\mathring{{\gamma }}}^{BD} \left( \frac{1}{2} \left( \delta _2\overset{{ (1)}}{g}_{AB} \partial _{u} \delta _1\overset{{ (1)}}{g}_{CD} - \delta _1\overset{{ (1)}}{g}_{AB} \partial _{u} \delta _2\overset{{ (1)}}{g}_{CD}\right) \right. \nonumber \\&\qquad + \frac{\alpha ^2}{2 } \left( \delta _2\overset{{ (0)}}{g}_{AB} \delta _1\overset{{ (1)}}{g}_{CD} - \delta _1\overset{{ (0)}}{g}_{AB} \delta _2\overset{{ (1)}}{g}_{CD} \right) \nonumber \\&\qquad \left. \left. + \delta _1\overset{{ (0)}}{g}_{AB} \mathring{D}_C\delta _2\overset{{ (2)}}{g}_{u D} - \delta _2\overset{{ (0)}}{g}_{AB} \mathring{D}_C\delta _1\overset{{ (2)}}{g}_{u D} \right) \right] \,d\mu _{{\mathring{{\gamma }}}}\nonumber \\&\qquad + o(1) \,. \end{aligned}$$

Letting $$\delta _1g_{\mu \nu }= h_{\mu \nu }$$, $$\delta _2g_{\mu \nu }= \partial _u h_{\mu \nu }$$, using

$$\begin{aligned} \overset{{ (0)}}{\check{h}}_{AB}\equiv 0 \equiv \overset{{ (-2)}}{\check{h}}_{AB} \end{aligned}$$

(similarly for the *u*-derivatives), and keeping in mind that ${\breve{h}}{:}{=} r^{-1} h$ and $\check{h}=r^{-2} h$, this becomes

5.43 $$\begin{aligned}&\frac{1}{2} \frac{d}{du} \int _{\mathscr {C}_{u,R}} {\mathring{{\gamma }}}^{AB}{\mathring{{\gamma }}}^{CD}\left( \partial _u {\breve{h}}_ {AC}\partial _{{r}} {\breve{h}}_{BD}-{\breve{h}}_ {AC}\partial _{{r}} \partial _u {\breve{h}}_{BD}\right) dr \,d\mu _{{\mathring{{\gamma }}}}\nonumber \\&\quad = \displaystyle -\int _{S_{u,R}} \left[ r\bigg [ 2 {\mathring{{\gamma }}}^{AB} \left( \overset{{ (0)}}{\check{h}}_{u A} \partial _u \overset{{ (-2)}}{\check{h}}_{u B} - \partial _u \overset{{ (0)}}{\check{h}}_{u A} \overset{{ (-2)}}{\check{h}}_{u B} \right) \right. \nonumber \\&\qquad + {\mathring{{\gamma }}}^{AC} {\mathring{{\gamma }}}^{BD} \left( \overset{{ (-1)}}{\check{h}}_{AB} \mathring{D}_C\partial _u \overset{{ (0)}}{\check{h}}_{u D} - \partial _u \overset{{ (-1)}}{\check{h}}_{AB} \mathring{D}_C\overset{{ (0)}}{\check{h}}_{u D} \right) \bigg ] \nonumber \\&\qquad +3 {\mathring{{\gamma }}}^{AB} \left( \overset{{ (0)}}{\check{h}}_{u A} \partial _u \overset{{ (-3)}}{\check{h}}_{u B} - \partial _u \overset{{ (0)}}{\check{h}}_{u A} \overset{{ (-3)}}{\check{h}}_{u B} \right) \nonumber \\&\qquad \left. + \frac{1}{2} {\mathring{{\gamma }}}^{AC} {\mathring{{\gamma }}}^{BD} \left( \partial _u \overset{{ (-1)}}{\check{h}}_{AB} \partial _{u} \overset{{ (-1)}}{\check{h}}_{CD} - \overset{{ (-1)}}{\check{h}}_{AB} \partial ^2_{u} \overset{{ (-1)}}{\check{h}}_{CD} \right) \right] \,\nonumber \\&\qquad \times d\mu _{{\mathring{{\gamma }}}}+ o(1) \,. \end{aligned}$$

In order to obtain ${d E_c[h,\mathscr {C}_{u,R}]}/{du}$ from this formula, we analyse the surface terms in the definition of canonical energy (5.32):

5.44 $$\begin{aligned} B_{E_{c}}&{:}{=}R \int _{S^2} \left( 2 {{\mathring{{\gamma }}}}^{AB} \overset{{ (0)}}{{{\check{h}}_{u}{}} }{}_{A} \overset{{ (-2)}}{{{\check{h}}_{u}{}} }{}_{B} -\frac{1}{2}{{\mathring{{\gamma }}}}^{AB}{{\mathring{{\gamma }}}}^{CD}\right. \nonumber \\&\times \left. \big ( \alpha ^2 \overset{{ (-1)}}{\check{h}}_{AC} \overset{{ (-1)}}{\check{h}}_{BD} - 2 \overset{{ (-1)}}{\check{h}}_{AC} \mathring{D}_B \overset{{ (0)}}{\check{h}}_{BD} \big ) \right) d \mu _{{\mathring{{\gamma }}}} \nonumber \\&+ \int _{S^2} \left( 3 {{\mathring{{\gamma }}}}^{AB} \overset{{ (0)}}{{{\check{h}}_{u}{}} }{}_{A} \overset{{ (-3)}}{{{\check{h}}_{u}{}} }{}_{B} -\frac{1}{2}{{\mathring{{\gamma }}}}^{AB}{{\mathring{{\gamma }}}}^{CD}\right. \nonumber \\&\times \left. \big ( \overset{{ (-1)}}{\check{h}}_{AC} \partial _{u}\overset{{ (-1)}}{\check{h}}_{BD} \big ) \right) d \mu _{{\mathring{{\gamma }}}} \nonumber \\&+ o(1) \,. \end{aligned}$$

Explicitly computing $$ {d{B}_{E_c}}/{du}$$, adding both sides to (5.43), after multiplying by $$1/(32 \pi )$$ we recover the mass loss formula (5.33), keeping in mind that $$\overset{{ (-2)}}{\check{h}}_{AB}=0 $$ when no logarithms occur.

In the case $\varLambda =0$ things simplify as then $\alpha =\overset{{ (0)}}{\check{h}}_{u B} =0$. One can integrate by parts in the first line above so that the left-hand side of (5.43) reads instead

5.45 $$\begin{aligned}&\frac{d}{du} \left( \int _{\mathscr {C}_{u,R}} {\mathring{{\gamma }}}^{AB}{\mathring{{\gamma }}}^{CD} \partial _u {\breve{h}}_ {AC}\partial _{{r}} {\breve{h}}_{BD} dr \,d\mu _{{\mathring{{\gamma }}}}\right. \nonumber \\&\left. - \frac{1}{2} \int _{S_{u,R}} {\mathring{{\gamma }}}^{AB}{\mathring{{\gamma }}}^{CD} {\breve{h}}_ {AC} \partial _u {\breve{h}}_{BD} \,d\mu _{{\mathring{{\gamma }}}}\right) \,. \end{aligned}$$

From (5.43) one obtains

5.46 $$\begin{aligned}&\frac{d}{du} \left( \int _{\mathscr {C}_{u,R}} {\mathring{{\gamma }}}^{AB}{\mathring{{\gamma }}}^{CD} \partial _u {\breve{h}}_ {AC}\partial _{{r}} {\breve{h}}_{BD} dr \,d\mu _{{\mathring{{\gamma }}}}\right. \nonumber \\&\qquad \left. - \frac{1}{2} \int _{S_{u,R}} {\mathring{{\gamma }}}^{AB}{\mathring{{\gamma }}}^{CD} {\breve{h}}_ {AC} \partial _u {\breve{h}}_{BD} \,d\mu _{{\mathring{{\gamma }}}}\right) \nonumber \\&\quad = \displaystyle - \frac{1}{2}\int _{S_{u,R}} \Big [ {\mathring{{\gamma }}}^{AC} {\mathring{{\gamma }}}^{BD} \big ( \partial _u \overset{{ (-1)}}{\check{h}}_{AB} \partial _{u} \overset{{ (-1)}}{\check{h}}_{CD}\nonumber \\&\qquad - \overset{{ (-1)}}{\check{h}}_{AB} \partial ^2_{u} \overset{{ (-1)}}{\check{h}}_{CD} \big ) \Big ] \,d\mu _{{\mathring{{\gamma }}}}+ o(1) \,. \end{aligned}$$

Equivalently

5.47 $$\begin{aligned}&\frac{d}{du} \left( \int _{\mathscr {C}_{u,R}} {\mathring{{\gamma }}}^{AB}{\mathring{{\gamma }}}^{CD} \partial _u {\breve{h}}_ {AC}\partial _{{r}} {\breve{h}}_{BD} dr \,d\mu _{{\mathring{{\gamma }}}}\right. \nonumber \\&\qquad \left. - \int _{S_{u,R}} {\mathring{{\gamma }}}^{AB}{\mathring{{\gamma }}}^{CD} {\breve{h}}_ {AC} \partial _u {\breve{h}}_{BD} \,d\mu _{{\mathring{{\gamma }}}}\right) \nonumber \\&\quad = -\int _{S_{u,R}} {\mathring{{\gamma }}}^{AC} {\mathring{{\gamma }}}^{BD} \partial _u \overset{{ (-1)}}{\check{h}}_{AB} \partial _{u} \overset{{ (-1)}}{\check{h}}_{CD} \,d\mu _{{\mathring{{\gamma }}}}+ o(1) \,, \nonumber \\ \end{aligned}$$

or

5.48 $$\begin{aligned}&\frac{d}{du} \left( \int _{\mathscr {C}_{u,R}} {\mathring{{\gamma }}}^{AB}{\mathring{{\gamma }}}^{CD}\left( \partial _u {\breve{h}}_ {AC}\partial _{{r}} {\breve{h}}_{BD}-{\breve{h}}_ {AC}\partial _{{r}} \partial _u {\breve{h}}_{BD}\right) dr \,d\mu _{{\mathring{{\gamma }}}}\right. \nonumber \\&\qquad \left. - \int _{S_{u,R}} {\mathring{{\gamma }}}^{AB}{\mathring{{\gamma }}}^{CD} {\breve{h}}_ {AC} \partial _u {\breve{h}}_{BD} \,d\mu _{{\mathring{{\gamma }}}}\right) \nonumber \\&\quad = -2 \int _{S_{u,R}} {\mathring{{\gamma }}}^{AC} {\mathring{{\gamma }}}^{BD} \partial _u \overset{{ (-1)}}{\check{h}}_{AB} \partial _{u} \overset{{ (-1)}}{\check{h}}_{CD} \,d\mu _{{\mathring{{\gamma }}}}+ o(1) \,.\nonumber \\ \end{aligned}$$

Passing with *R* to infinity, after multiplying by $1/(64 \pi )$ the right-hand side becomes (up to a multiplicative factor) the right-hand side of the familiar Trautman–Bondi mass loss formula. Hence the expression which is differentiated at the left-hand side must be the Trautman–Bondi energy, up to the addition of a time-independent functional of the fields.

Now, it is known that there are no such functionals in the nonlinear theory which are gauge-independent and which vanish when $h_{\mu \nu }$ vanishes by [17], and it is clear that the argument there carries over to the linearised theory. However, gauge-independence of the functional being differentiated in (5.48), namely

5.49 $$\begin{aligned}&\int _{\mathscr {C}_{u }} {\mathring{{\gamma }}}^{AB}{\mathring{{\gamma }}}^{CD}\left( \partial _u {\breve{h}}_ {AC}\partial _{{r}} {\breve{h}}_{BD}-{\breve{h}}_ {AC}\partial _{{r}} \partial _u {\breve{h}}_{BD}\right) dr \,d\mu _{{\mathring{{\gamma }}}}\nonumber \\&\quad - \lim _{R\rightarrow \infty } \int _{S_{u,R}} {\mathring{{\gamma }}}^{AB}{\mathring{{\gamma }}}^{CD} {\breve{h}}_ {AC} \partial _u {\breve{h}}_{BD} \,d\mu _{{\mathring{{\gamma }}}}\,, \end{aligned}$$

is not clear at this stage. In Sect. 5.6 below we settle the issue by providing a direct proof of the equality of $\hat{E}_c$ with half of the quadratisation of the Trautman–Bondi mass. When $\varLambda$ vanishes this could have been anticipated by the results of [15], where it is shown that the canonical energy for the nonlinear field is the Trautman–Bondi mass. However, such statements involve a careful choice of boundary terms so that the correspondence is not automatic. And no such statement for $\varLambda >0$ has been established so far in any case.

### Renormalised energy

Equation (5.33) shows that the divergent term in $E_c$ has a dynamics of its own, evolving separately from the remaining part of the canonical energy. It is therefore natural to introduce a *renormalised canonical energy*, say $\hat{E}_c[h,\mathscr {C}_{u,R }]$, by removing the divergent term in (5.37). After having done this, we can pass to the limit $R\rightarrow \infty$ to obtain:

5.50 $$\begin{aligned}&\hat{E}_c[h,\mathscr {C}_{u }] \nonumber \\&\quad : = \frac{1}{64 \pi } \int _{\mathscr {C}_{u }} {{g}}^{BE } {{g}}^{FC} \big ( \partial _u h_{BC } \partial _{ r } h_{EF } - h_{BC } \partial _{ r } \partial _u h_{EF } \big )\,r^2 \,\nonumber \\&\qquad \times dr \sin \theta \, d\theta \, d\varphi \nonumber \\&\qquad - {\frac{1}{64 \pi } } \int _{S^2} {{\mathring{{\gamma }}}}^{AB} \Big ({{\mathring{{\gamma }}}}^{CD}\overset{{ (-1)}}{\check{h}}_{AC} \partial _{u} \overset{{ (-1)}}{\check{h}}_{BD} - { 6 \overset{{ (0)}}{\check{h}}{}_{uA} \overset{{ (-3)}}{\check{h}}{}_{uB}} \Big ) \,\nonumber \\&\qquad \times \sin \theta \, d\theta \, d\varphi \,. \end{aligned}$$

This is our first main result here, and is our proposal how to calculate the total energy contained in a light cone of a weak gravitational wave on a de Sitter background.

The flux equation for the renormalised energy $\hat{E}_c$ coincides with the one obtained by dropping the term linear in *R* in (5.33) and passing again to the limit $R\rightarrow \infty$:

5.51 $$\begin{aligned}&\frac{d \hat{E}_c[h,\mathscr {C}_{u,R}]}{du} = - {\frac{1}{32 \pi } } \times \nonumber \\&\quad \int _{S^2} {{\mathring{{\gamma }}}}^{AB}\Big ({{\mathring{{\gamma }}}}^{CD} \partial _{u} \overset{{ (-1)}}{\check{h}}_{AC} \partial _{u} \overset{{ (-1)}}{\check{h}}_{BD} - { 6 \overset{{ (-3)}}{\check{h}}{}_{uA}} \partial _u \overset{{ (0)}}{\check{h}}{}_{uB} \Big ) \, \nonumber \\&\qquad \times \sin \theta \, d\theta \, d\varphi . \end{aligned}$$

This is our key new formula. When $\varLambda =0$ we recover the weak-field version of the usual Trautman–Bondi mass loss formula since, as already pointed out, $\overset{{ (0)}}{\check{h}}_{uA}\equiv 0$ in the asymptotically Minkowskian case. Hence the last term in (5.51) shows how the cosmological constant affects the flux of energy emitted by a gravitating astrophysical system.

### Energy and gauge transformations

We have seen by general considerations that the energy integral is invariant under gauge transformations, up to boundary terms. It is instructive to rederive this result for the residual gauge transformations by a direct calculation. As a byproduct we obtain the explicit form of the boundary terms arising.

We focus attention on the volume part of the energy integral:

5.52 $$\begin{aligned} E_{{V}}[h](u):= & {} \frac{1}{64 \pi } \int _{\mathscr {C}_u} {{g}}^{BE } {{g}}^{FC} \big ( \partial _u h_{BC } \partial _{ r } h_{EF }\nonumber \\&- h_{BC } \partial _{ r } \partial _u h_{EF } \big )\, d\mu _{\mathscr {C}} \,. \end{aligned}$$

It is convenient to define a new field

5.53 $$\begin{aligned} {\breve{h}}_{\mu \nu } {:}{=} r^{-1} h_{\mu \nu } \equiv r \check{h}_{\mu \nu } \,. \end{aligned}$$

In terms of ${\breve{h}}$ the integral $E_{{V}}$ takes the form

5.54 $$\begin{aligned}&E_{{V}}[h](u) \nonumber \\&\quad = \frac{1}{64 \pi } \int _{\mathscr {C}_u} {{\mathring{{\gamma }}}}^{BE } {{\mathring{{\gamma }}}}^{FC} \big ( \partial _u {\breve{h}}_{BC } \partial _{ r } {\breve{h}}_{EF } - {\breve{h}}_{BC } \partial _{ r } \partial _u {\breve{h}}_{EF } \big )\nonumber \\&\qquad dr \,d\mu _{{\mathring{{\gamma }}}}\,. \end{aligned}$$

Under asymptotic symmetries we have (cf. (3.43))

5.55 $$\begin{aligned} {\breve{h}}_{AB}&\mapsto {\breve{h}}_{AB} + r \big ( \underbrace{\mathring{D}_{A} \xi _{B} + \mathring{D}_{B} \xi _{A}- \mathring{\gamma }_{AB}\mathring{D}^{C} \xi _{C} }_{0} \big ) \nonumber \\&\quad + \big ( \mathring{\gamma }_{AB} \varDelta _{\mathring{\gamma }} -2 \mathring{D}_{A}\mathring{D} _{B} \big ) \xi ^{u} \,, \end{aligned}$$

where the term linear in *r* vanishes since ${\xi }^A(u,\cdot )$ is a conformal Killing vector of $S^2$. Whence

5.56 $$\begin{aligned} \partial _u{\breve{h}}_{AB}&\mapsto \partial _u{\breve{h}}_{AB} + \big ( \mathring{\gamma }_{AB} \varDelta _{\mathring{\gamma }} -2 \mathring{D}_{A}\mathring{D} _{B} \big )\partial _u\xi ^{u}(u, x^{A}) \,, \end{aligned}$$

5.57 $$\begin{aligned} \partial _r{\breve{h}}_{AB}&\mapsto \partial _r{\breve{h}}_{AB} \,. \end{aligned}$$

Now, for each *u* the function $\partial _u{\xi }^u$ is a linear combination of $\ell =0$ and $\ell =1$ spherical harmonics (see Appendix A), which implies that the terms involving $\partial _u{\xi }^u$ in $\partial _u {\breve{h}}_{AB}$ vanish. We conclude that both $\partial _u h_{AB} \equiv r \partial _u {\breve{h}}_{AB}$ and $\partial _r(r^{-1} h_{AB})\equiv \partial _r{\breve{h}}_{AB}$ are invariant under asymptotic symmetries.

Since the measure $d\mu _{{\mathring{{\gamma }}}}= \sqrt{\det {{\mathring{{\gamma }}}}}\, dx^2\wedge dx^3$ is *r*-independent, integration by parts in (5.52) gives

5.58 $$\begin{aligned} E_{{V}}[h](u)= & {} \frac{1}{32 \pi } \int _{\mathscr {C}_u} {{\mathring{{\gamma }}}}^{BE } {{\mathring{{\gamma }}}}^{FC} \partial _u {\breve{h}}_{BC } \partial _{ r } {\breve{h}}_{EF }\,dr \, d\mu _{{{\mathring{{\gamma }}}}} \nonumber \\&+\lim _{\epsilon \rightarrow 0}\frac{1}{64 \pi } \int _{r=\epsilon } {{\mathring{{\gamma }}}}^{BE } {{\mathring{{\gamma }}}}^{FC} {\breve{h}}_{BC } \partial _u {\breve{h}}_{EF } \,d\mu _{{\mathring{{\gamma }}}}\nonumber \\&- \lim _{R\rightarrow \infty }\frac{1}{64 \pi } \int _{r=R} {{\mathring{{\gamma }}}}^{BE } {{\mathring{{\gamma }}}}^{FC} {\breve{h}}_{BC } \partial _u {\breve{h}}_{EF } \,d\mu _{{\mathring{{\gamma }}}}\,, \nonumber \\ \end{aligned}$$

and note that the second line above does not vanish in a general Bondi gauge, since then both ${\breve{h}}_{AB}$ and $\partial _u{\breve{h}}_{AB}$ are of order one for small *r* by (5.55). So

5.59 $$\begin{aligned}&E_{{V}}[h](u) = \frac{1}{32 \pi } \int _{\mathscr {C}_u} {{\mathring{{\gamma }}}}^{BE } {{\mathring{{\gamma }}}}^{FC} \partial _u {\breve{h}}_{BC } \partial _{ r } {\breve{h}}_{EF }\,dr \, d\mu _{{{\mathring{{\gamma }}}}}\nonumber \\&\, +\frac{1}{64 \pi } \int _{S^2} {{\mathring{{\gamma }}}}^{BE } {{\mathring{{\gamma }}}}^{FC} \big ( ( {\breve{h}}_{BC } \partial _u {\breve{h}}_{EF })|_{r=0} - \overset{{ (0)}}{{\breve{h}}}_{BC } \partial _u \overset{{ (0)}}{{\breve{h}}}_{EF } \big ) \,d\mu _{{\mathring{{\gamma }}}}, \, \nonumber \\ \end{aligned}$$

where we assumed that the metric $h_{\mu \nu }$ has the usual asymptotic expansion for large *r* as considered elsewhere in this paper.

From what has been said the volume integral in (5.59) is invariant under residual gauge transformations, so that we have

5.60 $$\begin{aligned}&E_{{V}}[h](u) \mapsto E_{{V}}[h](u) \nonumber \\&\quad + \frac{1}{64 \pi } \int _{S^2} {{\mathring{{\gamma }}}}^{AB } {{\mathring{{\gamma }}}}^{CD} \big ( \partial _u \check{h}_{AC }|_{r=0} - \partial _u \overset{{ (-1)}}{\check{h}}_{AC } \big ) \nonumber \\&\qquad \times \big ( \mathring{\gamma }_{BD} \varDelta _{\mathring{\gamma }} -2 \mathring{D}_{B}\mathring{D} _{D} \big ) \xi ^{u} \,d\mu _{{\mathring{{\gamma }}}}\,. \end{aligned}$$

The second line in (5.60) vanishes when $\xi ^u$ is a linear combination of $\ell =0$ and $\ell =1$ spherical harmonics.

It easily follows that the canononical energy is invariant under such residual gauge transformations.

### $\hat{E}_c=\frac{1}{2}\delta ^2 m_{\mathrm {TB}}$

In this section we show by a direct calculation that our renormalised energy $\hat{E}_c$ coincides with the Trautman–Bondi mass of the linearised theory. As before we fix a light cone $\mathscr {C}_u$ and use the gauge (5.31) on $\mathscr {C}_u$.

We start with (5.13), which we repeat here for the convenience of the reader:

5.61 $$\begin{aligned}&\delta ^2 m_{\mathrm {TB}}(\mathscr {C}_{u,R}) = \frac{1}{16 \pi } \int _{\mathscr {C}_{u,R}} \Big ( r^4 {{\mathring{{\gamma }}}}^{AB}\partial _r \check{h}_{uA} \partial _r \check{h}_{uB}\nonumber \\&\quad { -4}(1- \varLambda r^2 ) \delta ^2 \beta \Big ) \, dr \, d\mu _{{{\mathring{{\gamma }}}}} \,.\nonumber \\ \end{aligned}$$

We integrate by parts on the $\delta ^2 \beta$ term and use the vacuum version of (5.3),

5.62 $$\begin{aligned} \partial _r \delta ^2\beta = \frac{r}{8}{{\mathring{{\gamma }}}}^{AC}{{\mathring{{\gamma }}}}^{BD} \partial _r \check{h}_{AB}\partial _r \check{h}_{CD} \,, \end{aligned}$$

to obtain

5.63 $$\begin{aligned} \delta ^2 m_{\mathrm {TB}}(\mathscr {C}_{u,R})= & {} \frac{1}{16 \pi } \int _{\mathscr {C}_{u,R}} \Bigg ( r^4 {{\mathring{{\gamma }}}}^{AB}\partial _r \check{h}_{u A}\partial _r \check{h}_{uB} \nonumber \\&{ +} \frac{3 r^2- {\varLambda r^4} }{6}{{\mathring{{\gamma }}}}^{AC}{{\mathring{{\gamma }}}}^{BD} \partial _r \check{h}_{AB}\partial _r \check{h}_{CD} \Bigg ) \, dr\, d\mu _{{{\mathring{{\gamma }}}}} \nonumber \\&{ -} \frac{1}{4 \pi }\int _{S_R} r \left( 1- \frac{\varLambda r^2}{3}\right) \delta ^2 \beta \, d\mu _{{{\mathring{{\gamma }}}}} \,. \end{aligned}$$

The first volume integral can be handled using (4.14),

5.64 $$\begin{aligned} \partial _r \left[ r^4 \partial _r(r^{-2} \delta g_{uA})\right] = r^2 \mathring{D}_E \left( {\mathring{{\gamma }}}^{EF}\partial _r \left( r^{-2}\delta g_{AF}\right) \right) \,. \end{aligned}$$

Equivalently,

5.65 $$\begin{aligned} \partial _r \left[ r^4 \partial _r \check{h}_{uA} \right] = r^{ 2} \partial _r \mathring{D}^{ F}\check{h}_{AF} \,. \end{aligned}$$

Hence

5.66 $$\begin{aligned}&\frac{1}{16 \pi } \int _{\mathscr {C}_{u,R}} r^4 {{\mathring{{\gamma }}}}^{AB}\partial _r \check{h}_{u A}\partial _r \check{h}_{uB} \, { d\mu _{{{\mathring{{\gamma }}}}} \, dr } \nonumber \\&\quad = \frac{1}{16 \pi } \int _{S_R} r^4 {{\mathring{{\gamma }}}}^{AB} \check{h}_{u A} \partial _r \check{h}_{uB}\, d\mu _{{{\mathring{{\gamma }}}}} \nonumber \\&\qquad - \frac{1}{16 \pi } \int _{\mathscr {C}_{u,R}} {{\mathring{{\gamma }}}}^{AB} \check{h}_{uA} \partial _r (r^4 \partial _r \check{h}_{u B}) \, { d\mu _{{{\mathring{{\gamma }}}}} \, dr } \nonumber \\&\quad = \frac{1}{16 \pi } \int _{S_R} r^4 {{\mathring{{\gamma }}}}^{AB} \check{h}_{u A} \partial _r \check{h}_{uB}\, d\mu _{{{\mathring{{\gamma }}}}} \nonumber \\&\qquad - \frac{1}{16 \pi } \int _{\mathscr {C}_{u,R}} r^{ 2}{{\mathring{{\gamma }}}}^{AB} \check{h}_{uA} \partial _r\mathring{D}^{ F}\check{h}_{BF} \, { d\mu _{{{\mathring{{\gamma }}}}} \, dr } \,. \end{aligned}$$

Equation (5.63) can thus be rewritten as

5.67 $$\begin{aligned}&\delta ^2 m_{\mathrm {TB}}(\mathscr {C}_{u,R}) \nonumber \\&\quad = - \frac{1}{16 \pi } \int _{\mathscr {C}_{u,R}} \Bigg ( r^{ 2}{{\mathring{{\gamma }}}}^{AB} \check{h}_{uA} \partial _r \mathring{D}^{ F}\check{h}_{BF} \nonumber \\&\qquad { -} \frac{3 r^2- {\varLambda r^4} }{{6}} {{\mathring{{\gamma }}}}^{AC}{{\mathring{{\gamma }}}}^{BD} \partial _r \check{h}_{AB}\partial _r \check{h}_{CD} \Bigg ) \, dr\, d\mu _{{{\mathring{{\gamma }}}}} \nonumber \\&\qquad + \frac{1}{16 \pi }\int _{S_R} \left( r^4 {{\mathring{{\gamma }}}}^{AB} \check{h}_{u A} \partial _r \check{h}_{uB} - 4 r \!\left( 1- \frac{\varLambda r^2}{3}\right) \delta ^2 \beta \right) \! d\mu _{{{\mathring{{\gamma }}}}}. \,\, \nonumber \\ \end{aligned}$$

Denoting by $E_V[h,\mathscr {C}_{u,R}]$ the volume term in (5.32) and

$$\begin{aligned} \breve{h}_{AB} {:}{=} r^{-1} h_{AB} \,, \end{aligned}$$

after another integration by parts we find

5.68 $$\begin{aligned} E_V[h,\mathscr {C}_{u,R}]&{:}{=} \frac{1}{64 \pi } \int _{\mathscr {C}_{u,R}} {{g}}^{BE } {{g}}^{FC} \big ( \partial _u h _{BC } \partial _{ r } h_{EF }\nonumber \\&\quad - h_{BC } \partial _{ r } \partial _u h_{EF } \big )\, d\mu _{\mathscr {C}} \nonumber \\&= \frac{1}{64 \pi } \int _{\mathscr {C}_{u,R}} {{\mathring{{\gamma }}}}^{BE } {{\mathring{{\gamma }}}}^{FC} \nonumber \\&\qquad \big ( \partial _u \breve{h} _{BC } \partial _{ r } \breve{h}_{EF } - \breve{h}_{BC } \partial _{ r } \partial _u \breve{h}_{EF } \big )\, { d\mu _{{{\mathring{{\gamma }}}}} \, dr }\nonumber \\&= - \frac{1}{32 \pi } \int _{\mathscr {C}_{u,R}} {{{\mathring{{\gamma }}}}}^{BE } {{{\mathring{{\gamma }}}}}^{FC}\nonumber \\&\quad \breve{h}_{BC } \partial _{ r } \partial _u \breve{h}_{EF } \, dr \, d\mu _{{{\mathring{{\gamma }}}}}\nonumber \\&\quad + \frac{1}{64 \pi } \int _{S_R} r^{-2} {{{\mathring{{\gamma }}}}}^{BE } {{\mathring{{\gamma }}}}^{FC} \partial _u h _{BC } h_{EF } \, d\mu _{{{\mathring{{\gamma }}}}} \,. \end{aligned}$$

Inserting the vacuum version of (4.25),

5.69 $$\begin{aligned}&\partial _r \partial _u \breve{h}_{EF} + \frac{\epsilon }{2r } \partial _r[ r^2 {N}^2 \partial _r \check{h}_{EF}] - TS \left[ \frac{1}{r } \mathring{D}_E\big ( \partial _r (r^2\check{h}_{uF}) \big )\right] \nonumber \\&\quad = 0 \,, \end{aligned}$$

into (5.68) we are led to

5.70 $$\begin{aligned}&E_V[h,\mathscr {C}_{u,R}] \nonumber \\&\quad = \frac{1}{32 \pi } \int _{\mathscr {C}_{u,R}} {{{\mathring{{\gamma }}}}}^{BE } {{{\mathring{{\gamma }}}}}^{FC} \check{h}_{BC }\nonumber \\&\qquad \times \left( \frac{\epsilon }{2} \partial _r[ r^2 {N}^2 \partial _r \check{h}_{EF}] - \mathring{D}_E\big ( \partial _r (r^2\check{h}_{uF}) \big ) \right) \, { d\mu _{{{\mathring{{\gamma }}}}} \, dr } \nonumber \\&\qquad + \frac{1}{64 \pi } \int _{S_R} r^{-2} {{{\mathring{{\gamma }}}}}^{BE } {{\mathring{{\gamma }}}}^{FC} \partial _u h _{BC } h_{EF } \, d\mu _{{{\mathring{{\gamma }}}}} \nonumber \\&\quad = - \frac{1}{32 \pi } \int _{\mathscr {C}_{u,R}} {{{\mathring{{\gamma }}}}}^{BE } {{{\mathring{{\gamma }}}}}^{FC}\nonumber \\&\qquad \times \left( \frac{\epsilon }{2 } r^2 {N}^2 \partial _r \check{h}_{BC } \partial _r \check{h}_{EF} + \check{h}_{BC } \mathring{D}_E\big ( \partial _r (r^2\check{h}_{uF}) \big ) \right) d\mu _{{{\mathring{{\gamma }}}}}\, dr \nonumber \\&\qquad + \frac{1}{64 \pi } \int _{S_R} {{{\mathring{{\gamma }}}}}^{BE } {{\mathring{{\gamma }}}}^{FC} h _{BC } \big ( \partial _u \check{h}_{EF } + {\epsilon } {N}^2 \partial _r \check{h}_{EF} \big ) \, d\mu _{{{\mathring{{\gamma }}}}} \,.\nonumber \\ \end{aligned}$$

The last integral in the before-last line can be integrated by parts over the angles to become, using (5.67) to pass from (5.71) to (5.72),

5.71 $$\begin{aligned}&-\frac{1}{32 \pi } \int _{\mathscr {C}_{u,R}} {{{\mathring{{\gamma }}}}}^{BE } {{{\mathring{{\gamma }}}}}^{FC} \check{h}_{BC } \mathring{D}_E\big ( \partial _r (r^2\check{h}_{uF}) \big ) \big ) \, d\mu _{{{\mathring{{\gamma }}}}}\, dr \nonumber \\&\quad =\frac{1}{32 \pi } \int _{\mathscr {C}_{u,R}} {{{\mathring{{\gamma }}}}}^{FC} \mathring{D}^B \check{h}_{BC } \partial _r (r^2\check{h}_{uF}) \, d\mu _{{{\mathring{{\gamma }}}}}\, dr \nonumber \\&\quad =- \frac{1}{32 \pi } \int _{\mathscr {C}_{u,R}} {{\mathring{{\gamma }}}}^{FC} r^2\check{h}_{uF} \partial _r \mathring{D}^B \check{h}_{BC } \, d\mu _{{{\mathring{{\gamma }}}}}\, dr \nonumber \\&\qquad + \frac{1}{32 \pi } \int _{S_R }r^2 {{{\mathring{{\gamma }}}}}^{FC} \check{h}_{uF} \mathring{D}^B \check{h}_{BC} \, d\mu _{{{\mathring{{\gamma }}}}} \end{aligned}$$

5.72 $$\begin{aligned}&\quad = \frac{1}{2} \delta ^2 m_{\mathrm {TB}}(\mathscr {C}_{u,R}) \nonumber \\&\qquad { -}\frac{1}{32 \pi } \int _{\mathscr {C}_{u,R}} \underbrace{\frac{3 r^2- {\varLambda r^4} }{6}}_{-\epsilon r^2 {N}^2/2} {{\mathring{{\gamma }}}}^{AC}{{\mathring{{\gamma }}}}^{BD} \partial _r \check{h}_{A B}\partial _r \check{h}_{C D} \, dr\, d\mu _{{{\mathring{{\gamma }}}}} \nonumber \\&\qquad - \frac{1}{32 \pi }\int _{S_R} \Bigg ( r^4 {{\mathring{{\gamma }}}}^{AB} \check{h}_{u A} \partial _r \check{h}_{uB} - 4r \left( 1- \frac{\varLambda r^2}{3}\right) \delta ^2 \beta \nonumber \\&\qquad - r^2 {{{\mathring{{\gamma }}}}}^{FC} \check{h}_{uF} \mathring{D}^B \check{h}_{BC} \Bigg ) \, d\mu _{{{\mathring{{\gamma }}}}} \,. \end{aligned}$$

Hence

5.73 $$\begin{aligned}&E_V[h,\mathscr {C}_{u,R}] = \frac{1}{2} \delta ^2 m_{\mathrm {TB}}(\mathscr {C}_{u,R})\nonumber \\&\quad - \frac{1}{64 \pi }\int _{S_R} \Bigg ( 2 r^4 {{\mathring{{\gamma }}}}^{AB} \check{h}_{u A} \partial _ r \check{h}_{uB} - 8 r \left( 1- \frac{\varLambda r^2}{3}\right) \delta ^2 \beta \nonumber \\&\quad - 2 r^2{{{\mathring{{\gamma }}}}}^{FC} \check{h}_{uF} \mathring{D}^B \check{h}_{BC} - {{{\mathring{{\gamma }}}}}^{BE } {{\mathring{{\gamma }}}}^{FC} h _{BC } \nonumber \\&\qquad \times \big ( \partial _u \check{h}_{EF } + {\epsilon } {N}^2 \partial _r \check{h}_{EF} \big ) \Bigg ) \, d\mu _{{{\mathring{{\gamma }}}}} \,. \end{aligned}$$

Now, using (2.124), (2.126), (2.138), (5.21), the total canonical energy equals

5.74 $$\begin{aligned} E_c [\mathscr {C}_{u,R},h]&{:}{=}\frac{1}{2} \left( \int _{\mathscr {C}_{u,R}} \omega ^\mu (h , {\mathcal {L}}_X h) \, dS_\mu \right. \nonumber \\&\left. - \int _{S_R} \tilde{{\pi }}^{ \alpha \beta [\mu } X^{{{\sigma }}]} h_{\alpha \beta } dS_{\sigma \mu } \right) \nonumber \\= & {} E_V[h,\mathscr {C}_{u,R}] - {\frac{1}{32 \pi } }\nonumber \\&\times \int _{S_R} P^{{ r} (\beta \gamma ) \delta (\epsilon \sigma ) }h_{\beta \gamma } { {\mathring{\nabla }}}_{ \delta } h_{\epsilon \sigma } \, r^2 d\mu _{{{\mathring{{\gamma }}}}} \nonumber \\= & {} E_V[h,\mathscr {C}_{u,R}] - {\frac{1}{32 \pi } } \int _{S_R} b^r (h,h) \, r^2 d\mu _{{{\mathring{{\gamma }}}}} \nonumber \\= & {} E_V[h,\mathscr {C}_{u,R}] \nonumber \\&- \frac{1}{32 \pi } \int _{S_R} \Big ( \frac{1}{2r^4} {\mathring{{\gamma }}}^{A C} {\mathring{{\gamma }}}^{B D}h_{C D} \nonumber \\&\times \big ( \epsilon {N}^2 \partial _{{r}}h_{AB} + \partial _{{u}}h_{AB} - 2 \mathring{D}_Ah_{u B} \big )\nonumber \\&+ h_{{u}A} {\mathring{{\gamma }}}^{A B} \underbrace{ \frac{1}{r^2} \left( \partial _{{r}}h_{{u}B} - \frac{2}{r}h_{{u}B} \right) }_{\partial _r \check{h}_{uB}} \Big ) \, r^2 d\mu _{{{\mathring{{\gamma }}}}} \nonumber \\= & {} \frac{1}{2} \delta ^2 m_{\mathrm {TB}}(\mathscr {C}_{u,R}) \nonumber \\&- \frac{1}{64 \pi }\int _{S_R} \Big ( 2 r^4 {{\mathring{{\gamma }}}}^{AB} \check{h}_{u A} \partial _r \check{h}_{uB} \nonumber \\&- 8 r \left( 1- \frac{\varLambda r^2}{3}\right) \delta ^2 \beta \nonumber \\&- 2 r^2{{{\mathring{{\gamma }}}}}^{FC} \check{h}_{uF} \mathring{D}^B \check{h}_{BC} - {{{\mathring{{\gamma }}}}}^{BE } {{\mathring{{\gamma }}}}^{FC} h _{BC } \nonumber \\&\times \big ( \partial _u \check{h}_{EF } + \epsilon {N}^2 \partial _r \check{h}_{EF} \big ) \Big ) \, d\mu _{{{\mathring{{\gamma }}}}}\nonumber \\&- \frac{1}{32 \pi } \int _{S_R} \left( \frac{1}{2 } {\mathring{{\gamma }}}^{A C} {\mathring{{\gamma }}}^{B D} { \check{h}} _{C D}\right. \nonumber \\&\times \big ( \epsilon {N}^2 \partial _{{r}}h_{AB} + \partial _{{u}}h_{AB} - 2 \mathring{D}_Ah_{u B} \big ) \nonumber \\&\left. + r^2 h_{{u}A} {\mathring{{\gamma }}}^{A B} \partial _{{r}}\check{h}_{{u}B} \right) \, d\mu _{{{\mathring{{\gamma }}}}} \nonumber \\= & {} \frac{1}{2} \delta ^2 m_{\mathrm {TB}}(\mathscr {C}_{u,R})\nonumber \\&- \frac{1}{64 \pi }\int _{S_R} \Big ( 4 r^4 {{\mathring{{\gamma }}}}^{AB} \check{h}_{u A} \partial _r \check{h}_{uB} \nonumber \\&- 8 r \underbrace{\left( 1- \frac{\varLambda r^2}{3}\right) }_{-\epsilon N^2} \delta ^2 \beta + {{{\mathring{{\gamma }}}}}^{BE } {{\mathring{{\gamma }}}}^{FC} \epsilon {N}^2 \nonumber \\&\times \big ( \check{h}_{BC } \partial _r h_{EF} -h _{BC } \partial _r \check{h}_{EF} \big ) \Big ) \, d\mu _{{{\mathring{{\gamma }}}}} \,. \end{aligned}$$

We emphasise that no asymptotic conditions have been used in the analysis in this section so far.

To continue we invoke the asymptotic conditions used in the preceding sections. From the expansion (5.5) of $\delta ^2\beta$ one obtains

5.75 $$\begin{aligned}&E_c [\mathscr {C}_{u,R},h] = \frac{1}{2} \delta ^2 m_{\mathrm {TB}}(\mathscr {C}_{u,R}) \nonumber \\&\quad - \frac{1}{64 \pi }\int _{S_R} \Bigg ( \frac{3 \alpha ^2}{2} r {{{\mathring{{\gamma }}}}}^{BE } {{\mathring{{\gamma }}}}^{FC} \overset{{ (-1)}}{\check{h}}_{BC } \overset{{ (-1)}}{\check{h}}_{EF} \nonumber \\&\qquad \qquad + 4 r^4 {{\mathring{{\gamma }}}}^{AB} \check{h}_{u A} \partial _r \check{h}_{uB} \Bigg ) \, d\mu _{{{\mathring{{\gamma }}}}}+ o (1) \, . \end{aligned}$$

Using (5.16) this becomes

5.76 $$\begin{aligned}&E_c [\mathscr {C}_{u,R},h] = \frac{1}{2} \delta ^2 m_{\mathrm {TB}}(\mathscr {C}_{u })+ \frac{R}{64 \pi } \int _{S_R} \nonumber \\&\quad \times \left( \!- \frac{3 \alpha ^2}{2} {{{\mathring{{\gamma }}}}}^{BE } {{\mathring{{\gamma }}}}^{FC} \overset{{ (-1)}}{\check{h}}_{BC } \overset{{ (-1)}}{\check{h}}_{EF} \!+\!8 {{\mathring{{\gamma }}}}^{AB} \overset{{ (0)}}{\check{h}}_{u A} \overset{{ (-2)}}{\check{h}}_{uB} \right) \nonumber \\&\quad \times d\mu _{{{\mathring{{\gamma }}}}} - \frac{\varLambda R}{128 \pi }\int _{S_R} {{\mathring{{\gamma }}}}^{AC}{{\mathring{{\gamma }}}}^{BD} \overset{{ (-1)}}{\check{h}}_{AB} \overset{{ (-1)}}{\check{h}}_{CD} \, d\mu _{{{\mathring{{\gamma }}}}} + o (1) \, .\nonumber \\ \end{aligned}$$

The last term in the second line can be integrated by parts as in (5.35)–(5.36) to obtain

5.77 $$\begin{aligned}&E_c [\mathscr {C}_{u,R},h] = \frac{1}{2} \delta ^2 m_{\mathrm {TB}}(\mathscr {C}_{u }) \nonumber \\&\quad - \frac{\alpha ^2 R}{64 \pi }\int _{S_R} {{\mathring{{\gamma }}}}^{AC}{{\mathring{{\gamma }}}}^{BD} \overset{{ (-1)}}{\check{h}}_{AB} \overset{{ (-1)}}{\check{h}}_{CD} \, d\mu _{{{\mathring{{\gamma }}}}} + o (1) \, . \end{aligned}$$

We see that the renormalised energy $\hat{E}_c$ coincides with one half of the quadratisation of the Trautman–Bondi mass.

### Energy in the asymptotically block-diagonal gauge

So far we have assumed a gauge where the leading order corrections to the metric are encoded in the off-diagonal components $h_{uA}$ of the metric perturbation. As such, the residual gauge freedom of Sect. 3.2 allows us to pass between two natural choices of asymptotic gauge:

5.78 $$\begin{aligned} \mathrm {(I)}&\overset{{ (0)}}{\check{h}}_{A B} \equiv 0 \, , \quad \overset{{ (0)}}{\check{h}}_{uA} \ne 0 \, , \end{aligned}$$

5.79 $$\begin{aligned} \mathrm {(II)}&\overset{{ (0)}}{\check{h}}_{A B} \ne 0 \, , \quad \overset{{ (0)}}{\check{h}}_{uA} \equiv 0 \, . \end{aligned}$$

Both choices are possible. However, as already emphasised in Sect. 3.2.1, when $\varLambda >0$ the asymptotic gauge $\overset{{ (0)}}{\check{h}}_{uA} \equiv 0$ cannot be attained simultaneously with $\overset{{ (0)}}{\check{h}}_{A B} \equiv 0$ for all *u* for general metric perturbations considered in this work.

We wish to revisit our analysis in the asymptotically block-diagonal gauge (I). Thus in the remainder of this section we assume

5.80 $$\begin{aligned} \delta \overset{{ (2)}}{g}_{A B}\ne & {} 0 \, , \end{aligned}$$

5.81 $$\begin{aligned} \delta \overset{{ (2)}}{g}_{u A }\equiv & {} 0 \, . \end{aligned}$$

#### The energy and its flux

Repeating the analysis of Sect. 4.1, one finds:

1. $\delta \overset{{ (2)}}{g}_{A B} \ne 0$ does not change the expansion (4.23) of $g_{u A}$. In particular $\delta \overset{{ (1)}}{g}_{u A}$ remains related with the log terms in the asymptotic expansion of $g_{AB}$.
2. When $\delta \overset{{ (2)}}{g}_{A B} \ne 0$, Eq. (4.36) becomes
  5.82 $$\begin{aligned} \delta V= & {} \frac{1}{2} \mathring{D}^A \big (\int _0^r \left( \mathring{D}^B \check{h}_{AB} -\frac{1}{r^2 } \big [ \partial _r (r^4\check{h}_{uA })\big ] \big ) \big |_{r=\rho } d\rho \right) \nonumber \\= & {} \mathring{D}^{A} \left( \frac{1}{2} r \mathring{D}^{B} \overset{{ (0)}}{\check{h}}_{AB } + {\hat{\nu } }_A + \frac{1}{2 r} \overset{{ (-3)}}{\check{h}}_{uA } + o(1/r) \right) \,. \end{aligned}$$
  5.83 $$\begin{aligned} {\hat{\nu }}_A= & {} \lim _{r \rightarrow \infty } \left( \frac{1}{2}\int _0^r \big ( \mathring{D}^B \check{h}_{AB} -\frac{1}{r^2 } \big [ \partial _r (r^4\check{h}_{uA })\big ] \big ) \big |_{r=\rho } d\rho \right. \nonumber \\&\left. -\frac{r}{2} \mathring{D}^{B} \overset{{ (0)}}{\check{h}}_{AB} \right) \!. \, \end{aligned}$$
  This differs from (4.37), but does not affect the mass because of the divergence structure of the right-hand side of (5.825.83).

The asymptotics of the linearised metric perturbations reads

5.84 $$\begin{aligned} \delta g_{u r}\equiv & {} 0 \ \, \equiv \ \, \delta g_{r A} \ \, \equiv \ \,\delta g_{rr} \,, \end{aligned}$$

5.85 $$\begin{aligned} \delta g_{AB}= & {} \delta \overset{{ (2)}}{g}_{A B}r_{{}}^2 + \delta \overset{{ (1)}}{g}_{A B} r_{{}}+ o(1) \,,\end{aligned}$$

5.86 $$\begin{aligned} \delta g_{u A}= & {} \delta \overset{{ (0)}}{g}_{u A} + o(1) \,, \end{aligned}$$

5.87 $$\begin{aligned} \delta g_{u u}= & {} \delta \overset{{ (0)}}{g}_{u u} +\frac{\delta \overset{{ (-1)}}{g}_{u u}}{r_{{}}} + o(r_{{}}^{-1}) \,. \end{aligned}$$

Let us denote by $\hat{E}_{c,II}$ the renormalised canonical energy in the block-diagonal gauge. To determine $\hat{E}_{c,II}$ we use (5.74) with the asymptotically block-diagonal boundary conditions. One checks that the asymptotic behaviour (5.5) of $\delta ^2 \beta$ remains unchanged at the order needed; the analysis of the remaining terms in (5.74) is likewise straightforward, leading to

5.88 $$\begin{aligned}&\hat{E}_{c,II} = \left( \frac{1}{2} \delta ^2 m_{\mathrm {TB}}(\mathscr {C}_{u }) -\frac{1}{16 \pi } \right. \nonumber \\&\quad \left. \times \int _{S^2} {{\mathring{{\gamma }}}}^{AC }{{\mathring{{\gamma }}}}^{BD }\left( \frac{\varLambda }{3} \overset{{ (-3)}}{\check{h}}_{AB} \overset{{ (0)}}{\check{h}}_{CD} -\overset{{ (-1)}}{\check{h}}_{AB} \overset{{ (0)}}{\check{h}}_{CD}\right) \, d \mu _{{\mathring{{\gamma }}}} \right) .\nonumber \\ \end{aligned}$$

In order to make clear the comparison with our previous calculations, let us denote by $\hat{E}_{c,I}$ the renormalised energy $\hat{E}_c$ calculated in the asymptotically off-diagonal gauge (I):

5.89 $$\begin{aligned} \hat{E}_{c,I} \equiv \hat{E}_{c} \,. \end{aligned}$$

Now, $\delta ^2 \overset{{ (0)}}{V}$ is gauge-independent (cf. (3.40)), and therefore so is $\delta ^2 m_{\mathrm {TB}}(\mathscr {C}_{u })$. And we have seen that this last quantity coincides with $2 \hat{E}_c$. We conclude that

5.90 $$\begin{aligned} \hat{E}_{c,II}= & {} \hat{E}_{c,I } - \frac{1}{16 \pi } \int _{S^2} {{\mathring{{\gamma }}}}^{AC }{{\mathring{{\gamma }}}}^{BD }\nonumber \\&\times \left( \frac{\varLambda }{3} \overset{{ (-3)}}{\check{h}}_{AB} \overset{{ (0)}}{\check{h}}_{CD} -\overset{{ (-1)}}{\check{h}}_{AB} \overset{{ (0)}}{\check{h}}_{CD}\right) \, d \mu _{{\mathring{{\gamma }}}} \,. \end{aligned}$$

An alternative, direct way of calculating $\hat{E}_{c,II}$ invokes the boundary integrand $b^r$ in (5.21), which now reads:

5.91 $$\begin{aligned}&r^2 b^{r}(\delta _1 g,\delta _2 g) \, \nonumber \\&\quad ={\mathring{{\gamma }}}^{AC}{\mathring{{\gamma }}}^{BD} \left[ r^3 \alpha ^2 \delta _{1} \overset{{ (2)}}{g}_{A B} \delta _{2} \overset{{ (2)}}{g}_{C D} \right. \nonumber \\&\qquad + \frac{1}{2} r^2 \left( \delta _{1} \overset{{ (2)}}{g}_{A B} \partial _{u}\delta _{2} \overset{{ (2)}}{g}_{C D} +\alpha ^2 \delta _{1} \overset{{ (2)}}{g}_{A B} \delta _{2} \overset{{ (1)}}{g}_{C D}\right. \nonumber \\&\qquad \left. +2 \alpha ^2 \delta _{1} \overset{{ (1)}}{g}_{A B} \delta _{2} \overset{{ (2)}}{g}_{C D} \right) \nonumber \\&\qquad + \frac{1}{2} r \left( \delta _{1} \overset{{ (2)}}{g}_{A B} \partial _{u} \delta _{2} \overset{{ (1)}}{g}_{C D} +\delta _{1} \overset{{ (1)}}{g}_{A B} \partial _{u}\delta _{2} \overset{{ (2)}}{g}_{C D}\right. \nonumber \\&\qquad \left. +\alpha ^2 \delta _{1} \overset{{ (1)}}{g}_{A B}\delta _{2} \overset{{ (1)}}{g}_{C D} -2\delta _{1} \overset{{ (2)}}{g}_{A B} \delta _{2} \overset{{ (2)}}{g}_{C D} \right) \nonumber \\&\qquad +\frac{1}{2} \left( \delta _{1} \overset{{ (1)}}{g}_{A B} \partial _{u}\delta _{2} \overset{{ (1)}}{g}_{C D} -\delta _{1}\overset{{ (2)}}{g}_{A B} \delta _{2} \overset{{ (1)}}{g}_{C D} \right. \nonumber \\&\qquad -2 \delta _{1}\overset{{ (1)}}{g}_{A B} \delta _{2} \overset{{ (2)}}{g}_{C D} +2\alpha ^2 \delta _{1} \overset{{ (-1)}}{g}_{A B}\delta _{2} \overset{{ (2)}}{g}_{C D} \nonumber \\&\qquad \left. \left. -\alpha ^2 \delta _{1} \overset{{ (2)}}{g}_{A B}\delta _{2} \overset{{ (-1)}}{g}_{C D} -2 \delta _{1} \overset{{ (2)}}{g}_{A B} \mathring{D}_C \delta _{2} \overset{{ (0)}}{g}_{u D} \right) \right] \nonumber \\&\qquad + o(1) \,. \end{aligned}$$

Proceedings as in the derivation of (5.32), after discarding the divergent terms both in the volume (compare (5.38)) and boundary integrals, the renormalised canonical energy in the block-diagonal gauge (II) is found to be

5.92 $$\begin{aligned}&\hat{E}_{c,II}[h,\mathscr {C}_{u }] \nonumber \\&\quad : = \left[ \frac{1}{64 \pi } \int _{\mathscr {C}_{u }} {{g}}^{BE } {{g}}^{FC}\right. \nonumber \\&\qquad \left. \times \big ( \partial _u h_{BC } \partial _{ r } h_{EF } - h_{BC } \partial _{ r } \partial _u h_{EF } \big )\,r^2 \,dr \sin \theta \, d\theta \, d\varphi \right] \Big |_{\mathrm {ren}}\nonumber \\&\qquad - {\frac{1}{64 \pi } } \int _{S^2} {{\mathring{{\gamma }}}}^{AB} {{\mathring{{\gamma }}}}^{CD}\nonumber \\&\qquad \times \left( \overset{{ (-1)}}{\check{h}}_{AC} \partial _{u} \overset{{ (-1)}}{\check{h}}_{BD} - { \overset{{ (0)}}{\check{h}}{}_{AC} \overset{{ (-1)}}{\check{h}}{}_{BD}} -\overset{{ (0)}}{\check{h}}{}_{AC} \mathring{D}_{B} \mathring{D}^{F} \overset{{ (-1)}}{\check{h}}{}_{FD}\right. \nonumber \\&\qquad \left. -2 \overset{{ (-1)}}{\check{h}}{}_{AC} \overset{{ (0)}}{\check{h}}{}_{BD} +\alpha ^{2}(2 \overset{{ (-3)}}{\check{h}}{}_{AC} \overset{{ (0)}}{\check{h}}{}_{BD}- \overset{{ (0)}}{\check{h}}{}_{AC} \overset{{ (-3)}}{\check{h}}{}_{BD}) \right) \nonumber \\&\qquad \quad \times \sin \theta \, d\theta \, d\varphi \,. \end{aligned}$$

(Here one could use (2.144) to analyse the change of the volume integral under changes of gauges to isolate the divergent terms.)

The energy flux for the renormalised energy $\hat{E}_{c,II}[h,\mathscr {C}_{u }]$ is

5.93 $$\begin{aligned}&\frac{d \hat{E}_{c,II}[h,\mathscr {C}_{u }]}{du} = - {\frac{1}{32 \pi } } \int _{S^2} {{\mathring{{\gamma }}}}^{AB}{{\mathring{{\gamma }}}}^{CD}\nonumber \\&\quad \times \left( \partial _{u} \overset{{ (-1)}}{\check{h}}_{AC} \partial _{u} \overset{{ (-1)}}{\check{h}}_{BD} - { 2 \partial _{u}\overset{{ (-1)}}{\check{h}}{}_{AC}} \overset{{ (0)}}{\check{h}}{}_{BD}\right. \nonumber \\&\qquad +\alpha ^{2}\left( 2 \partial _{u} \overset{{ (-3)}}{\check{h}}{}_{AC}\overset{{ (0)}}{\check{h}}{}_{BD}-\overset{{ (-1)}}{\check{h}}{}_{AC}\overset{{ (-1)}}{\check{h}}{}_{BD}- \overset{{ (-1)}}{\check{h}}{}_{AC} \mathring{D}_{B}\mathring{D}^{F}\overset{{ (-1)}}{\check{h}}{}_{FD} \right) \nonumber \\&\qquad \left. -\alpha ^{4} \overset{{ (-1)}}{\check{h}}{}_{AC} \times \overset{{ (-3)}}{\check{h}}{}_{BD} \right) \, \sin \theta \, d\theta \, d\varphi \,,\nonumber \\ \end{aligned}$$

where we have used (5.91) and $\partial _{u} \overset{{ (0)}}{\check{h}}{}_{AB}=\alpha ^{2}\overset{{ (-1)}}{\check{h}}{}_{AB}$.

#### Comparing with [20]

Compère et al. [20, 21] proposed a version of mass in the non-linear theory, using the asymptotically block-diagonal gauge. In [20, Equation (2.39)] they define

5.94 $$\begin{aligned} M^{(\varLambda )} = M + \frac{1}{16} (\partial _u + l)(C_{CD}C^{CD}) \,, \end{aligned}$$

where *M* is an integration constant which appears when analysing the characteristic constraint equations, *l* is a gauge field which can be set to zero and $C_{AB}$ is, essentially, our field $\overset{{ (-1)}}{\check{h}}_{AB}$. They propose to define a mass, which we will denote by $E^{(\varLambda )}$, as

5.95 $$\begin{aligned} E^{(\varLambda )} {:}{=} \frac{1}{4\pi } \int M^{(\varLambda )} \,d \mu _{{\mathring{{\gamma }}}} \,. \end{aligned}$$

(Strictly speaking, a different multiplicative constant factor is probably used in [20].) Using our notation, the quadratised version of (5.94) reads

5.96 $$\begin{aligned} \delta ^2 M^{(\varLambda )} = -\frac{1}{2} \delta ^2 \overset{{ (0)}}{V} + \frac{1}{8} \partial _u \left( {{\mathring{{\gamma }}}}^{AB}{{\mathring{{\gamma }}}}^{CD}\overset{{ (-1)}}{\check{h}}_{AC}\overset{{ (-1)}}{\check{h}}_{BD}\right) \,. \end{aligned}$$

Integrating over $S^2$ we find:

5.97 $$\begin{aligned}&\delta ^2 E^{(\varLambda )} {:}{=} \frac{1}{4\pi } \int _{S^2} \delta ^2 M^{(\varLambda )} \, d \mu _{{\mathring{{\gamma }}}} \end{aligned}$$

5.98 $$\begin{aligned}&\quad = \delta ^2 m_{\mathrm {TB}}(\mathscr {C}_{u }) + \frac{1}{16 \pi } \int _{S^{2}} {{\mathring{{\gamma }}}}^{AE }{{\mathring{{\gamma }}}}^{FB }\overset{{ (-1)}}{\check{h}}_{E F} \partial _{u} \overset{{ (-1)}}{\check{h}}_{AB} \,d \mu _{{\mathring{{\gamma }}}} \end{aligned}$$

5.99 $$\begin{aligned}&\quad = 2 \hat{E}_{c,I} + \frac{1}{16 \pi } \int _{S^{2}} {{\mathring{{\gamma }}}}^{AE }{{\mathring{{\gamma }}}}^{FB }\overset{{ (-1)}}{\check{h}}_{E F} \partial _{u} \overset{{ (-1)}}{\check{h}}_{AB} \,d \mu _{{\mathring{{\gamma }}}} \end{aligned}$$

5.100 $$\begin{aligned}&\quad = 2 \hat{E}_{c,II} + \frac{1}{16 \pi } \int _{S^{2}} {{\mathring{{\gamma }}}}^{AE }{{\mathring{{\gamma }}}}^{FB }\overset{{ (-1)}}{\check{h}}_{E F} \partial _{u} \overset{{ (-1)}}{\check{h}}_{AB} \,d \mu _{{\mathring{{\gamma }}}} \nonumber \\&\qquad +\frac{1}{8 \pi } \int _{S^2} {{\mathring{{\gamma }}}}^{AC }{{\mathring{{\gamma }}}}^{BD }\left( \frac{\varLambda }{3} \overset{{ (-3)}}{\check{h}}_{AB} \overset{{ (0)}}{\check{h}}_{CD} -\overset{{ (-1)}}{\check{h}}_{AB} \overset{{ (0)}}{\check{h}}_{CD}\right) \,\nonumber \\&\qquad \times d \mu _{{\mathring{{\gamma }}}} \,, \end{aligned}$$

where we used

5.101 $$\begin{aligned} \hat{E}_{c,I }= & {} \frac{1}{2} \delta ^2 m_{\mathrm {TB}}(\mathscr {C}_{u }) = - \frac{1}{16 \pi } \int _{S^2} \delta ^2 \overset{{ (0)}}{V} \, d \mu _{{\mathring{{\gamma }}}} \,, \end{aligned}$$

5.102 $$\begin{aligned} \hat{E}_{c,II}= & {} \hat{E}_{c,I} -\frac{1}{16 \pi } \int _{S^2} {{\mathring{{\gamma }}}}^{AC }{{\mathring{{\gamma }}}}^{BD }\nonumber \\&\times \left( \frac{\varLambda }{3} \overset{{ (-3)}}{\check{h}}_{AB} \overset{{ (0)}}{\check{h}}_{CD} -\overset{{ (-1)}}{\check{h}}_{AB} \overset{{ (0)}}{\check{h}}_{CD}\right) \, d \mu _{{\mathring{{\gamma }}}} \,. \nonumber \\ \end{aligned}$$

For completeness we note the following flux formula

5.103 $$\begin{aligned}&\partial _u \int _{S^{2}} \delta ^2 M^{(\varLambda )} d \mu _{{\mathring{{\gamma }}}} = - \frac{1}{24} \int _{S^{2}} \overset{{ (-1)}}{\check{h}}_{E F} {{\mathring{{\gamma }}}}^{AE } {{\mathring{{\gamma }}}}^{FB } \nonumber \\&\qquad \times \left\{ -\varLambda ^2\overset{{ (-3)}}{\check{h}}_{A B}-3 \partial ^{2}_{u} \overset{{ (-1)}}{\check{h}}_{AB}\right. \nonumber \\&\qquad \left. - \varLambda \left[ \mathring{D}_{(A} \mathring{D}^{C}\overset{{ (-1)}}{\check{h}}_{B) C} - \overset{{ (-1)}}{\check{h}}_{AB}\right] \right\} d \mu _{{\mathring{{\gamma }}}} \nonumber \\&\quad =\frac{1}{8} \int _{S^{2}} \partial _{u} \left[ \overset{{ (-1)}}{\check{h}}_{E F} {{\mathring{{\gamma }}}}^{AE }{{\mathring{{\gamma }}}}^{FB }\partial _{u} \overset{{ (-1)}}{\check{h}}_{AB} \right] d \mu _{{\mathring{{\gamma }}}}\nonumber \\&\qquad -\frac{1}{8}\int _{S^{2}} {{\mathring{{\gamma }}}}^{AC} {{\mathring{{\gamma }}}}^{BD} \left( \partial _{u} \overset{{ (-1)}}{\check{h}}_{AB} \partial _{u} \overset{{ (-1)}}{\check{h}}_{CD}\right. \nonumber \\&\qquad -\frac{\varLambda }{3}(\overset{{ (-1)}}{\check{h}}_{AB} \mathring{D}_{C}\mathring{D}^{F} \overset{{ (-1)}}{\check{h}}_{FD}-\overset{{ (-1)}}{\check{h}}_{AB} \overset{{ (-1)}}{\check{h}}_{CD}) \nonumber \\&\qquad \left. -\frac{\varLambda ^{2}}{3} \overset{{ (-1)}}{\check{h}}_{AB} \overset{{ (-3)}}{\check{h}}_{CD}\right) d \mu _{{\mathring{{\gamma }}}} \, . \end{aligned}$$

### $\varLambda <0$

All our results so far are independent of the sign of $\varLambda$: it suffices to replace $\alpha ^2$ by $-\alpha ^2$ wherever relevant.

Now, it should be pointed out that there is a key conceptual difference arising from the causal character of the boundary at infinity. While in the $\varLambda \ge 0$ case the data on the initial light cone determine the remainder of the evolution uniquely, this is not the case anymore for $\varLambda <0$, where we have the freedom to add *and control* boundary data on $\mathscr {I}$ (compare [30, 34]).

In the nonlinear theory the “pre-holographic” approach is to require that the conformal boundary at infinity is the same as that of anti de Sitter spacetime. At a linearised level, this translates to the requirement that the evolution preserves the condition

5.104 $$\begin{aligned} \overset{{ (2)}}{h}_{\mu \nu } \equiv 0 \,, \end{aligned}$$

up to gauge. This, in turn, leads to corner conditions on the data at the intersection of the initial light cone with the conformal boundary at infinity. For instance, choosing a gauge so that (5.104) holds, Equation (4.30) implies

5.105 $$\begin{aligned} \overset{{ (-1)}}{\check{h}}_{AB} \equiv 0 \,, \end{aligned}$$

which together with (5.104) shows that the canonical energy $E_c [\mathscr {C}_{u },h]$ is finite, *u*-independent, and equals the Trautman–Bondi mass, without the need for renormalisation.

A natural option is to relax (5.104) by allowing the asymptotic data (but *not* the whole linearised solution) to be stationary. For this we reanalyze the linearised vacuum Einstein equation under the assumption that $X=\partial _{u}$ is a Killing field for a leading terms in metric perturbation. In other words, we assume

5.106 $$\begin{aligned} {\mathcal {L}}_{X} \overset{{ (2)}}{h}_{\mu \nu } \equiv 0 \, . \end{aligned}$$

In the off-diagonal gauge this the same as

5.107 $$\begin{aligned} \partial _{u} \overset{{ (2)}}{h}_{u A} = 0 = \overset{{ (2)}}{h}_{AB}\, . \end{aligned}$$

Equation (4.41) then gives

5.108 $$\begin{aligned} \partial _{u}\overset{{ (1)}}{h}_{uu} = 0 \, . \end{aligned}$$

Applying a *u*-derivative to (4.52) and (4.54) we obtain

5.109 $$\begin{aligned}&\partial _{u} \overset{{ (0)}}{h}_{u A} = 0 \, , \end{aligned}$$

5.110 $$\begin{aligned}&4 \alpha ^2 \partial _{u} \overset{{ (-2)}}{h}_{u A} +\partial _{u}\mathring{D}_{A} \overset{{ (-1)}}{h}_{u u} - 3 \partial _{u}^2 \overset{{ (-1)}}{h}_{u A} = 0 \, . \end{aligned}$$

Under (5.107) the mass-loss formula (5.51) reduces, in view of (5.1095.110), to the one known from the $\varLambda =0$ case:

5.111 $$\begin{aligned}&\frac{d \hat{E}_c[h,\mathscr {C}_{u,R}]}{du} \nonumber \\&\quad = - {\frac{1}{32 \pi } } \int _{S^2} {{\mathring{{\gamma }}}}^{AB} {{\mathring{{\gamma }}}}^{CD} \partial _{u} \overset{{ (-1)}}{\check{h}}_{AC} \partial _{u} \overset{{ (-1)}}{\check{h}}_{BD} \, \sin \theta \, d\theta \, d\varphi . \nonumber \\ \end{aligned}$$

For completeness we list some further simplifications which arise in the asymptotics of the field. Equation (4.52) gives

5.112 $$\begin{aligned} \partial _{u} D^{A} \overset{{ (1)}}{h}_{A B} = 0 \, . \end{aligned}$$

Using (4.46) and (5.112) one obtains the following formula for the evolution of the linearised mass aspect function (not to be confused with the evolution of the quadratised mass aspect, which is relevant for (5.111)):

5.113 $$\begin{aligned} \partial _{u} \overset{{ (-1)}}{h}_{u u}= 3\alpha ^2 \overset{{ (-2)}}{h}_{u u} \, . \end{aligned}$$

Equations (5.1095.110) and (4.43) lead to

5.114 $$\begin{aligned} \partial _{u} \overset{{ (-1)}}{h}_{u u} = \alpha ^2 \overset{{ (-2)}}{h}_{u u} -\alpha ^2 \mathring{D}^{A} \overset{{ (-1)}}{h}_{u A} \, . \end{aligned}$$

Comparing (5.113) with (5.114) we find

5.115 $$\begin{aligned} \overset{{ (-2)}}{h}_{u u} = \frac{1}{2} \mathring{D}^{A} \overset{{ (-1)}}{h}_{u A} \, . \end{aligned}$$

The asymptotic symmetries of interest are now those preserving (5.107). Equations (3.43), (5.108)–(5.1095.110) and (5.112) show that these satisfy

5.116 $$\begin{aligned} \partial _u h_{uu} : \qquad 0= & {} \partial _u ( \mathring{D}_{B} \partial _{{u}}{\xi }^{B}+\alpha ^{2} {\varDelta _{{{\mathring{{\gamma }}}}}}{\xi }^{{u}}) \end{aligned}$$

5.117 $$\begin{aligned}= & {} \partial _u(2\partial _{{u}}{\xi }^{{u}} - \mathring{D}_{B}{\xi }^{B}) \,, \end{aligned}$$

5.118 $$\begin{aligned} \partial _u h_{uA} : \qquad 0= & {} \partial _{A} \big ({\varDelta _{{{\mathring{{\gamma }}}}}}\partial _u{\xi }^{{u}}+ 2 \partial _u\xi ^{u}\big ) \end{aligned}$$

5.119 $$\begin{aligned}= & {} \partial _u \varDelta _{{\mathring{{\gamma }}}}( \mathring{D}_{B} {\xi }^{B}-2\partial _{u} \xi ^{u}) \end{aligned}$$

5.120 $$\begin{aligned}= & {} \partial _u({{\mathring{{\gamma }}}}_{AB}\partial _{{u}} {\xi }^{B}+\alpha ^{2}\partial _{A}\xi ^{u}) \,, \end{aligned}$$

5.121 $$\begin{aligned} \partial _uh_{AB} :\qquad 0= & {} \partial _u ( \mathring{{\mathcal {L}}}_{{\xi }} {{\mathring{{\gamma }}}}_{AB} - {{\mathring{{\gamma }}}}_{AB} \mathring{D}_{C} {\xi }^{C} ) \,. \end{aligned}$$

Equations (5.117) and (5.119) are automatically satisfied by (3.44). Equation (5.118) shows that $\partial _u {\xi }^u$ is a linear combination of $\ell =0$ and $\ell =1$ spherical harmonics, and thus its gradient is a conformal Killing vector on $S^2$. Equation (5.120) gives

5.122 $$\begin{aligned} \partial _{{u}}^2 {\xi }^{A}= - \alpha ^2\mathring{D}^{A}\partial _u\xi ^{u} \,, \end{aligned}$$

and (5.116) follows automatically by taking the divergence of (5.122). By (5.121) the vector field $\partial _u {\xi }^A$ is also a conformal vector field on $S^2$.

By (5.56) the right-hand side of (5.111) is gauge-invariant under the current asymptotic symmetries. We conclude that

$$\begin{aligned} \hat{E}_c[h,\mathscr {C}_{u}] \equiv \hat{E}_{c,I}[h,\mathscr {C}_{u}] \end{aligned}$$

is gauge-invariant up do the addition of a functional which is *u*-independent.

Note that the flux of energy seems to be of more interest than the energy itself, since energy is always defined up to an additive constant anyway, so gauge-invariance of the flux is the key for a physically meaningful quantity.

Let us return to (5.98)-(5.99):

5.123 $$\begin{aligned}&\delta ^2 E^{(\varLambda )}= \delta ^2 m_{\mathrm {TB}}(\mathscr {C}_{u }) \nonumber \\&\qquad + \frac{1}{16 \pi } \int _{S^{2}} {{\mathring{{\gamma }}}}^{AE }{{\mathring{{\gamma }}}}^{FB }\overset{{ (-1)}}{\check{h}}_{E F} \partial _{u} \overset{{ (-1)}}{\check{h}}_{AB} \,d \mu _{{\mathring{{\gamma }}}} \end{aligned}$$

5.124 $$\begin{aligned}&\quad = 2 \hat{E}_{c,I} + \frac{1}{16 \pi } \int _{S^{2}} {{\mathring{{\gamma }}}}^{AE }{{\mathring{{\gamma }}}}^{FB }\overset{{ (-1)}}{\check{h}}_{E F} \partial _{u} \overset{{ (-1)}}{\check{h}}_{AB} \,d \mu _{{\mathring{{\gamma }}}} \,. \nonumber \\ \end{aligned}$$

The first equation is quite general, while the second holds in a Bondi gauge which is “as regular at the origin as can be”. But since $d\hat{E}_{c,I} /du$ is gauge-independent, the flux of $\hat{E}_{c,I}$ coincides with that of $\delta ^2 m_{\mathrm {TB}}(\mathscr {C}_{u })/2$.

Next, consider the explicit integral term in (5.123). Under a residual gauge transformation we have, by (3.43) and by the above,

5.125 $$\begin{aligned} \overset{{ (-1)}}{\check{h}}_{AB}&\mapsto \overset{{ (-1)}}{\check{h}}_{AB} - 2 \mathring{D}_{A}\mathring{D}_{B} {\xi }^{{u}} + {{\mathring{{\gamma }}}}_{AB} {\varDelta _{{{\mathring{{\gamma }}}}}}{\xi }^{{u}} \nonumber \\ \partial _{u} \overset{{ (-1)}}{\check{h}}_{AB}&\mapsto \partial _{u} \left( \overset{{ (-1)}}{\check{h}}_{AB} - 2 \mathring{D}_{A}\mathring{D}_{B} {\xi }^{{u}} + {{\mathring{{\gamma }}}}_{AB} {\varDelta _{{{\mathring{{\gamma }}}}}}{\xi }^{{u}} \right) \nonumber \\&= \partial _{u} \overset{{ (-1)}}{\check{h}}_{AB} \,. \end{aligned}$$

Hence

5.126 $$\begin{aligned}&\frac{1}{16 \pi } \int _{S^{2}} {{\mathring{{\gamma }}}}^{AE }{{\mathring{{\gamma }}}}^{FB }\overset{{ (-1)}}{\check{h}}_{E F} \partial _{u} \overset{{ (-1)}}{\check{h}}_{AB} \,d \mu _{{\mathring{{\gamma }}}} \nonumber \\&\quad \mapsto \frac{1}{16 \pi } \int _{S^{2}} {{\mathring{{\gamma }}}}^{AE }{{\mathring{{\gamma }}}}^{FB }\left( \overset{{ (-1)}}{\check{h}}_{E F}- 2 \mathring{D}_{E}\mathring{D}_{F} {\xi }^{{u}} \right) \partial _{u} \overset{{ (-1)}}{\check{h}}_{AB} d \mu _{{\mathring{{\gamma }}}}\nonumber \\&\quad = \frac{1}{16 \pi } \int _{S^{2}} \left( \overset{{ (-1)}}{\check{h}}{}^{AB}- 2{\xi }^{{u}} \mathring{D}^{A} \mathring{D}^{B} \right) \partial _{u} \overset{{ (-1)}}{\check{h}}_{AB} \,d \mu _{{\mathring{{\gamma }}}} \nonumber \\&\quad = \frac{1}{16 \pi } \int _{S^{2}} {{\mathring{{\gamma }}}}^{AE }{{\mathring{{\gamma }}}}^{FB }\overset{{ (-1)}}{\check{h}}_{E F} \partial _{u} \overset{{ (-1)}}{\check{h}}_{AB} \,d \mu _{{\mathring{{\gamma }}}} \,. \end{aligned}$$

Since $\delta ^2 m_{\mathrm {TB}}(\mathscr {C}_{u })$ is invariant under all residual gauge-transformations, we conclude that both $\delta ^2 m_{\mathrm {TB}}(\mathscr {C}_{u })$ and $\delta ^2 E^{(\varLambda )}$ are invariant under asymptotic symmetries preserving (5.106). But their fluxes differ. Which of the two fluxes is more relevant for specific physical applications requires further justification.

## Data Availability

This manuscript has no associated data or the data will not be deposited. [Authors’ comment: All concepts and logical implications are given in the manuscript.]

## A The conformal Killing operator on $S^2$

Consider the conformal Killing equation with a source on a round two-dimensional sphere,

A.1 $$\begin{aligned} \mathring{D}_{A} \xi _{B} + \mathring{D}_{B} \xi _{A}- \mathring{\gamma }_{AB}\mathring{D}^{C} \xi _{C}= H_{AB} \, . \end{aligned}$$

Its properties have been extensively analysed by Boucetta in [10], see also Appendix A in [23] and Appendix E in [38]). The CKV operator, defined by the left-hand side of (A.1), has a six-dimensional kernel, see (A.6). Solutions of (A.1) are not unique and exist if and only if the symmetric traceless tensor $H_{AB}$ is orthogonal to the kernel.

We calculate the kernel of the CKV operator

A.2 $$\begin{aligned} 0= & {} \mathring{D}_{A} \xi _{B} + \mathring{D}_{B} \xi _{A}- \mathring{\gamma }_{AB}\mathring{D}^{C} \xi _{C} \nonumber \\= & {} \mathring{D}^{A}\mathring{D}_{A} \xi _{B} + \mathring{D}^{A}\mathring{D}_{B} \xi _{A}- \mathring{\gamma }_{AB}\mathring{D}^{A}\mathring{D}^{C} \xi _{C} \nonumber \\= & {} \mathring{D}^{A}\mathring{D}_{A} \xi _{B}+\xi _{C}{{R^{C}}_{AB}}^{A} \nonumber \\= & {} (\varDelta _{{{\mathring{{\gamma }}}}}+1)\xi _{B} \, . \end{aligned}$$

In the last line, we have used

A.3 $$\begin{aligned} {{R^{C}}_{AB}}^{A} = {R^{C}}_{B} = {\delta ^{C}}_{B} \, . \end{aligned}$$

We wish to show that only a gradient of a “dipole function”, i.e. a combination of $\ell =1$ spherical harmonics, together with a co-gradient of a dipole function fulfill (A.2). According to the Hodge–Kodaira theorem, each one-form can be expressed as the sum of an exterior derivative, a coderivative and a harmonic form. Furthermore, it is well-known that there are no harmonic one-forms on $S^{2}$. This leads to the following decomposition of $\xi _{B}$:

A.4 $$\begin{aligned} \xi _{B}=\mathring{D}_{B}\iota +{\varepsilon _{B}}^{C} \mathring{D}_{C} \upsilon \,, \end{aligned}$$

where ${\varepsilon _{B}}^{C}$ is the two-dimensional Levi–Civita tensor. Inserting this into (A.2) one obtains

A.5 $$\begin{aligned} 0= & {} (\varDelta _{{{\mathring{{\gamma }}}}}+1)(\mathring{D}_{B}\iota +{\varepsilon _{B}}^{C} \mathring{D}_{C} \upsilon ) \nonumber \\= & {} \mathring{D}_{B}(\varDelta _{{{\mathring{{\gamma }}}}}+2)\iota +{\varepsilon _{B}}^{C} \mathring{D}_{C}(\varDelta _{{{\mathring{{\gamma }}}}}+2)\upsilon \, . \end{aligned}$$

Taking a divergence and codivergence of (A.5) gives

A.6 $$\begin{aligned} \varDelta _{{{\mathring{{\gamma }}}}}(\varDelta _{{{\mathring{{\gamma }}}}}+2)\iota = 0 = \varDelta _{{{\mathring{{\gamma }}}}}(\varDelta _{{{\mathring{{\gamma }}}}}+2)\upsilon \, . \end{aligned}$$

Basic facts about spherical harmonics show that the kernel of $\varDelta _{{{\mathring{{\gamma }}}}}(\varDelta _{{{\mathring{{\gamma }}}}}+2)$ is a mono-dipole function. Since a constant part of $\iota$ or $\upsilon$ does not contribute to the co-vector $\xi _{B}$, we conclude that conformal Killing vectors of $S^2$ can be uniquely written in the form (A.4) where $\iota$ and $\upsilon$ are linear combinations of $\ell =1$ spherical harmonics.

## B The field $b^{\alpha }$

### B.1 Alternative representation

Recall that

B.1 $$\begin{aligned} b^\alpha (\delta _1 g, \delta _2 g){:}{=} P^{ \alpha (\beta \gamma ) \delta (\epsilon \sigma ) } \delta _1 g_{\beta \gamma } \nabla _{ \delta } \delta _2 g_{\epsilon \sigma } \,, \end{aligned}$$

where

B.2 $$\begin{aligned} P^{\alpha \beta \gamma \delta \epsilon \sigma }= & {} \frac{1}{2} \big ( {{g}}^{\alpha \epsilon } {{g}}^{\delta \beta } {{g}}^{\gamma \sigma } + {{g}}^{\alpha \epsilon } {{g}}^{\sigma \beta } {{g}}^{\gamma \delta } - {{g}}^{\alpha \delta } {{g}}^{\beta \epsilon } {{g}}^{\sigma \gamma } \nonumber \\&- {{g}}^{\alpha \beta } {{g}}^{\gamma \delta } {{g}}^{\epsilon \sigma } - {{g}}^{\beta \gamma } {{g}}^{\alpha \epsilon } {{g}}^{\sigma \delta } + {{g}}^{\beta \gamma } {g}^{\alpha \delta } {{g}}^{\epsilon \sigma } \big ) \,.\nonumber \\ \end{aligned}$$

In Bondi gauge for *g* and $\delta g$ we have

B.3 $$\begin{aligned} g^{\alpha \beta } \delta g_{\alpha \beta } = 2 g^{ur} \delta g_{ur} + \underbrace{g^{AB} \delta g_{AB}}_{0} \,. \end{aligned}$$

For solutions of interest here it holds that $\delta g_{ur}\equiv 0$, which leads us to consider the gauge

B.4 $$\begin{aligned} g^{\alpha \beta } \delta g_{\alpha \beta } \equiv 0 \,, \end{aligned}$$

hence also

B.5 $$\begin{aligned} g^{\alpha \beta }\nabla _\sigma \delta g_{\alpha \beta } \equiv 0 \,. \end{aligned}$$

After insertion in (B.1) the last three terms of (B.2) drop out when (B.4)–(B.5) hold, and we obtain

B.6 $$\begin{aligned}&b^\alpha (\delta _1 g, \delta _2 g) \nonumber \\&\quad = P^{ \alpha (\beta \gamma ) \delta (\epsilon \sigma ) } \delta _1 g_{\beta \gamma } \nabla _{ \delta } \delta _2 g_{\epsilon \sigma } \nonumber \\&\quad = \frac{1}{2} \big ( {{g}}^{\alpha \epsilon } {{g}}^{\delta \beta } {{g}}^{\gamma \sigma } + {{g}}^{\alpha \epsilon } {{g}}^{\sigma \beta } {{g}}^{\gamma \delta } - {{g}}^{\alpha \delta } {{g}}^{\beta \epsilon } {{g}}^{\sigma \gamma }\big ) \delta _1 g_{\beta \gamma }\nonumber \\&\qquad \times \nabla _{ \delta } \delta _2 g_{\epsilon \sigma } \nonumber \\&\quad = \frac{1}{2} \big ( 2{{g}}^{\alpha \epsilon } {{g}}^{\delta \beta } {{g}}^{\gamma \sigma } - {{g}}^{\alpha \delta } {{g}}^{\beta \epsilon } {{g}}^{\sigma \gamma }\big ) \delta _1 g_{\beta \gamma }\nabla _{ \delta } \delta _2 g_{\epsilon \sigma } \,. \end{aligned}$$

This formula can be used as a starting point for a simpler calculation of a *u*+*r*+angles decomposed form of the fields $b^\alpha$ and $\omega ^\alpha$.

### B.2 The field $b^r$

We assume the Bondi form of the metric

B.7 $$\begin{aligned} g_{\alpha \beta }dx^{\alpha }dx^{\beta }= & {} -\frac{V}{r}e^{2\beta } du^2-2 e^{2\beta }dudr \nonumber \\&+g_{AB}\Big (dx^A-U^Adu\Big )\Big (dx^B-U^Bdu\Big ) \, , \nonumber \\ \end{aligned}$$

with variations satisfying

B.8 $$\begin{aligned} \delta g_{rr} = \delta g_{rA} = 0 \,. \end{aligned}$$

The metric allows a more general form of two-dimensional metric than the one used in (4.1). In other words, we use general coordinates adapted to a family of null hypersurfaces $\{u=\mathrm{const}\}$. In particular we do *not* assume the trace condition $g^{AB}\delta g_{AB} =0$ and that the determinant $\det g_{AB}$ takes a canonical form. Using the definition (2.138), we then find

B.9 $$\begin{aligned}&b^{r}(\delta _1 g,\delta _2 g) \nonumber \\&\quad = e^{-4 \beta }\delta _{1} g_{u r} \bigg [ g^{A B} \big ( \frac{1}{2} \nabla _{u}\delta _{2}g_{A B} - \nabla _{A}\delta _{2}g_{u B} +\frac{V}{r} \nabla _{A}\delta _{2}g_{r B} \big ) \nonumber \\&\qquad +\frac{1}{2} \big ( U^{A} g^{BC}-2 U^{B}g^{AC} \big ) \nabla _{A}\delta _{2} g_{BC} \bigg ] \nonumber \\&\qquad - \frac{e^{-4 \beta }}{2} \delta _{1}g_{u u} g^{AB} \nabla _{r} \delta 2 g_{A B} \nonumber \\&\qquad +e^{-4 \beta }\delta _{1} g_{u A} \bigg [ \big ( g^{A B}U^{C} - U^{A} g^{B C} \big ) \nabla _{r} \delta _{2}g_{B C} \nonumber \\&\qquad + g^{A B}\big ( \nabla _{r} \delta _{2}g_{u B } - \nabla _{u} \delta _{2}g_{r B } \big ) \nonumber \\&\qquad +g^{A B} \bigg ( \frac{e^{2 \beta }}{2} g^{C D} \nabla _{B} \delta _{2}g_{C D } - U^{C} \nabla _{C} \delta _{2}g_{r B} \bigg ) \bigg ] +\frac{e^{-2 \beta }}{2} \times \nonumber \\&\qquad \delta _{1} g_{A B} \bigg \{ g^{A B} \bigg [ e^{-2 \beta } \bigg ( U^{C} U^{D} \big ( \nabla _{C} \delta _{2}g_{r D } - \nabla _{r} \delta _{2}g_{C D } \big ) \nonumber \\&\qquad +\nabla _{u} \delta _{2}g_{u r } - \nabla _{r} \delta _{2}g_{u u } +U^{C} \big ( \nabla _{C} \delta _{2}g_{u r} \nonumber \\&\qquad -2 \nabla _{r} \delta _{2}g_{u C } +\nabla _{u} \delta _{2}g_{r C } \big ) \bigg ) \nonumber \\&\qquad +g^{C D} \bigg ( \nabla _{C} \delta _{2}g_{u D} -\nabla _{u} \delta _{2}g_{C D}\nonumber \\&\qquad +\frac{V}{r} \big ( \nabla _{r} \delta _{2}g_{C D}-\nabla _{C} \delta _{2}g_{r D} \big ) \bigg ) \bigg ] \nonumber \\&\qquad +2 e^{-2 \beta } U^{A} \bigg [ g^{B C} \big ( \nabla _{r} \delta _{2}g_{u C} - \nabla _{u} \delta _{2}g_{r C} +U^{D} \nabla _{r} \delta _{2}g_{C D} \big ) \nonumber \\&\qquad -g^{B D} U^{C} \nabla _{C} \delta _{2}g_{r D} - \frac{1}{2}U^{B} g^{CD} \nabla _{r} \delta _{2}g_{C D} \bigg ] \nonumber \\&\qquad +g^{A C} g^{B D} \bigg ( \nabla _{u} \delta _{2}g_{C D} - 2 \nabla _{C} \delta _{2}g_{u D} \nonumber \\&\qquad - \frac{V}{r} \big ( \nabla _{r} \delta _{2}g_{C D} - 2 \nabla _{C} \delta _{2}g_{r D} \big ) \bigg ) \nonumber \\&\qquad +\big ( U^{A} g^{B D} g^{C E} -g^{A C} g^{B D} U^{E} \big )\nabla _{C} \delta _{2}g_{D E} \bigg \} \; . \end{aligned}$$

Letting ${D}$ be the covariant derivative associated with the metric $g_{AB}$, we find

B.10 $$\begin{aligned}&b^{r}(\delta _1 g,\delta _2 g) \nonumber \\&\quad =\frac{e^{-4 \beta }}{4} \delta _{1} g_{ur} \bigg \{\delta _{2}g_{A B}\bigg [ g^{AC} g^{BD}\big ( \partial _{u} g_{C D}-\frac{2 V}{r} \partial _{r} g_{C D} \big ) \bigg ] \nonumber \\&\qquad + 2 g^{A B} \partial _{u} \delta _{2}g_{A B}+ 2 \left( U^{A} g^{BC}- 2 U^{B}g^{AC}\right) {D}_{A} \delta _{2}g_{BC} \nonumber \\&\qquad +4 \left( \delta _{2} g_{ur}e^{-2 \beta } {D}_{A}U^{A} -g^{AB} {D}_{A}\delta _{2}g_{u B} \right) \bigg \} \nonumber \\&\qquad +\frac{e^{-2 \beta }}{2} \delta _{1} g_{AB} \bigg \{ g^{AB} \bigg [ 2 \delta _{2} g_{C D} U^{C} g^{D E} {D}_{E} \beta + g^{CD}\nonumber \\&\qquad \times \left( \frac{V}{r} \partial _{r}\delta _{2}g_{C D} -\partial _{u}\delta _{2}g_{C D}+{D}_{C} \delta _{2}g_{u D}+\delta _{2}g_{u C} {D}_{D} \beta \right) \nonumber \\&\qquad +e^{-2 \beta } \bigg ( U^{C} \big ( \delta _{2}g_{uD}g^{D E} \partial {r}g_{C E} -2 \partial _{r}\delta _{2}g_{uC} \nonumber \\&\qquad - \delta _{2}g_{CD}\partial _{r}U^{D} +\delta _{2}g_{u r} {D}_{C}\beta -U^{D} \partial _{r} \delta _{2}g_{C D} \big ) \nonumber \\&\qquad +\nabla _{u}\delta _{2}g_{u r}-\nabla _{r}\delta _{2}g_{u u} -\delta _{2} g_{u r} {D}_{C}U^{C} - \frac{1}{2} \delta _{2} g_{u C} \partial _{r} U^{C} \nonumber \\&\qquad +\frac{3}{2} e^{-2 \beta } \delta _{2}g_{u r}U^{C}g_{C D} \partial _{r} U^{D} \nonumber \\&\qquad +U^{C} \big ( {D}_{C} \delta _{2} g_{u r} -2\delta _{2} g_{u r} {D}_{C} \beta \nonumber \\&\qquad +\frac{1}{2} e^{-2 \beta } \delta _{2} g_{u r} U^{E} \partial _{r} g_{E C} -\frac{1}{2} \delta _{2} g_{u E} g^{E F} \partial _{r} g_{F C} \big ) \bigg ) \bigg ] \nonumber \\&\qquad + g^{A C} g^{B D} \bigg [ \partial _{u} \delta _{2} g_{C D} -\frac{V}{r} \partial _{r} \delta _{2} g_{C D} -2 {D}_{C}\delta _{2}g_{uD} \nonumber \\&\qquad \quad - U^{E} {D}_{C}\delta _{2}g_{D E} \nonumber \\&\qquad +\frac{e^{-2 \beta }}{2} \partial _{r}g_{CD} \bigg ( 3 U^{E}\delta _{2}g_{u E} +U^{E} U^{F} \delta _{2}g_{E F}\bigg ) \bigg ]\nonumber \\&\qquad +U^{A} g^{B C} g^{D E} {D}_{D}\delta _{2}g_{C E} \nonumber \\&\qquad +\frac{e^{-2 \beta }}{2}U^{A} \bigg [ \big ( U^{B}g^{C E} -2 g^{BC} U^{E} - U^{C}g^{BE} \big )g^{D F} \nonumber \\&\qquad \times \partial _{r} g_{E F} \delta _{2}g_{C D} +2\big ( 2 U^{D}g^{BC} -U^{B}g^{C D} \big ) \partial _{r} \delta _{2} g_{C D} \nonumber \\&\qquad +4g^{B C} \partial _{r} \delta _{2} g_{u C} - g^{B C} g^{D E} \partial _{r} g_{C D} \delta _{2} g_{u E} \bigg ] \nonumber \\&\qquad + \frac{e^{-2 \beta }}{2} \bigg ( g^{A C} g^{B F} U^{D} U^{E} \delta _{2} \partial _{r}g_{E F} g_{C D} \nonumber \\&\qquad \quad + g^{A E} g^{B D} U^{C} \partial _{r} g_{C D } \delta _{2} g_{u E} \bigg ) \bigg \} \nonumber \\&\qquad +\frac{e^{-4 \beta }}{4} \delta _{1}g_{u A} \bigg [ \big ( 2 U^{A} g^{B C} g^{D E} \delta _{2} g_{C E} \nonumber \\&\qquad -3(U^{E} \delta _{2} g_{C E}+\delta _{2} g_{u C}) g^{A B} g^{C D} \big )\partial _{r} g_{B D} \nonumber \\&\qquad +2 g^{AB} \big ( 2 \partial _{r} \delta _{2} g_{u B} \big ) -4 e^{-2 \beta } \partial _{r} U^{A} \delta _{r} g_{u r} \nonumber \\&\qquad +4\big ( g^{AC}U^{B}-U^{A} g^{B C} \big )\partial _{r} \delta _{2} g_{B C} \bigg ] \nonumber \\&\qquad +\frac{e^{-4 \beta }}{4} \delta _{1} g_{u u} g^{A C} \left( g^{B D} \partial _{r} g_{AB} \delta _{2} g_{C D} - 2 \partial _{r} \delta _{2} g_{A C}\right) \; ,\nonumber \\ \end{aligned}$$

where

B.11 $$\begin{aligned} \nabla _{u}\delta _{2}g_{u r}= & {} \partial _{u} \delta _{2}g_{u r} + \delta _{2}g_{u r} \big [ U^{A} {D}_{A} \beta \nonumber \\&-2 \partial _{u} \beta -\frac{e^{-2 \beta }}{2} U^{A} \partial _{r}\big ( g_{A B} U^{B} \big ) \big ] \nonumber \\&+\delta _{2}g_{u A} \big [ \frac{1}{2} g^{AB} \partial _{r} \big ( g_{B C} U^{C} \big ) -e^{2 \beta } g^{A B} {D}_{B} \beta \big ] \, ,\nonumber \\ \end{aligned}$$

B.12 $$\begin{aligned} \nabla _{r}\delta _{2}g_{u u}= & {} \partial _{r} g_{u u} +\delta _{2}g_{u A} \bigg [ g^{A B} \partial _{r}\big ( g_{B C} U^{C} \big ) -2 e^{2 \beta } g^{A B} {D}_{B} \beta \bigg ] \nonumber \\&+\delta _{2}g_{u r} \bigg [ +2 U^{A} {D}_{A} \beta +e^{-2 \beta } U^{A}g_{A B} \partial _{r} U^{B} \nonumber \\&+2 \frac{V}{r} \partial _{r} \beta - \partial _{r} \bigg (\frac{V}{r}\bigg ) \bigg ] \, . \end{aligned}$$

Assuming $U^{A}=0$ and $g^{A B} \delta g_{AB}=0$, equation (B.9) simplifies to

B.13 $$\begin{aligned}&b^{r}(\delta _1 g,\delta _2 g) \nonumber \\&\quad = e^{-4 \beta }\delta _{1} g_{u r} \bigg [ g^{A B} \big ( \frac{1}{2} \nabla _{u}\delta _{2}g_{A B} - \nabla _{A}\delta _{2}g_{u B} +\frac{V}{r} \nabla _{A}\delta _{2}g_{r B} \big ) \bigg ] \nonumber \\&\qquad - \frac{e^{-4 \beta }}{2} \delta _{1}g_{u u} g^{AB} \nabla _{r} \delta _2 g_{A B} \nonumber \\&\qquad +e^{-4 \beta }\delta _{1} g_{u A} \bigg [ g^{A B}\big ( \nabla _{r} \delta _{2}g_{u B } - \nabla _{u} \delta _{2}g_{r B } \big ) \nonumber \\&\qquad +g^{A B} \bigg ( \frac{e^{2 \beta }}{2} g^{C D} \nabla _{B} \delta _{2}g_{C D } \bigg ) \bigg ] \nonumber \\&\qquad +\frac{e^{-2 \beta }}{2} \delta _{1} g_{A B} g^{A C} g^{B D} \bigg ( \nabla _{u} \delta _{2}g_{C D} - 2 \nabla _{C} \delta _{2}g_{u D} \nonumber \\&\qquad - \frac{V}{r} \big ( \nabla _{r} \delta _{2}g_{C D} - 2 \nabla _{C} \delta _{2}g_{r D} \big ) \bigg ) \,. \end{aligned}$$

Likewise we have the following version of (B.10):

B.14 $$\begin{aligned}&b^{r}(\delta _1 g,\delta _2 g) \nonumber \\&\quad = \frac{e^{-4 \beta }}{4} \delta _{1} g_{ur} \bigg \{\delta _{2}g_{A B}\bigg [ g^{AC} g^{BD}\left( \partial _{u} g_{C D}-\frac{2 V}{r} \partial _{r} g_{C D} \right) \bigg ] \nonumber \\&\qquad + 2 g^{A B} \partial _{u} \delta _{2}g_{A B} -4g^{AB} {D}_{A}\delta _{2}g_{u B} \bigg \} \nonumber \\&\qquad +\frac{e^{-2 \beta }}{2} \delta _{1} g_{AB} g^{A C} g^{B D} \nonumber \\&\qquad \times \bigg [ + \partial _{u} \delta _{2} g_{C D} -\frac{V}{r} \partial _{r} \delta _{2} g_{C D} -2 {D}_{C}\delta _{2}g_{uD} \bigg ]+\frac{e^{-4 \beta }}{4} \nonumber \\&\qquad \times \delta _{1}g_{u A} \bigg [ -3\delta _{2} g_{u C} g^{A B} g^{C D} \partial _{r} g_{B D} +2 g^{AB} \big ( 2 \partial _{r} \delta _{2} g_{u B} \big ) \bigg ] \nonumber \\&\qquad +\frac{e^{-4 \beta }}{4} \delta _{1} g_{u u} g^{A C} \left( g^{B D} \partial _{r} g_{AB} \delta _{2} g_{C D} - 2 \partial _{r} \delta _{2} g_{A C}\right) \; .\nonumber \\ \end{aligned}$$

## C Linearised curvature

For completeness we derive here a formula for the linearised Ricci tensor, as used in the main text. While the calculation is standard, our final formulae (C.4) and (C.5) are nonstandard in that they do not involve any explicit curvature tensors at the right-hand sides.

C.1 $$\begin{aligned} \delta \varGamma ^{\mu }{}_{\nu \lambda }= & {} \frac{1}{2} g^{\mu \kappa } \big ( \nabla _{\lambda }\delta g_{\kappa \nu } +\nabla _{\nu }\delta g_{\kappa \lambda } -\nabla _{\kappa }\delta g_{\lambda \nu } \big ) ,\nonumber \\ \end{aligned}$$

C.2 $$\begin{aligned} \delta R^{\alpha }{}_{\beta \mu \nu }= & {} \nabla _{\mu } \delta \varGamma ^{\alpha }{}_{\beta \nu } -\nabla _{\nu }\delta \varGamma ^{\alpha }{}_{\beta \mu } \, ,\end{aligned}$$

C.3 $$\begin{aligned} 2 g_{\mu \alpha }\delta R^{\alpha }{}_{\nu \sigma \rho }= & {} \nabla _{\sigma } \nabla _{\rho }\delta g_{\mu \nu } -\nabla _{\rho } \nabla _{\sigma }\delta g_{\mu \nu } \nonumber \\&+\nabla _{\sigma } \nabla _{\nu }\delta g_{\mu \rho } \nonumber \\&-\nabla _{\sigma } \nabla _{\mu }\delta g_{\nu \rho } -\nabla _{\rho } \nabla _{\nu }\delta g_{\mu \sigma } \nonumber \\&+\nabla _{\rho } \nabla _{\mu }\delta g_{\nu \sigma } \, , \nonumber \\= & {} \nabla _{\sigma }\nabla _{\nu }\delta g_{\mu \rho } -\nabla _{\sigma }\nabla _{\mu }\delta g_{\nu \rho }\nonumber \\&-\nabla _{\rho }\nabla _{\nu }\delta g_{\mu \sigma } +\nabla _{\mu }\nabla _{\rho }\delta g_{\nu \sigma } \nonumber \\&-R^{\alpha }{}_{ \mu \sigma \rho } \delta g_{\alpha \nu } -R^{\alpha }{}_{\nu \sigma \rho } \delta g_{\alpha \mu }\nonumber \\&-R^{\alpha }{}_{\nu \rho \mu } \delta g_{\alpha \sigma } -R^{\alpha }{}_{\sigma \rho \mu } \delta g_{\alpha \nu } \, ,\nonumber \\ \end{aligned}$$

C.4 $$\begin{aligned} 2 \delta R_{\nu \rho }= & {} \nabla ^{\sigma }\nabla _{\nu }\delta g_{\rho \sigma } +\nabla ^{\sigma }\nabla _{\rho }\delta g_{\sigma \nu }\nonumber \\&-\nabla ^{\sigma }\nabla _{\sigma }\delta g_{\nu \rho } -g^{\mu \sigma }\nabla _{\nu }\nabla _{\rho }\delta g_{\sigma \mu } \,, \nonumber \\ \end{aligned}$$

C.5 $$\begin{aligned} 2 \delta R_{\alpha \beta } - g_{\alpha \beta } g^{\nu \rho } \delta R_{\nu \rho }= & {} \nabla ^{\mu } \nabla _{\alpha } \delta \bar{g}_{\beta \mu } +\nabla ^{\mu } \nabla _{\beta }\delta \bar{g}_{\alpha \mu }\nonumber \\&-\nabla _{\mu } \nabla ^{\mu } \delta \bar{g}_{ \alpha \beta } -g_{\alpha \beta } \nabla ^{\kappa } \nabla ^{\lambda } \delta \bar{g}_{\kappa \lambda } \, , \,,\nonumber \\ \end{aligned}$$

where $$\delta \bar{g}_{\alpha \beta } = \delta g_{\alpha \beta } - \frac{1}{2} g^{\mu \nu }\delta g_{\mu \nu } g_{\alpha \beta } $$.

The linearisation of the Einstein tensor reads

C.6 $$\begin{aligned} \delta G_{\alpha \nu } = \delta R_{\alpha \nu } - \frac{1}{2} g_{\alpha \nu } g^{\sigma \rho } \delta R_{\sigma \rho } -\frac{1}{2} R \delta g_{\alpha \nu } +\frac{1}{2}g_{\alpha \nu }\delta g_{\sigma \rho } R^{\sigma \rho } \end{aligned}$$

In order to compare the linearised Einstein equations,

C.7 $$\begin{aligned} \delta \big ( G_{\mu \nu } + \varLambda g_{\mu \nu } \big )=0 \, , \end{aligned}$$

with the Euler–Lagrange equations, we substitute (C.6) into (C.7) and regroup the terms as follows

C.8 $$\begin{aligned} \delta R_{\alpha \nu } - \frac{1}{2} g_{\alpha \nu } g^{\sigma \rho } \delta R_{\sigma \rho } = \underbrace{ \delta G_{\alpha \nu } +\frac{1}{2} R \delta g_{\alpha \nu } -\frac{1}{2}g_{\alpha \nu }\delta g_{\sigma \rho } R^{\sigma \rho } }_{\varLambda \overline{h}_{\alpha \nu } } \, . \end{aligned}$$

Using (C.5), the last equations are equivalent to (2.146) when the background satisfies $R_{\mu \nu }=\varLambda g_{\mu \nu }$ holds. Furthermore, under this last assumption, Eq.  (C.8) coincides with (4.3).

## D The remaining Einstein equations revisited

In this appendix we derive some further consequences of the linearised Einstein equations.

Adding, in vacuum, the equation $\mathring{D}^A \times$ (4.14),

D.1 $$\begin{aligned} \partial _r \left[ r^4 \partial _r(r^{-2} \mathring{D}^A {h}_{uA})\right] = r^2 \left[ \partial _r \left( r^{-2} \mathring{D}^A \mathring{D}^B {h}_{AB}\right) \right] \,, \end{aligned}$$

to $$-r^2\partial _r \times $$ (4.34),

D.2 $$\begin{aligned} 2 r^2 \partial _r^2 (r {h}_{uu}) = - r^2 \partial _r \big [r^{-2} \big ( \mathring{D}^A\mathring{D}^B {h}_{AB} - \partial _r (r^2 \mathring{D}^A {h}_{uA} \big ) \big ] \,, \end{aligned}$$

one finds

D.3 $$\begin{aligned} \partial _r\big ( r^2 \partial _r {h}_{uu} {-} \mathring{D}^A {h}_{uA} \big ) = 0 \,. \end{aligned}$$

Integrating, we find that there exists a function $\chi (u,x^{C})$ such that

D.4 $$\begin{aligned} \mathring{D}^{A} {h}_{u A} = r^2 \partial _{r} {h}_{u u} +\chi (u,x^{C}) \,. \end{aligned}$$

We evaluate this equation at $r=0$ for a globally smooth linearised field ${h}_{\mu \nu }$. Let $\zeta [h]$ denote the gauge vector field which brings $h_{\mu \nu }$ to the Bondi gauge; compare (3.9)–(3.10) with $\xi$ given by (3.37)–(3.38). By (3.26) and (3.28) one obtains

D.5 $$\begin{aligned} \chi = ( \mathring{D}^{A} {h}_{u A}- r^2 \partial _{r} {h}_{u u})|_{r=0} = - \frac{1}{2} ({\varDelta _{{{\mathring{{\gamma }}}}}}+2){\varDelta _{{{\mathring{{\gamma }}}}}}{\xi }^{{u}} \,. \end{aligned}$$

Keeping in mind that $\xi ^u$ is freely specifiable *at a chosen value of* *u*, but that $\partial _u\xi ^u$ is essentially determined by the solution (cf. (D.6) below), Equation (D.5) shows that the function $\chi$ can be made to vanish by applying an asymptotic symmetry at a chosen retarded time *u*, and that the vanishing of $\chi$ at some value of *u* will *not* be preserved by evolution in general. We will exploit the possibility of setting $\chi$ to zero at the chosen light cone later, but for the moment we remain in a general gauge compatible with our setting so far.

Equations (D.5) and (3.38) show that

D.6 $$\begin{aligned} \partial _u \chi= & {} - \frac{1}{2} ({\varDelta _{{{\mathring{{\gamma }}}}}}+2){\varDelta _{{{\mathring{{\gamma }}}}}}\partial _u {\xi }^{{u}} \nonumber \\= & {} -\frac{1}{2} ({\varDelta _{{{\mathring{{\gamma }}}}}}+2){\varDelta _{{{\mathring{{\gamma }}}}}}\left[ \frac{1}{2} h_{tt}|_{r=0} +\frac{1}{4} h_{ij}|_{r=0} \right. \nonumber \\&\left. \times \left( \delta ^{ij} - 3 \frac{x^ix^j}{r^2}\right) + \frac{1}{2} \mathring{D}^A \xi _A[h] \right] \,. \end{aligned}$$

where we have used the fact that, by [23, Appendix A.1], when ${\xi }^{B}({u}, x^{A})$ is a conformal vector field its divergence is a linear combination of $\ell =1$ spherical harmonics.

We continue by inserting (D.4) into the right-hand side of (4.34):

D.7 $$\begin{aligned} \mathring{D}^{A} \mathring{D}^{B} {h}_{A B} = r^4 \partial ^{2}_{r} {h}_{u u} +2 r^3 \partial _{r} {h}_{u u} -2r^2 {h}_{u u} { +}2 r \chi (u, x^C) \, . \end{aligned}$$

Recall that the equation $r^2 \mathscr {E}_{u u}=0$ reads

D.8 $$\begin{aligned}&2\Big ( \partial _{u} +(\alpha ^2 r^2-1) \partial _{r} -\frac{1}{r} \Big ) \mathring{D}^{A} {h}_{u A} - \mathring{D}^{A} \mathring{D}_{A} {h}_{u u} \nonumber \\&\quad -(\alpha ^2 r^2-1) \bigg (\frac{\mathring{D}^{A} \mathring{D}^{B} {h}_{A B}}{r^2}\bigg ) -2 r \partial _{u} {h}_{u u} \nonumber \\&\quad -2 (\alpha ^2 r^2-1) \partial _{r}(r {h}_{u u}) =0 \, . \end{aligned}$$

Substituting (4.32) into (D.8) one obtains

D.9 $$\begin{aligned}&\Big ( 2 r \partial _{u} + (\alpha ^2 r^3-r) \partial _{r} -2 \alpha ^2r^2 \Big ) \mathring{D}^{A} {h}_{u A} \nonumber \\&\quad - r \mathring{D}^{A} \mathring{D}_{A} {h}_{u u}-2 r^2 \partial _{u} {h}_{u u} = 0 \, . \end{aligned}$$

We insert the asymptotic expansion of $h_{\mu \nu }$ into (D.9), obtaining

D.10 $$\begin{aligned}&r^2 \Big ( 2 \alpha ^2 {\overset{{ (-1)}}{h}}_{uu} - 2 \alpha ^2 \chi -({\varDelta _{{{\mathring{{\gamma }}}}}}+ 2) \overset{{ (1)}}{h}_{uu} \Big ) \nonumber \\&\quad + r \Big ( 6 \alpha ^2 {\overset{{ (-2)}}{h}}_{uu} - 4 \partial _{u} {\overset{{ (-1)}}{h}}_{u u} +2 \partial _{u} \chi \Big ) = O (1) \,, \end{aligned}$$

thus both the coefficient of *r* and that of $r^2$ in the left-hand side have to vanish:

D.11 $$\begin{aligned}&\displaystyle \chi = {\overset{{ (-1)}}{h}}_{uu} -\frac{1}{ 2 \alpha ^2 }({\varDelta _{{{\mathring{{\gamma }}}}}}+ 2) \overset{{ (1)}}{h}_{uu} \,, \end{aligned}$$

D.12 $$\begin{aligned}&\partial _{u} \chi = 2 \partial _{u} {\overset{{ (-1)}}{h}}_{u u} - 3 \alpha ^2 {\overset{{ (-2)}}{h}}_{uu} \,. \end{aligned}$$

Assume momentarily that we are in Minkowski spacetime. From (D.10) we obtain

D.13 $$\begin{aligned} \partial _u \chi = 2 \partial _u {\overset{{ (-1)}}{h}}_{uu} \,. \end{aligned}$$

An asymptotic expansion of the right-hand side of (D.7) gives

D.14 $$\begin{aligned} \mathring{D}^{A} \mathring{D}^{B} {h}_{A B} = 2r (\chi - {\overset{{ (-1)}}{h}}_{uu} ) +O(1) \, . \end{aligned}$$

Differentiating (D.14) with respect to *u* one finds

D.15 $$\begin{aligned} \partial _u {\overset{{ (-1)}}{h}}_{uu} = \frac{1}{2} \mathring{D}^{A} \mathring{D}^{B} \partial _{u} \overset{{ (-1)}}{\check{h}}_{AB} \,. \end{aligned}$$

This is the linearised version of the usual formula for the time evolution of the mass aspect function.

When $\varLambda \ne 0$, we can insert (D.11) into (D.14) to obtain a *corner condition*

D.16 $$\begin{aligned} ({\varDelta _{{{\mathring{{\gamma }}}}}}+ 2) \overset{{ (1)}}{h}_{uu} = -\alpha ^2 \mathring{D}^{A} \mathring{D}^{B} \overset{{ (-1)}}{\check{h}}_{A B} \, . \end{aligned}$$

Differentiating (D.11) with respect to *u* and inserting into (D.12) gives the following evolution equation for $\overset{{ (-1)}}{h}_{uu}$:

D.17 $$\begin{aligned} \displaystyle \partial _{u} {\overset{{ (-1)}}{h}}_{u u}= & {} - \frac{1}{ 2 \alpha ^2 } ({\varDelta _{{{\mathring{{\gamma }}}}}}+ 2) \partial _u \overset{{ (1)}}{h}_{uu} + 3 \alpha ^2 {\overset{{ (-2)}}{h}}_{uu} \nonumber \\= & {} \frac{1}{2} \mathring{D}^{A} \mathring{D}^{B} \partial _{u} \overset{{ (-1)}}{\check{h}}_{AB} + 3 \alpha ^2 {\overset{{ (-2)}}{h}}_{uu} \,. \end{aligned}$$

For further reference we note the following. In [37] Jezierski has introduced two gauge-invariant scalars describing linearised gravitational fields on spherically symmetric backgrounds. These scalars are closely related to the fields $\mathring{D}^B h_{uB}$ and $\varepsilon ^{A B} \mathring{D}_{A} h_{uB}$. The time evolution of these last fields is governed by the divergence and co-divergence of (4.50):

D.18 $$\begin{aligned} 0= & {} \Big [ \partial _{u} \partial _{r} + (\alpha ^2 r^2 -1) \partial _{r} ^2 -\frac{2}{r}\partial _{u} -2 \alpha ^2 \Big ] \mathring{D}^{A} {h}_{u A} \nonumber \\&+\frac{1}{r^2} \partial _{u} \mathring{D}^{A} \mathring{D}^{B} {h}_{A B} - \partial _{r} \mathring{D}^{A} \mathring{D}_{A}{h}_{u u} \,, \end{aligned}$$

D.19 $$\begin{aligned} 0= & {} \Big [ (\alpha ^2 r^2-1) r^2 \partial ^{2}_{r} +r^2 \partial _{u} \partial _{r} -2 r \partial _{u} -2 \alpha ^2 r^2 \Big ]\varepsilon ^{A B} \mathring{D}_{A} h_{uB} \nonumber \\&+\partial _{u} \big ( \varepsilon ^{A B} \mathring{D}_{A} \mathring{D}^{C} h_{C B} \big ) -{\varDelta _{{{\mathring{{\gamma }}}}}}\varepsilon ^{A B} \mathring{D}_{A} h_{uB} \, . \end{aligned}$$

## Footnotes

1 The calculation here is due to J.Hoque.

2 We take this opportunity to note that there is a misprint in the power of the log term in Equation (4.33) in [13]; the correct power is $r^{-3}$, as here. There are also some powers of $\nu ^0$ missing in this equation, which is innocuous since $\nu ^0$ equals 1 in situations of interest.

## References

[1] A. Ashtekar, B. Bonga, A. Kesavan, Asymptotics with a positive cosmological constant: I. Basic framework. Class. Quantum Gravity **32**, 025004 (2015). arXiv:1409.3816 [gr-qc]

[2] A. Ashtekar, B. Bonga, A. Kesavan, Asymptotics with a positive cosmological constant: II. Linear fields on de Sitter spacetime. Phys. Rev. D **92**, 044011 (2015). arXiv:1506.06152 [gr-qc]

[3] A. Ashtekar, B. Bonga, A. Kesavan, Asymptotics with a positive cosmological constant. III. The quadrupole formula. Phys. Rev. D **92**, 104032 (2015). arXiv:1510.05593 [gr-qc]

[4] Ashtekar A, Das S (2000). Asymptotically anti-de Sitter spacetimes: conserved quantities. Class. Quantum Gravity.

[5] Ashtekar A, Magnon A (1984). Asymptotically anti-de Sitter space-times. Class. Quantum Gravity.

[6] H. Barzegar, P.T. Chrusciel, M. Hörzinger, Energy in higher-dimensional spacetimes. Phys. Rev. D **96**(12), 124002 (2017). arXiv:1708.03122 [gr-qc]

[7] Bishop NT (2016). Gravitational waves in a de Sitter universe. Phys. Rev. D.

[8] L. Blanchet, T. Damour, Radiative gravitational fields in general relativity. I. General structure of the field outside the source. Philos. Trans. R. Soc. Lond. Ser. A **320**, 379–430 (1986)

[9] Bondi H, van der Burg MGJ, Metzner AWK (1962). Gravitational waves in general relativity VII: waves from axi-symmetric isolated systems. Proc. R. Soc. Lond. A.

[10] Boucetta M (1999). Spectre des laplaciens de Lichnerowicz sur les sphères et les projectifs réels. Publ. Mat..

[11] Chruściel PT (1985). On the relation between the Einstein and the Komar expressions for the energy of the gravitational field. Ann. Inst. Henri Poincaré.

[12] P.T. Chruściel, Sk.J. Hoque, T. Smołka, Energy of weak gravitational waves in spacetimes with a positive cosmological constant. Phys. Rev. D **103**, 064008 (2021). arXiv:2003.09548 [gr-qc] 10.1140/epjc/s10052-021-09350-y PMC8550275 34720723

[13] P.T. Chruściel, L. Ifsits, The cosmological constant and the energy of gravitational radiation. Phys. Rev. D **93**, 124075 (2016). arXiv:1603.07018 [gr-qc]

[14] Chruściel PT, Jezierski J (2012). On free general relativistic initial data on the light cone. J. Geom. Phys..

[15] P.T. Chruściel, J. Jezierski, J. Kijowski, Hamiltonian field theory in the radiating regime, Lect. Notes in Physics, vol. m70. Springer, Berlin (2002)

[16] Chruściel PT, Jezierski J, Leski S (2004). The Trautman–Bondi mass of hyperboloidal initial data sets. Adv. Theor. Math. Phys..

[17] P.T. Chruściel, J. Jezierski, M. MacCallum, Uniqueness of the Trautman–Bondi mass. Phys. Rev. D **58**, 084001 (1998). arXiv:gr-qc/9803010

[18] P.T. Chruściel, T.-T. Paetz, The many ways of the characteristic Cauchy problem. Class. Quantum Gravity **29**, 145006 (2012). arXiv:1203.4534 [gr-qc]

[19] Chruściel PT, Simon W (2001). Towards the classification of static vacuum spacetimes with negative cosmological constant. J. Math. Phys..

[20] Compère G, Fiorucci A, Ruzziconi R (2019). The $$\Lambda $$-$$\text{BMS}_4$$ group of $$dS_4$$ and new boundary conditions for AdS$$_4$$. Class. Quantum Gravity.

[21] Compère G, Fiorucci A, Ruzziconi R (2020). The $$\Lambda $$-BMS$$_4$$ Charge Algebra. JHEP.

[22] Č Crnković, E. Witten, Covariant description of canonical formalism in geometrical theories, Three hundred years of gravitation. Cambridge University Press, Cambridge, pp. 676–684 (1987)

[23] Czajka P, Jezierski J (2018). Conformal Yano–Killing tensors for space-times with cosmological constant. Acta Phys. Polon. B.

[24] Date G, Hoque SJ (2016). Gravitational waves from compact sources in a de Sitter background. Phys. Rev. D.

[25] Date G, Hoque SJ (2017). Cosmological horizon and the Quadrupole Formula in de Sitter background. Phys. Rev. D.

[26] C. Fefferman, C.R. Graham, The ambient metric. Ann. Math. Stud. **178**, 1–128 (2011). arXiv:0710.0919 [math.DG]

[27] Fischer K (1969). Interpretation of Einstein’s theory of gravitation including the cosmological term as a de Sitter-invariant field theory on the de Sitter space. Z. Phys..

[28] Friedman JL (1978). Generic instability of rotating relativistic stars. Commun. Math. Phys..

[29] Friedrich H (1986). On the existence of $$n$$-geodesically complete or future complete solutions of Einstein’s field equations with smooth asymptotic structure. Commun. Math. Phys..

[30] Friedrich H (1995). Einstein equations and conformal structure: existence of anti-de-Sitter-type spacetimes. J. Geom. Phys..

[31] de Haro S, Solodukhin SN, Skenderis K (2001). Holographic reconstruction of spacetime and renormalization in the AdS/CFT correspondence. Commun. Math. Phys..

[32] Hawking SW (1983). The boundary conditions for gauged supergravity. Phys. Lett. B.

[33] Hollands S, Ishibashi A, Marolf D (2005). Comparison between various notions of conserved charges in asymptotically AdS-spacetimes. Class. Quantum Gravity.

[34] Holzegel G, Luk J, Smulevici J, Warnick C (2019). Asymptotic properties of linear field equations in anti-de Sitter space. Commun. Math. Phys..

[35] Hoque SJ, Virmani A (2018). On propagation of energy flux in de Sitter spacetime. Gen. Relativ. Gravit..

[36] Iyer V, Wald RM (1994). Some properties of Noether charge and a proposal for dynamical black hole entropy. Phys. Rev. D.

[37] Jezierski J (1999). Energy and angular momentum of the weak gravitational waves on the Schwarzschild background—quasilocal gauge-invariant formulation. Gen. Relativ. Gravit..

[38] Jezierski J (2002). “Peeling property” for linearized gravity in null coordinates. Class. Quantum Gravity.

[39] J. Jezierski, Geometry of null hypersurfaces, in Relativity Today (Proceedings of the Seventh Hungarian Relativity Workshop, 2003), ed. by I. Racz (Akademiai Kiado, Budapest, 2004) (2003). arXiv:gr-qc/0405108

[40] Jezierski J (2008). Asymptotic conformal Yano-Killing tensors for asymptotic anti-de Sitter spacetimes and conserved quantities. Acta Phys. Polon. B.

[41] J. Kijowski, W.M. Tulczyjew, A Symplectic Framework for Field Theories, Lecture Notes in Physics, vol. 107. Springer, New York (1979)

[42] M. Kolanowski, J. Lewandowski, Energy of gravitational radiation in the de Sitter universe at the Scri and at a horizon. Phys. Rev. D **102**, 124052 (2020). arXiv:2008.13753 [gr-qc]

[43] Kolanowski M, Lewandowski J (2021). Hamiltonian charges in the asymptotically de Sitter spacetimes. JHEP.

[44] Korbicz J, Tafel J (2004). Lagrangian and Hamiltonian for the Bondi-Sachs metrics. Class. Quantum Gravity.

[45] Lee J, Wald RM (1990). Local symmetries and constraints. J. Math. Phys..

[46] Mädler T, Winicour J (2016). Bondi–Sachs formalism. Scholarpedia.

[47] Paetz T-T (2015). Conformally covariant systems of wave equations and their equivalence to Einstein’s field equations. Ann. H. Poincaré.

[48] Papadimitriou I, Skenderis K (2005). Thermodynamics of asymptotically locally AdS spacetimes. JHEP.

[49] Saw V-L (2018). Bondi mass with a cosmological constant. Phys. Rev. D.

[50] Starobinsky AA (1983). Isotropization of arbitrary cosmological expansion given an effective cosmological constant. JETP Lett..

[51] Szabados LB, Tod P (2015). A positive Bondi-type mass in asymptotically de Sitter spacetimes. Class. Quantum Gravity.

[52] Szabados LB, Tod P (2019). A review of total energy-momenta in GR with a positive cosmological constant. Int. J. Mod. Phys. D.

[53] A. Trautman, Radiation and boundary conditions in the theory of gravitation. Bull. Acad. Pol. Sci. Série Sci. Math. Astr. Phys. **VI**, 407–412 (1958). arXiv:1604.03145

[54] A. Trautman, King College Lectures on general relativity, May–June 1958. Gen. Relativ. Gravit. **34**, 715–762 (2002)

[55] Vasy A (2010). The wave equation on asymptotically de Sitter-like spaces. Adv. Math..

[56] R.M. Wald, A. Zoupas, A general definition of “conserved quantities” in general relativity and other theories of gravity. Phys. Rev. D **61**, 084027 (2000). arXiv:gr-qc/9911095

